# Activity as a readout parameter for neurobehavioral research and severity assessment in laboratory rodents: a systematic comparison and meta-analysis of measurement techniques

**DOI:** 10.1038/s41598-026-65396-6

**Published:** 2026-08-21

**Authors:** Daniel Pérez-Pérez, Miriam Maihöfner, Anna Munk, Maria Reiber, Anika Bach-Hagemann, André Bleich, Peter Gass, Christine Häger, Marie Johne, Simone Kumstel, Ute Lindauer, Anne S. Mallien, Kerstin Schwabe, Leonie Tix, René H Tolba, Laura Warner, Brigitte Vollmar, Dietmar Zechner, Heidrun Potschka

**Affiliations:** 1 Institute of Pharmacology, Toxicology, and Pharmacy, Ludwig-Maximilians- Universität München, Munich, Germany; 2 Fraunhofer Institute for Toxicology and Experimental Medicine, Department of Preclinical Pharmacology and Toxicology, Hanover, Germany; 3 Institute for Laboratory Animal Science, Hannover Medical School, Hanover, Germany; 4 Department of Psychiatry and Psychotherapy, RG Animal Models in Psychiatry, Medical Faculty Mannheim, Central Institute of Mental Health, Heidelberg University, Mannheim, Germany; 5 Institute for Laboratory Animal Science & Experimental Surgery, Faculty of Medicine, RWTH Aachen University, Aachen, Germany; 6 Rudolf-Zenker-Institute of Experimental Surgery, University Medical Center Rostock, Rostock, Germany; 7 Translational Neurosurgery and Neurobiology, Department of Neurosurgery, Faculty of Medicine, RWTH Aachen University, Aachen, Germany; 8 Experimental Neurosurgery, Department of Neurosurgery, Hannover Medical School, Hanover, Germany

**Keywords:** Neurobehavioral research, Severity assessment, Laboratory rodents, Activity monitoring, Home cage monitoring, Medical research, Neurology, Neuroscience

## Abstract

Despite the widespread use of locomotion and activity analyses in neurobehavioral research and severity assessment, comparative evidence on the sensitivity and informative value of available monitoring techniques remains limited. Our study addresses these gaps by systematically evaluating activity data from multiple techniques across different interventions and disease models. The meta-analysis of datasets from the DFG FOR2591 consortium assessed how horizontal activity and locomotion change following surgical procedures, during stress paradigms, and in non-genetic and genetic disease models. Invasive interventions consistently resulted in post-surgical declines in activity, while neurological and psychiatric models were associated either with hypo- or hyperlocomotion, highlighting the bidirectional nature of activity alterations. A comparative evaluation of the techniques revealed that continuous home-cage telemetry and video-based tracking during the dark phase were more sensitive than clinical scoring systems, Neuroscores, or Open field tests. Voluntary wheel running emerged as the most informative of the alternatives. The analysis supports the integration of automated, continuous monitoring technologies to overcome the limitations of traditional daytime and observer-dependent methods. Since horizontal activity and locomotion have proven to be valuable readout parameters for most interventions and disease models, it is recommended that a sensitive analysis be integrated into multidimensional approaches to severity assessment.

**Supplementary Information:**

The online version contains supplementary material available at 10.1038/s41598-026-65396-6.

## Introduction

Horizontal activity and locomotion are important readout parameters in neurobehavioral studies evaluating the phenotype of induced and genetically engineered neurological and neuropsychiatric disease models, as well as the impact of genetic, electrical, and pharmacological interventions.^1^ During pre-clinical drug development, a precise evaluation of drug candidates on activity patterns can provide valuable information about efficacy and tolerability, serving as a basis for informed decision-making^2,3^.

In vivo studies in experimental animals must be based on an ethical evaluation that weighs the relevance of the knowledge gained against the potential harm to the animals. In line with this concept, the EU Directive 2010/63 mandates prospective and retrospective assessments of the severity experienced by the animals as a consequence of the underlying research, classifying procedures as non-recovery, mild, moderate, or severe. Sensitive and precise objective assessment of severity provides a basis for approval decisions, for feedback loops for continuous improvement, and for evidence-based identification and validation of refinement measures. Activity and locomotion are often among the standard parameters in common clinical scoring systems.

In addition to standard clinical scoring, more advanced techniques have been developed for both research and evidence-based severity assessment. Continuous 24/7 monitoring of activity can be achieved using video-based tracking, radiotelemetric activity monitoring, or analysis of wheel-running activity within the home-cage environment^4–6^. Alternatively, activity can be assessed by transferring animals to a novel Open field (OF) arena, where their behavior is tracked over a limited period using video-based systems^7^.

In the context of severity assessment, the application of these objective quantitative technical approaches has provided evidence that the precise data generated can be valuable across different interventions and models^5,8–23^.

These findings sparked interest in applying unbiased quantitative approaches for specific questions concerning the burden of new models and in new genetically modified mouse lines, as well as the efficacy of refinement measures. However, the requirements for data management and storage, the need for handling animals for OF testing, and the need for invasive transmitter implants raise the question of whether it is worthwhile to apply these techniques in the context of severity assessment, and if so, what the gain in informative value is. Therefore, we conducted a comprehensive analysis of activity data collected from mice and rats using various techniques across different interventions and induced or genetic disease models. The analyses provide comparative information on the sensitivity and informative value of data collected using multiple methods, including scoring systems, tracking in OF arenas and home cages, radiotelemetric approaches, and automated wheel running analysis. Moreover, we identified those interventions and disease models in which changes in locomotion can serve as relevant readout parameters.

## Materials and methods

This is a retrospective data analysis aimed at comparing the sensitivity of various methods for activity assessment across different rodent models and interventions. Moreover, we identified models and interventions in which activity serves as an informative parameter for assessing severity. Studies conducted by the research group (DFG FOR259^24–28^) evaluating activity-related readout parameters in mouse and rat models, as well as in the context of experimental interventions, were included in the analysis. The readout parameters comprised activity-related parameters from routine clinical scoring systems and a Neuroscore (modified Irwin test), the distance moved in the OF test and during voluntary wheel running (VWR), and telemetric or video-based activity recordings in the home cage.

A wide range of different animal disease models and interventions from the fields of neurology, gastroenterology, and oncology were included in the data analysis. The disease models comprised both genetic and non-genetic models, with electrical, chemical, or mechanical induction (Table 1).

**Table 1:** General information regarding the included studies.

Assigned classification	Species	Study ID	Experimental/ Intervention group	Reference data	Analyses		
					ES	MA	ROC + Cor
Surgical interventions	Mice	Intracerebral Electrode Implantation	Implanted (multiple analgesic regimes)	Baseline / Naïve / Sham (drug-control)	X	X	X
		Intraperitoneal Transmitter Implantation	Implanted	Baseline	X	X	
	Rats	Subarachnoid Hemorrhage (SAH) Model	SAH	Baseline / Sham / Sham (drug-control)	X	X	
		Partial Hepatectomy (PH)	PH (50% and 70%)	Baseline / Sham	X	X	X
Neuroscientific and stress tests/techniques	Mice	Restraint Stress Model	Stressed	Baseline / Naïve	X		
	Rats	Forced Swim Test (FST)	FST	Baseline / Naïve / Sham	X	X	
		Biopotential Recording Techniques	Tethered	Baseline	X		
Non-genetic disease models	Mice	Epilepsy Model – Kindling* (hip)	Kindling	Naïve / Sham	X	X	
		Epilepsy Model – Kindling* (bla)	Kindling	Naïve / Sham	X	X	
		Epilepsy Model – Post SE (IHK)^#^	IHK	Naïve / Sham / Sham (transmitter)	X	X	
		Colitis Model (Lab 1)	DSS (1 and 1.5%)	Baseline / Sham	X		
		Colitis Model (Lab 2)	DSS	Baseline	X		
		Pancreatic Cancer Model^#^	Pancreatic cancer	Baseline	X	X	
	Rats	Epilepsy Model – Kindling* (teth)	Kindling	Naïve / Sham	X		
		Epilepsy Model – Kindling* (tele)	Kindling	Baseline / Sham / Sham (transmitter)	X	X	
		Epilepsy Model – Post SE (pilo)	Post-SE (three cohorts)	Baseline / Naïve / Sham	X	X	X
		Epilepsy Model – Post SE (electrical)	Post-SE (three cohorts)	Baseline / Naïve / Sham	X	X	X
		Hearing Loss Model	Deaf (adult and juvenile)	Baseline / Naïve / Sham	X	X	
		Parkinsonism Model^#^	Post-6OHDA	Baseline / Sham	X	X	
		Glioma Model^#^	Glioma	Baseline	X	X	
		Glioma Model (endpoint)	Glioma	Baseline	X		
		Cerebellar Cognitive Affective Syndrome Model^#^	Lesioned	Baseline / Naïve / Sham	X	X	
Genetically modified disease models	Mice	Dravet Syndrome Model	*Scn1a-A1783V* ^*−/+*^	Naïve (*Scn1a*^+/+^)	X	X	X
		*Gria1* KO Model	*Gria1* ^−/−^	Naïve (wildtype)	X	X	X
	Rats	DAT KO Model	DAT^−/−^ and DAT^+/−^	Naïve (wildtype)	X	X	

ES; Effect size; MA, Meta-analysis; ROC, Receiving operating characteristics; Cor, Correlation; hip, hippocampus; bla, basolateral amygdala; SE, Status epilepticus; IHK, intrahippocampal kainate; teth, tethered; tele, telemetry; DSS, Dextran sulfate sodium; 6OHDA, 6-Hydroxydopamine; *Scn1a*, voltage-gated sodium channel subunit 1 A; tm, transmitter; *Gria1*, GluA1 subunit of the AMPA receptors; KO, knockout; DAT, Dopamine transporter; pilo, pilocarpine.

* Please note that with our current protocol, spontaneous seizures cannot be achieved in this model; # Data collected during the post-surgical period were additionally considered “surgical interventions in rodents.”

The studies were grouped into four methodological categories according to their main intervention or modeled disease: (1) surgical interventions (e.g., intracranial electrode implantation, partial hepatectomy); (2) neuroscientific and stress tests/techniques (e.g., forced swim test, tethered and non-tethered recording techniques); (3) non-genetic disease models (e.g., colitis, epilepsy, Parkinsonism); (4) genetically modified disease models (e.g., Dravet syndrome, *Gria1* encephalitis). Please note that for some studies, data from different experimental phases have been considered for different methodological categories (e.g., data from the early phase following surgery for category 1 and data from a later disease phase for category 3).

Animals exposed to the primary intervention or developing the modeled disease were designated as the experimental/intervention group. For some studies, more than one experimental/intervention group was considered (i.e., different disease intensities, Table 1).

Our analysis compared activity data from these experimental/intervention groups with their respective reference data. Three different categories of reference data were used for comparison, including data from naïve animals, data from sham animals, and baseline data. The latter includes data from a true baseline period (i.e., pre-exposure period) or data from the end of the laboratory-specific recovery phase for telemetric analyses (Supplementary Table S1; telemetric data can only be collected following surgery). For harmonization purposes, we assigned these reference categories to individual studies, even when the reference data in the original study were not labeled accordingly (Table 1).

### Workflow for data collection, processing, and analysis

A general workflow for collecting, merging, pre-processing, visualization, analysis, and reporting of the data is illustrated in Supplementary Figure S1. To improve harmonization among studies, standardization steps were implemented at various stages for individual studies (specified below).

### Data screening and completion

Based on a survey within the research group in 2025 (DFG FOR259^24–28^), studies were identified that evaluate one or more of the selected activity-related readout parameters. We reached out to all collaborating partners to coordinate the provision and completion of respective data, as well as methodological details.

Studies with a sample size of less than five animals/experimental units per experimental group, as well as any time points or groups with more than 20% missing data, were excluded (note that model-related dropouts were expected as part of the experimental design and were not considered as missing data, e.g., due to reaching the humane endpoint during tumor progression).

### Readout parameter-specific merging and preprocessing steps

All shared data were transferred into a central file containing all the relevant readout parameters. Error-free transfer was controlled via random quality spot checks by one coworker (evaluating at least 20% of the central files). To standardize the numbering of experimental days, they were renumbered relative to the day of the experiment/intervention (day zero) or the respective day for reference data.

#### Clinical score

The respective local authorities approved each site- and model-specific clinical scoring system used to assess the animals’ severity during the experiments. Data from these study-specific clinical scoring systems were used for the analysis. Information about the selected activity-related parameters in each clinical scoring system is provided in the Supplementary Table S2. For comparison with other techniques, we used the Clinical Scores evaluated on the same day as the assessments of the other readout parameters in each study. If clinical score data were not considered for our analysis, this is related to the absence of a separate reporting of outcomes for the clinical score‘s activity-specific parameters (Tables 2, 3 and 4). Clinical Scores were assessed during the light phase of the animals’ circadian rhythm.

**Table 2:** Changes in activity-related readout parameters in surgical interventions in rodents.

Study ID	Surgical procedure	Reference data	Clinical Score	Neuroscore	Open field	VWR (light-off)	Telemetry		Home cage-based video monitoring	
							Light-on	Light-off	Light-on	Light-off
Post-surgical day 0										
Intraperitoneal Transmitter Implantation		Baseline	~					↓		
Pancreatic Cancer Model	Laparotomy + cell injection	Baseline	↓					↓		
Cerebellar Cognitive Affective Syndrome Model	Intracerebral tissue ablation	Baseline	↓		↓					
		Naïve	↓		↓					
		Sham	↓		↓					
Post-surgical day 1										
Intracerebral Electrode Implantation		Baseline	↓ / ~	↓ / ~						
		Naïve	↓ / ~	↓ / ~		↓ / ~			↓ / ~	↓ / ~
		Sham (drug-control)	↓ / ~	↓ / ~		↓ / ~			↓ / ~	↓ / ~
Subarachnoid Hemorrhage Model	Intracerebral vessel perforation	Baseline	↓	↓	↓					
		Sham	~	~	~					
		Sham (drug-control)	↓		~					
Partial Hepatectomy		Baseline	↓ / ~		↓ / ~			↓		
		Sham	~		~					
Epilepsy Model – Post SE (IHK)	Intracerebral injection and implantation	Sham	↓				~	~		
		Sham (transmitter)	↓				~	↓		
Parkinsonism Model	Intracerebral injection	Baseline	↓		↓					
		Sham	↓		~					
Glioma Model	Intracerebral cell injection	Baseline	~		~					

↑ = increased activity; ↓ = decreased activity; ~ = no changes. Changes were interpreted based on the effect size calculation. Changes in different readout parameters within the same study were recorded on the same post-surgical day. More than one legend indicates more than one outcome among the experimental/intervention groups in respective studies. VWR, Voluntary wheel running; SE, Status epilepticus; IHK, intrahippocampal kainate.

**Table 3:** Changes in activity-related readout parameters in genetic disease models and in neuroscientific and stress tests/techniques used in rodents.

Study ID	Reference data	Clinical Score	Neuroscore	Open field	Voluntary wheel running	Telemetry (Light-off)	Home cage-based video monitoring
Genetic disease models							
Dravet Syndrome Model	Wildtype	~	↑ / ~	↑			↑ / ↓ / ~
*Gria1* KO Model	Wildtype	~	↑ / ~	↑	↓ / ~		↑ / ~
DAT KO Model	Wildtype	~	↑	↑			
Neuroscientific and stress tests/techniques							
Restraint Stress Model	Baseline				↓		
	Naïve				↓		
Forced Swim Test	Baseline			↓			
	Naïve			~			
	Sham			~			
Biopotential Recording Techniques	Baseline	~				~	

↑ = increased activity; ↓ = decreased activity; ~ = no changes. Changes were interpreted based on the effect size calculation. Changes in different readout parameters in the same study were recorded on the same day or experimental phase. More than one legend indicates multiple outcomes among the experimental/intervention groups in the respective studies.

**Table 4:** Changes in activity-related readout parameters in non-genetic rodent disease models.

Model ID	Reference data	Clinical Score	Neuroscore	Open field	VWR	Tele (Light-on)	Tele (Light-off)
Epilepsy Model – Kindling (hippocampus)	Naïve	~	↑	~			
	Sham	~	↑	↑			
Epilepsy Model – Kindling (basolateral amygdala)	Naïve	~	↑	~			
	Sham	~	↑	↑			
Epilepsy Model – Post SE (intrahippocampal kainate)	Naïve	~	↑	~			
	Sham	~	↑	~		~	~
	Sham (transmitter)	~	↑	~		~	~
Colitis Model (Lab 1)	Baseline				↓		
	Sham				↓		
Colitis Model (Lab 2)	Baseline	↓			↓		
Pancreatic Cancer Model	Baseline	~					~
Epilepsy Model – Kindling (tethered)	Naïve			~			
	Sham			~			
Epilepsy Model – Kindling (telemetry)	Baseline	~					~
	Sham	~				↑ / ~	~
	Sham (transmitter)	~				↑ / ~	~
Epilepsy Model – Post SE (pilocarpine)	Baseline	~					~
	Naïve	~		~			
	Sham	~	~	~		~	~
Epilepsy Model – Post SE (electrical)	Baseline	~					~
	Naïve	~		↑ / ~			
	Sham	~	↑ / ~	↑ / ~		~	~
Hearing Loss Model	Baseline	~		↑			
	Naïve	~		↑ / ~			
	Sham	~		↑ / ~			
Parkinsonism Model	Baseline	↓		↓			
	Sham	~		↓			
Glioma Model	Baseline	~		~			
Glioma Model (endpoint)	Baseline	↓		↓			↓
Cerebellar Cognitive Affective Syndrome Model	Baseline	~		~			
	Naïve	~		~			
	Sham	~		~			

↑ = increased activity; ↓ = decreased activity; ~ = no changes. Changes were interpreted based on the effect size calculation. Changes in different readout parameters in the same study were recorded on the same day or experimental phase. More than one legend indicates multiple outcomes among the experimental/intervention groups in the respective studies. VWR, Voluntary wheel running; Tele, Telemetry; SE, Status epilepticus.

Activity-related Clinical Scores were categorized as “yes” (if activity changes were observed) or “no” (if no activity changes were observed) to ensure consistent comparisons across the models. Most scoring systems were designed to detect a reduction in activity. For those considering the directionality of the activity-related changes, this was noted in the central file and taken into account during the analysis.

#### Neuroscore

The Neuroscore comprises a selection of parameters from a test battery developed initially by Samuel Irwin^29^; specifically, these parameters were chosen to provide valuable information for assessing the animal´s severity while avoiding pain-inducing techniques^11,13,30^. For the current analysis, we extracted data exclusively from the “locomotor activity” parameter. This parameter considers “0” as the normal condition, positive scores as increased activity, and negative scores as decreased activity. As with the Clinical Score, these scores were categorized as “yes” (if activity changes were observed) or “no” (if no activity changes were observed) to ensure consistent comparisons across the models. Directionality was considered during the analysis. Neuroscore data were assessed during the light phase of the animals’ circadian rhythm.

#### Open field

The OF test can assess exploratory behavior and locomotor activity. Rodents are placed in an open arena and video-monitored for a specified period. For analysis, we extracted data for “total distance moved”. Notably, the selected parameter encompassed the entire duration of the monitoring, without differentiating between exploratory behavior that can be analyzed during the first minutes after exposure to the new environment. Information regarding specific methodological details from the different studies (e.g., duration of video monitoring or type of arena) can be found in Supplementary Table S3. The OF test was performed during the light phase of the animals’ circadian rhythm, except for the Colitis Model (Lab 2) study.

#### Voluntary wheel running

Assessment of VWR is a non-invasive, home cage-based method for evaluating an animal’s activity. Running wheels are placed in the home cage of the animals to track their activity for a specified period of time. For analysis, we extracted data for the “total distance run” parameter. Information regarding specific methodological details from the different studies (e.g., type or position of the running wheel) can be found in Supplementary Table S4. For the majority of the studies, VWR data were reported as a single value, considering both the light and dark phases of the animals’ circadian rhythm, or even the entire exposure time.

#### Telemetry

Telemetry allows continuous monitoring of activity and other physiological parameters in freely moving animals. In our analysis, we considered data from studies that used wireless transmitters (surgically implanted subcutaneously or intraperitoneally) for recording in their home cages. The activity measured as “counts per minute (cpm)” was selected for further analysis. Methodological details of telemetric data acquisition are provided in Supplementary Table S1. Notably, most of the telemetric data originated from recordings during the dark phase, i.e., the active phase of the animals’ circadian rhythm.

#### Home-cage-based video monitoring

Home-cage-based video monitoring enables a continuous analysis of activity and other physiological parameters without the need for surgical implantation of any device. In the studies considered for our analysis, animals were housed in modified home cages equipped with infrared video cameras (PhenoTyper, Noldus, Wageningen, Netherlands). “Total distance moved” was selected as the parameter of interest for this analysis. Methodological details of data from home-cage-based video monitoring can be found in Supplementary Table S5. Of the three included studies, two reported activity data as a ratio of distance per hour.

### Project-specific comparisons of interest

This step aimed to identify potential comparisons between experimental/intervention groups and reference data for each study. Effect sizes and 95% confidence intervals (95% CI) were calculated by comparing data from the “experimental/intervention” group(s) with the corresponding reference data. A change in the activity-related readout parameter was considered only when the 95% CI of the effect size did not cross zero, independently of the actual value of the effect size. Note that multiple effect sizes for a single study were possible when the study included different reference categories (e.g., experimental animals vs. naïve and experimental animals vs. sham).

When activity readout parameters were assessed at multiple time points, data from the earliest assessment after surgery (day 0 or day 1, see Table 2) were selected for studies in the surgical intervention category. For studies from other categories, or for surgical intervention studies that included measurements from a later disease phase, only data from a single assessment time point were considered for analyses. Specifically, data from the time point with the largest and statistically significant effect size were selected for the meta-analysis and ROC analysis and included in tables summarizing changes in activity. If none of the effect sizes across later disease phase time points reached statistical significance, data from the assessment day furthest from surgery or follow-up were selected for comparison.

### Meta-analysis decision

To summarize the findings from different models or interventions, we aimed to perform a meta-analysis. This analysis was conducted only when more than five comparisons were available in a methodological category (e.g., surgical intervention), all using the same category of reference data (e.g., baseline data). Otherwise, only the effect size and 95% CI were calculated.

### Receiver operating characteristics and cross-correlation analysis

To compare the performance of the evaluated activity-related readout parameters within the same study, we performed a Receiver Operating Characteristic (ROC) analysis. For this analysis, we included only some exemplary studies (considering the number of parameters evaluated and the classification of the study). The included studies focused on partial hepatectomy, intracerebral electrode implantation, post-status epilepticus (SE) epilepsy models, and genetic neurological disease models. As an exception, and to fully explore the assessment of the readout parameters, we combined data from two studies investigating rat post-status epilepticus (SE) models of temporal lobe epilepsy (Epilepsy Model – Post SE pilocarpine and electrical) and two studies involving genetic mouse models (Dravet Syndrome Model and *Gria1* KO Model, Table 1). The combined data shared harmonized methodological protocols for data collection on the included readout parameters.

Data from telemetric or home cage-based video monitoring served as our “gold standard” and were therefore used to estimate a classification threshold. We presumed that these two techniques are closest to measuring the actual spontaneous activity of the animals in their home cage without experimental manipulation.

Threshold selection was based on one of the reference data categories per study (baseline, sham, or naïve). Data from the selected reference were analyzed for normality using QQ plots, histograms, and the Shapiro-Wilk test. If a normal distribution was found, then the threshold values were determined by mean ± standard deviation; otherwise, the quartiles 1 and 3 were used (Supplementary Figure S2). Once defined, these thresholds were applied to the complete dataset (including experimental/intervention groups, post-intervention days, or experimental phases) to classify each observation into increased or decreased activity.

ROC analysis evaluated the performance of various activity-related readout parameters considering the classification by the selected “gold standard” as a basis for calculation. For studies where a particular variable appears to significantly impact the interpretation of the results (e.g., sex, developmental stage, etc.), the analysis was performed independently for each subset of data.

In parallel, a cross-correlation analysis was performed among all the readout parameters evaluated in each study. This analysis included the complete dataset, independently of experimental/intervention groups, reference data, or multiple collection days.

### Statistics

Microsoft^®^ Excel^®^ (Microsoft 365 MSO, Version 2503 Build 16.0.18623.20266, 64-bit) was used for preprocessing and storing the data and calculating the effect sizes and 95% CI of the telemetric data. R (version 4.5.0) in RStudio (2025.05.0 Build 496, Posit Software) was used to calculate the effect sizes and 95% CI of the remaining readout parameters and for the meta-analysis, ROC analysis, and cross-correlation of the selected studies. A table with the specific packages used and their explanation can be found in Supplementary Table S6. GraphPad Prism^®^ 10 (GraphPad Software, version 10.4.1 (627), 64-bit) was used to illustrate the results graphically.

Cohen’s H effect size and 95% CI (based on t-distribution) were calculated for Clinical Scores and Neuroscores^31,32^. For the remaining readout parameters, Cohen’s D effect size with a Hedges’ G correction and 95% CI were calculated^33^.

For dichotomic outcomes (Clinical Score and Neuroscore), odds ratios (ORs) were calculated for individual studies and pooled using the common (Mantel-Haenszel method) and random (Inverse variance method) effects models. Additionally, we applied continuity correction of 0.5 in studies with zero cell frequencies when applicable. For quantitative outcomes (OF), standardized mean differences (SMD) were calculated for individual studies and pooled using the common and random (Inverse variance method) effects models. Additionally, we applied Hedge’s G correction to account for possible small sample sizes for some studies. Heterogeneity among the studies was evaluated with the restricted maximum-likelihood estimator for tau^2^ and I^2^ (based on Q).

The data distribution for selecting a threshold was assessed using the Shapiro-Wilk test and QQ-plots. For the ROC analysis, area under the curve (AUC) values were calculated using the trapezoidal rule, and their 95% CI were calculated using the DeLong method. A Spearman correlation test was used to estimate the correlation coefficients and p-values for the cross-correlation analysis.

### Animal and experimental information

Most of the studies included in our comprehensive cross-model and cross-intervention analysis have already been published (relevant references can be found in Supplementary Table S7). Approval was granted by the corresponding author for the use of the data in our analysis. According to the authors of the original publications or the investigators of the respective studies, all included experiments were conducted in accordance with German legislation and EU Directive 2010/63/EU and were approved by the local authorities. Corresponding reference numbers of the animal allowances and responsible authorities are listed in Supplementary Table S7. The original reporting was in accordance with the respective ARRIVE guidelines^34,35^(version 1.0 or 2.0 depending on the year of publication). Supplementary Table S7 provides information regarding the risk of bias of the projects included.

General information about the animals used in the original studies, and their housing and husbandry can be found in Supplementary Table S8. Information on analgesia and anesthesia protocols for studies in the surgical interventions category is available in Supplementary Table S9.

### Information about studies focused on different animal models and interventions

#### Kindling model of temporal lobe epilepsy in mice (Epilepsy Model – Kindling hippocampus and basolateral amygdala)^36^

Kindling refers to a chronic animal model of focal to bilateral tonic-clonic seizures similar to those observed in patients with temporal lobe epilepsy. This model involves the repeated electrical induction of seizures, which leads to a progressive increase in seizure severity and a persistent reduction in seizure thresholds^37–39^. In later phases, animals display seizures with focal onset that secondarily progress to generalized seizures. In this study, an intracranial electrode in the basolateral amygdala or hippocampus was surgically implanted in female HsdWin: NMRI mice. After a recovery period (approximately 14 days), seizure induction was initiated by repeated electrical stimulation (kindling). Clinical Score, Neuroscore, and OF data used for analysis were collected in fully kindled mice (2–5 weeks after kindling initiation). The reference data for comparison included data from a naïve group and a sham group.

#### Amygdala kindling model of temporal lobe epilepsy in rats (Epilepsy Model – Kindling tethered and telemetry)^16,40^

This model shares the pathophysiological principles and face validity of its equivalent model in mice. In this study, a subcutaneous telemetric transmitter and an intracranial electrode were surgically implanted in the basolateral amygdala of female Sprague-Dawley rats. After a recovery period (approximately 17 days), seizure induction was initiated by repeated electrical stimulations (kindling). Telemetric data were recorded at baseline (days three and two prior to the start of kindling) and during two phases of the model (during focal seizures and during generalized seizures). Additionally, Clinical Score and OF data were used for the analysis. Notably, this study employed two slightly different experimental setups to collect the OF and telemetric data, each setup involving distinct animals (tethered and telemetry sub-studies). The reference data for comparison included baseline data, data from a transmitter-only sham (sham tm) group, and data from a transmitter + electrode sham (referred to as “sham”) group.

#### Intrahippocampal kainate model of temporal lobe epilepsy in mice (Epilepsy Model – Post SE intrahippocampal kainate)^13^

The intrahippocampal kainate model in mice (IHK) is a chronic post-SE model widely used to study temporal lobe epilepsy. The model is characterized by three phases: (1) an acute phase marked by the induction of SE and associated brain injury, (2) a latency phase during which the animals do not display any seizures, but the brain is subject to various biological processes that contribute to the development of a hyperexcitable neural network, and (3) a chronic phase characterized by the presence of spontaneous recurrent seizures^41,42^. For monitoring purposes, a subcutaneous telemetric transmitter and an intracerebral electrode were surgically implanted in female HsdWin: NMRI mice. During the same surgery, the animals received an injection of kainate into the right CA1 region of the dorsal hippocampus to induce SE. The telemetric recordings took place during the first 96 h after SE induction, as well as during the chronic phase of the model (for 48 h at two weeks and four weeks after SE induction). Clinical Score, Neuroscore, and OF data collected post-surgically and/or during the chronic phase (i.e., 4–8 weeks after SE induction) were used for the analysis. The reference data for comparison included data from transmitter-only sham (sham tm) and transmitter + electrode sham (referred to as “sham”) groups.

#### Pilocarpine induced post-status epilepticus model of epilepsy in rats (Epilepsy Model – Post SE pilocarpine)^14,43^

This model is used to study temporal lobe epilepsy and can be characterized by the same three phases as the IHK model in mice^44,45^. In contrast to the IHK, this model is based on the systemic administration of pilocarpine for SE induction. For monitoring purposes, an intrahippocampal electrode (right dentate gyrus) and a subcutaneous telemetric transmitter were surgically implanted in female Sprague-Dawley rats. After a recovery period (approximately 18 days), pilocarpine was injected (in lithium pre-treated animals) to induce SE. Telemetric data were recorded on days three and two before SE induction (baseline) and on selected days during the first (early post-insult phase), fourth (latency phase), and ninth (chronic phase) week after SE induction. Additionally, Clinical Score, Neuroscore, and OF data were used for analysis. Of note, the studies employed three slightly different experimental setups, each involving distinct animals (tethered, telemetry, and positron-emission tomography sub-studies). The reference data for comparison included baseline data and data from a sham group.

#### Electrically induced post-status epilepticus model of epilepsy in rats (epilepsy model – post SE electrical)^15,46^

This model resembles features of temporal lobe epilepsy and reproduces the same three phases as the other two post-SE models described above^47^. In contrast to the pharmacologic induction of SE used in other post-SE models, SE is induced here by electrical stimulation of the basolateral amygdala. For stimulation and monitoring purposes, an electrode (in the basolateral amygdala) and a subcutaneous telemetric transmitter were surgically implanted in female Sprague-Dawley rats. After a recovery period (approximately six weeks), SE was induced by electrical stimulation via the implanted electrode. Telemetric data were recorded on the two days before SE induction (baseline) and on specific days during the first (early post-insult phase), fourth (latency phase), and ninth (chronic phase) week after SE induction. Additionally, Clinical Score, Neuroscore, and OF data were used for analysis. The reference data for comparison included baseline data and data from a sham group.

#### Model of hearing loss in rats (hearing loss model)^48–50^

This model involves the chemical induction of deafness by injecting the ototoxic compound neomycin directly into the cochlear of male Sprague-Dawley rats. Data from this model were obtained from two groups: neomycin injected in juvenile (P14^49^) and adult (> P70^48,50^ – two cohorts) rats. Animals were followed for up to 24 weeks in the juvenile and 1st adult cohorts (1st subgroup) and up to 56 weeks in the 2nd adult cohort. Clinical Score and OF data collected during the follow-up were used for analysis. The reference data for comparison included baseline data (2nd adult cohort) and data from naïve (adult, both cohorts) and sham (all cohorts) groups.

#### Colitis mouse model (Colitis Model laboratory 1)^8^

Colitis was induced by the repetitive administration of Dextran Sodium Sulfate (DSS). Thirteen-week-old female C57BL/6J mice were treated with DSS (0%, 1%, or 1.5%) from days one to five and followed up until day 14. Additionally, blood sampling was performed in one cohort of animals on days zero, five, and 14. VWR data were collected daily for 20 h, starting 14 days before the experiments (adaptation period) and continuing throughout the follow-up period. These VWR data were used for analysis. The reference data for this model were baseline data.

#### Colitis mouse model (Colitis Model laboratory 2)^9^

In this study, colitis was induced following a similar methodology to the one described above (Colitis Model laboratory 1). Eight-week-old male C57BL/6 N mice received 1% DSS from days one to five and were followed up until day 16. VWR data were collected daily during the dark phase, starting 16 days before the experiment (adaptation period) and continuing throughout the follow-up. These VWR data were used for analysis. Baseline data served as reference data for this model.

#### Model of parkinsonism in rats (Parkinsonism Model)^51^

Adult male Sprague-Dawley rats underwent the stereotactic intracerebral injection of 6-hydroxydopamine (6-OHDA) in the right medial forebrain bundle for induction of nigrostriatal loss of dopaminergic neurons, a hallmark of Parkinson’s disease in humans. Clinical Score and OF data were used for analysis. The reference data for comparison included baseline data and data from a sham group.

#### Intracerebral glioma model in rats (glioma model)^51^

Adult male BDIX rats underwent stereotactic intracerebral injection of BT4Ca glioma cells into the right frontal cortex. This rat model replicates many aspects of the infiltrative and fast growing malignant brain tumor, as described in the human disease. Animals were followed during the development of intracranial glioblastoma until a humane endpoint criterion, defined as sudden onset weight loss of 3–5% combined with deteriorated general condition (mild ataxia/postural instability). Clinical Score and OF data were used for analysis. Baseline data served as reference data for this model.

#### Intracerebral glioma model in rats (glioma model endpoint)^52^

In this study, a subcutaneous telemetric transmitter was surgically implanted in male BDIX rats. After a recovery period (approximately four weeks), BT4Ca cells were injected and rats were monitored by applying humane endpoint criteria as previously described (Glioma Model). The mean survival time of the transmitter-implanted animals was 14 days (range: 13 to 16 days) after tumor cell injection. Telemetric data were recorded three days before cell implantation (baseline) and during the last eight days before reaching the humane endpoint (day 0). Additionally, Clinical Score and OF data were used for analysis. The reference data for this model were baseline data.

#### Model of pancreatic cancer in mice (Pancreatic Cancer Model)^20^

A telemetric transmitter was surgically implanted intraperitoneally in male C57BL/6J mice. After a recovery period (approximately 14 days), mice were orthotopically injected with pancreatic carcinoma cells during laparotomy. Animals received an analgesic drug (metamizole/dipyrone) via drinking water throughout the entire experiment (from transmitter implantation until euthanasia, performed 37 days after cell implantation). Telemetric data were recorded one day before tumor cell implantation (baseline), early after cell implantation, and during tumor growth. Clinical Scores assessed on the respective days were used for the analysis. The reference data for this model were baseline data.

#### Subarachnoid hemorrhage via endovascular perforation using a micro-filament in rats (Subarachnoid Hemorrhage Model)^53^

Aneurysmal rupture can cause subarachnoid hemorrhage (SAH), resulting in hemorrhagic stroke. In this study, SAH was induced by perforating the circle of Willis using a microfilament in male Wistar rats. The animals were followed up for seven days. Clinical Score, Neuroscore, and OF data were used for analysis. The reference data for comparison included baseline data and data from sham animals that received the same surgical procedures as SAH without vessel perforation, and anesthesia-only animals that did not receive any surgical procedures (considered as “sham (drug-control)”).

#### Intracranial electrode surgery in mice (Intracerebral Electrode Implantation)^30^

Implantation of intracerebral electrodes is a standard surgical procedure in the field of neuroscience. In this study, female and male C57BL/6J wildtype mice underwent intracerebral electrode implantation. Then, animals received different analgesic regimens (monotherapy of a non-steroidal anti-inflammatory drug compared with three different multimodal regimens combining a non-steroidal anti-inflammatory drug with a local anesthetic and/or an opioid). Clinical Score, Neuroscore, VWR, and home-cage video monitoring data were used for analysis. The reference data for comparison included baseline data and data from naïve and drug control (considered as sham) groups.

#### Thermal lesion of the cerebellar fastigial nucleus as a neurodevelopment model for Cerebellar Cognitive Affective Syndrome^54^

The long-term effects of a lesion of the fastigial nucleus in juvenile rats reproduce the symptoms observed in patients with cerebellar cognitive affective syndrome. In this study, male Sprague-Dawley rats were subjected to the bilateral lesion via thermocoagulation of the fastigial nucleus at P23 and followed for up to 24 weeks. Clinical Score and OF data collected during follow-up were used for analysis. The reference data for comparison included baseline data and data from naïve and sham groups.

#### Intraperitoneal transmitter implantation in mice (Intraperitoneal Transmitter Implantation)^19^

For this study, a telemetric transmitter was intraperitoneally (i.p.) implanted in female C57BL/6J mice. The study consisted of two experimental groups that received different analgesic regimens. In one group, animals received carprofen pre-surgery and for three post-surgical days (pre-surgery: 5 mg/kg subcutaneously; post-surgery: 2.5 mg/kg subcutaneously every twelve hours). In the second experimental group, mice received metamizole (dipyrone) pre-surgery and until post-surgical day three (pre-surgery: 200 mg/kg subcutaneously; post-surgery: 200 mg/kg orally via drinking water). Animals from both groups were monitored for 28 days. Telemetric data were recorded during the first two post-surgical weeks and on day 28 (baseline). Clinical Scores assessed on the respective days were used for the analysis. The reference data for this model were baseline data.

#### Partial hepatectomy in rats (Partial Hepatectomy)^18,55,56^

Partial hepatectomy is a widely used method for studying the regenerative properties of the liver. In this study, male Wistar-Han rats underwent partial hepatectomy (50% and 70%). Subcutaneous telemetric transmitters were surgically implanted in a sample of these animals approximately 15 days before the partial hepatectomy. Telemetric data were recorded two days before (baseline) and during the first week after partial hepatectomy. Clinical Score and OF data collected during follow-up were used for analysis. The reference data for comparison included baseline data and data from a sham group.

#### Genetic knockout of the dopamine transporter in rats (DAT KO Model)^57^

By knocking out the dopamine transporter (DAT), characteristic features for neuropsychiatric diseases associated with hyperdopaminergia, and symptoms observed in patients with Parkinson´s disease are mimicked. DAT KO, DAT^+/−^, and DAT^+/+^ rats were generated on a Wistar-Han background. Only male animals were used for the experiments. Clinical Score, Neuroscore, and OF data were used for analysis. The reference data for comparison included data from the naïve group (i.e., wildtype).

#### Mouse model of Dravet syndrome (Dravet Syndrome Model)^11^

Dravet syndrome is a developmental epileptic encephalopathy frequently caused by a genetic variant in the *SCN1A* gene, resulting in difficult-to-treat seizures, behavioral alterations, and cognitive and motor dysfunction. In this study, experimental animals carrying a heterogeneous A1783V mutation in the *Scn1a* gene (described in human patients with Dravet syndrome) on a mixed background (C57BL/6J and 129S1) were used. Clinical Score, Neuroscore, OF, and home-cage video monitoring data were used for analysis. The reference data for comparison included data from the naïve group (i.e., *Scn1a*^+/+^).

#### Mouse model of neuropsychiatric disorders (Gria1 KO Model)^12^

This model mimics features associated with glutamatergic dysfunction, which is seen in many neurodevelopmental and psychiatric disorders. In this study, *Gria1* KO mice were considered experimental animals. Animals were bred on a C57BL/6 N background. Clinical Score, Neuroscore, VWR, OF, and home cage video monitoring data were used for analysis. The reference data for comparison included data from the naïve group (i.e., wildtype).

#### Restraint stress mouse model (Restraint Stress Model)^8^

This model is based on inducing chronic stress in mice by restraining their mobility. Thirteen-week-old female C57BL/6J mice underwent 60 min of restraint stress daily for ten days. Restraint consisted of limiting the mice’s ability to move by placing them in a plastic tube with closed ends. Fecal sampling was performed on days zero, seven, and ten. VWR data were collected daily for 20 h, starting 14 days before the experiments (adaptation period) and continuing throughout the follow-up period. These VWR data were used for analysis. The reference data for this model were baseline data.

#### Forced swim test in rats (Forced Swim Test)^58^

The forced swim test (FST) is commonly used in rodents to study behavioral changes following exposure to an inescapable stressor^59^. In this study, female and male Wistar-Han rats underwent FST consisting of two sessions on two consecutive days. During the first day, animals swam for 15 min, and on the second day, for only five minutes. OF data collected during a pre-exposure (i.e., 3 days before FST) and post-exposure (i.e., 3 days after FST) periods were used for analysis. The reference data for comparison included baseline data and data from a naïve and a sham (i.e., vehicle-control) group. Additionally, this study included the use of a drug-control receiving the tricyclic antidepressant Imipramine.

#### Impact of tethered and non-tethered recording techniques in rats (Biopotential Recording Techniques)^21^

This study aimed to evaluate the impact of a tethered compared to a telemetric recording technique on activity and sleep patterns, and thereby on the overall well-being of rats. In contrast to telemetric recording, tethered recording techniques may affect resting and sleeping behavior due to the cable connection between implanted electrodes and the external recording device. In this experiment, a subcutaneous telemetric transmitter and cranial surface screw electrode were implanted in female Sprague-Dawley rats. After a recovery period (approximately four weeks), animals were kept in individual modified plexiglass cages to perform electroencephalographic, electromyographic, and activity recordings during a two-week experimental phase. During the recordings, animals from the tethered group were additionally connected to a cable with a swivel system, allowing them to move freely in the cage. As the implanted transmitters in all animals recorded activity and sleep pattern data, the cables served solely to simulate a tethered recording setup. Telemetric data from the tethered and non-tethered animals were recorded on two days before the experimental acquisition phase (baseline) and during the experimental phase in plexiglass cages. Clinical Scores assessed on the respective days were considered for analysis. The reference data for this model were baseline data. Notably, baseline data were recorded in a different environment compared to the experimental recordings, i.e., before the animals were moved to the monitoring cages (animals’ previous home cage vs. glass cage).

## Results

### Studies and readout parameters overview

A total of 48 studies evaluating any of the selected readout parameters were identified; however, only 23 (48%) met our inclusion criteria. The inclusion rate per parameter ranged from 38% (9/24) for studies analyzing activity using telemetric data to 100% (3/3) for those analyzing it via home-cage-based video monitoring. Details on the inclusion and exclusion of studies are provided in Supplementary Table S10.

### Activity as an informative readout parameter across different study categories

Details on the effect size calculation for each study and the respective evaluated techniques can be found in the Supplementary tables S11-S16 and Supplementary Figures S3-S34. In addition, a summary of all pooled effect sizes and 95% confidence intervals (random-effects model) for the different parameters, interventions/disease models, and reference groups is shown in Fig. 1.

**Fig. 1:**
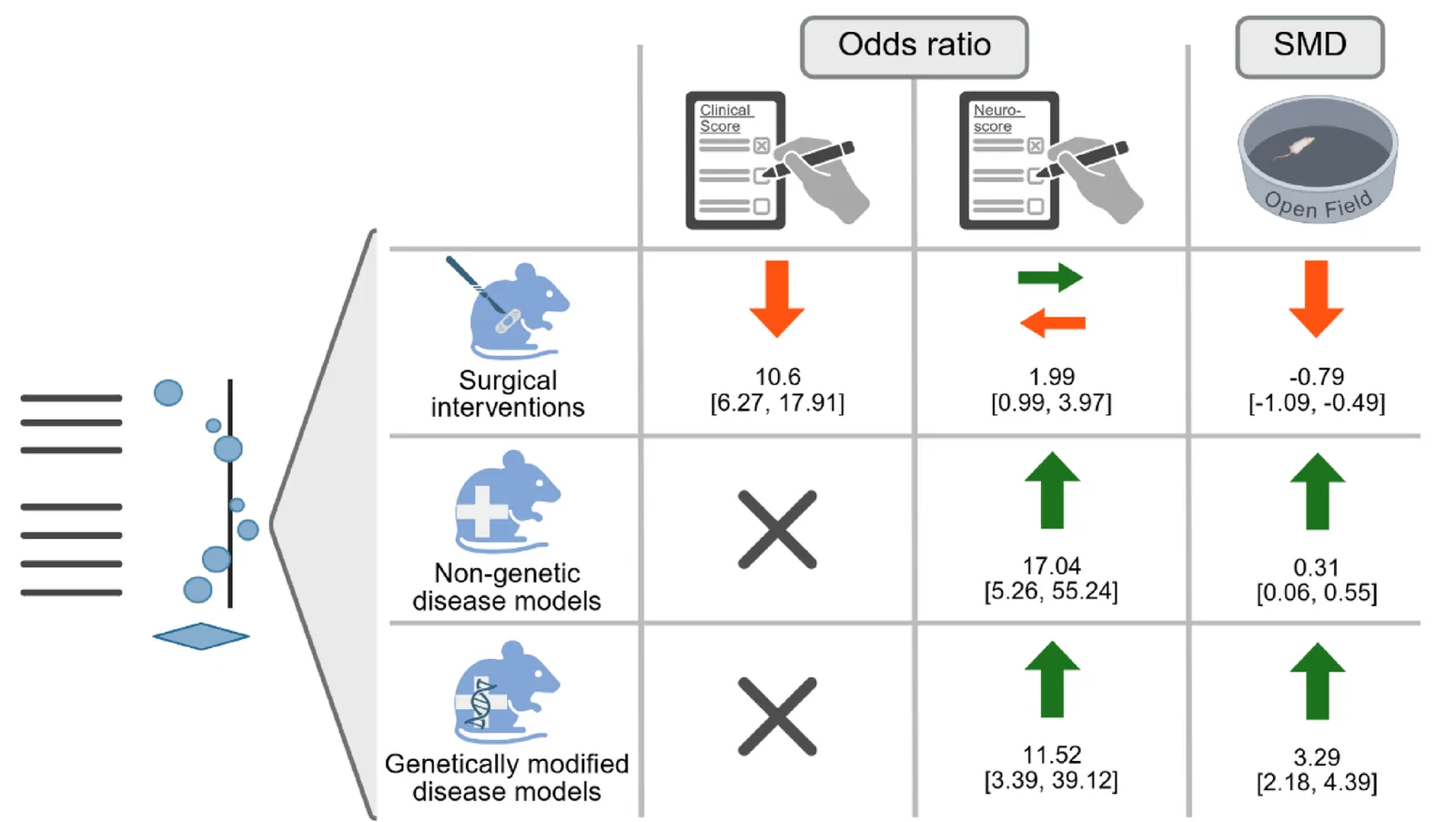
Summary of the high-level pooled effect size for activity-related parameters across different experimental categories. The direction of the arrow indicates the direction of the change in activity. The arrows were drawn using the pooled effect size as calculated by the random effects model, based on Odds ratio (for Clinical Score and Neuroscore) or Standardized Mean Difference (SMD, Open field). The scheme shows the results across pre-defined categories, including surgical interventions, non-genetic disease models, and genetic disease models. Created in BioRender. Potschka, H. (2026) https://BioRender.com/getagpr.

#### Surgical interventions

Following surgical intervention, a decrease in activity (as defined by the effect size and 95% CI) was detected in all studies by at least one of the evaluated techniques, with the Glioma Model in rats representing the only exception (Table 2, Supplementary Tables S11-S12, and Supplementary Figures S3-S11). We identified three patterns regarding the outcomes produced by different activity-related readout parameters for each study evaluating surgical interventions: (1) Changes in activity in all evaluated techniques; (2) No changes in activity in all evaluated techniques; and (3) Inconsistent outcomes across all the evaluated techniques. Notably, the Pancreatic Cancer, Cerebellar Cognitive Affective Syndrome, Subarachnoid Hemorrhage, and Parkinsonism Models were the only ones where a change in activity was detected by all techniques (Table 2, when compared with baseline values).

Effect size analysis revealed that consistency across readout parameters in identifying activity changes within one study depended on the reference data used for comparison. Consistency was highest when baseline values or data from naïve groups, as the best possible control groups, were used as references; however, it substantially declined when sham groups served as the reference, confirming that the sham procedures alone can already exert relevant effects on activity patterns (Table 2).

**Fig. 2:**
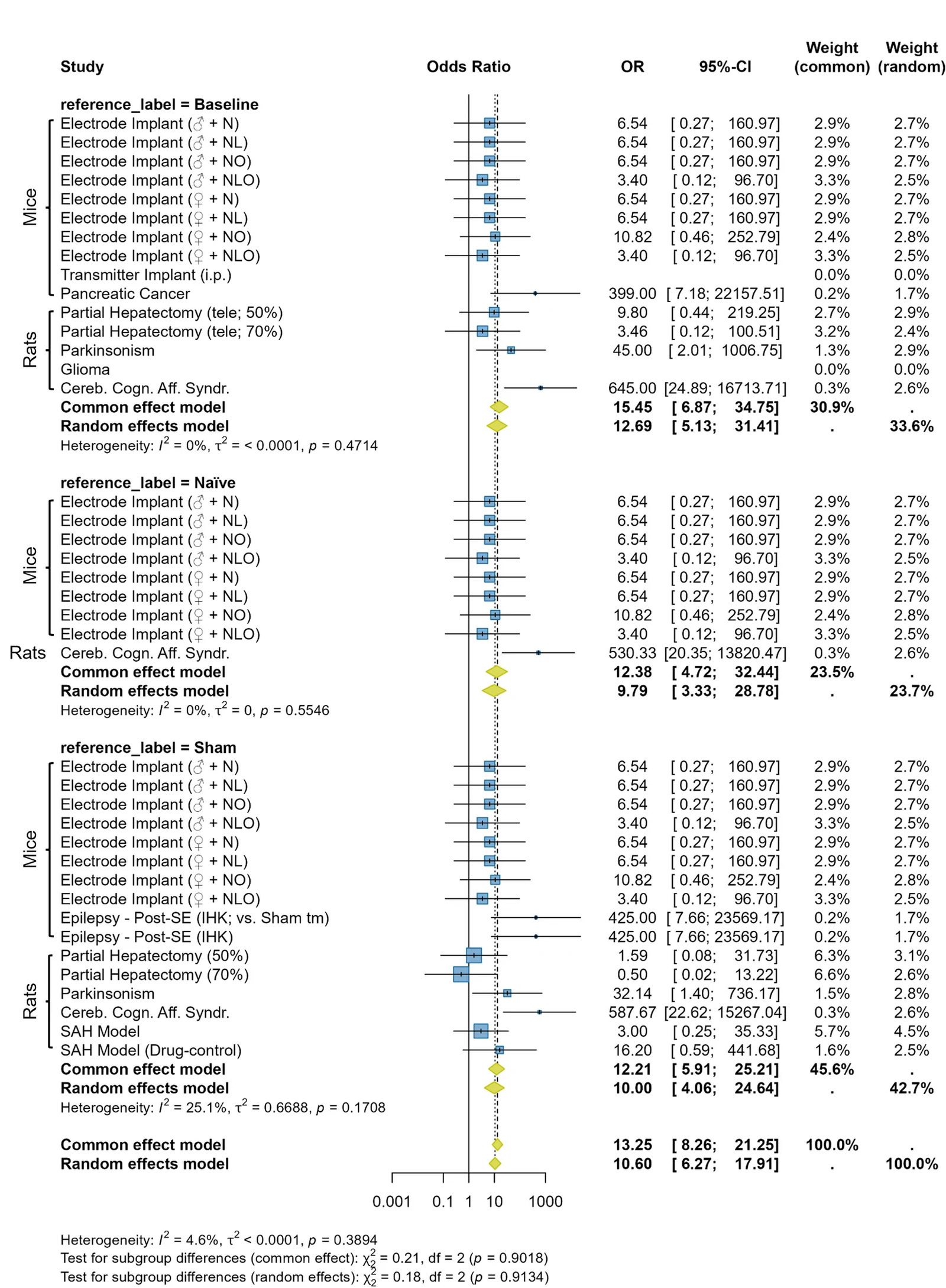
Forest plot depicting a meta-analysis based on Odds Ratios (OR) for changes in selected activity-related parameters of the Clinical Score in mice and rats after different surgical interventions. Data from the earliest evaluation after the surgery (i.e., day 0 or 1) were included in the analysis. Subgroup meta-analysis was performed based on the available reference data. Legends in brackets indicate the specific “intervention” group, except for the Epilepsy Model – Post SE IHK, where it indicated the respective sham group used for calculation. Cereb. Cogn. Aff. Syndr., Cerebellar Cognitive Affective Syndrome Model; CI, Confidence Interval; IHK, intrahippocampal kainate; N, Non-steroidal anti-inflammatory drugs; NL, N + Local anesthesia; NO, N + Opioids; NLO, N + Local anesthesia + Opioids; sham tm, transmitter-only sham; tele, telemetric recording.

**Fig. 3:**
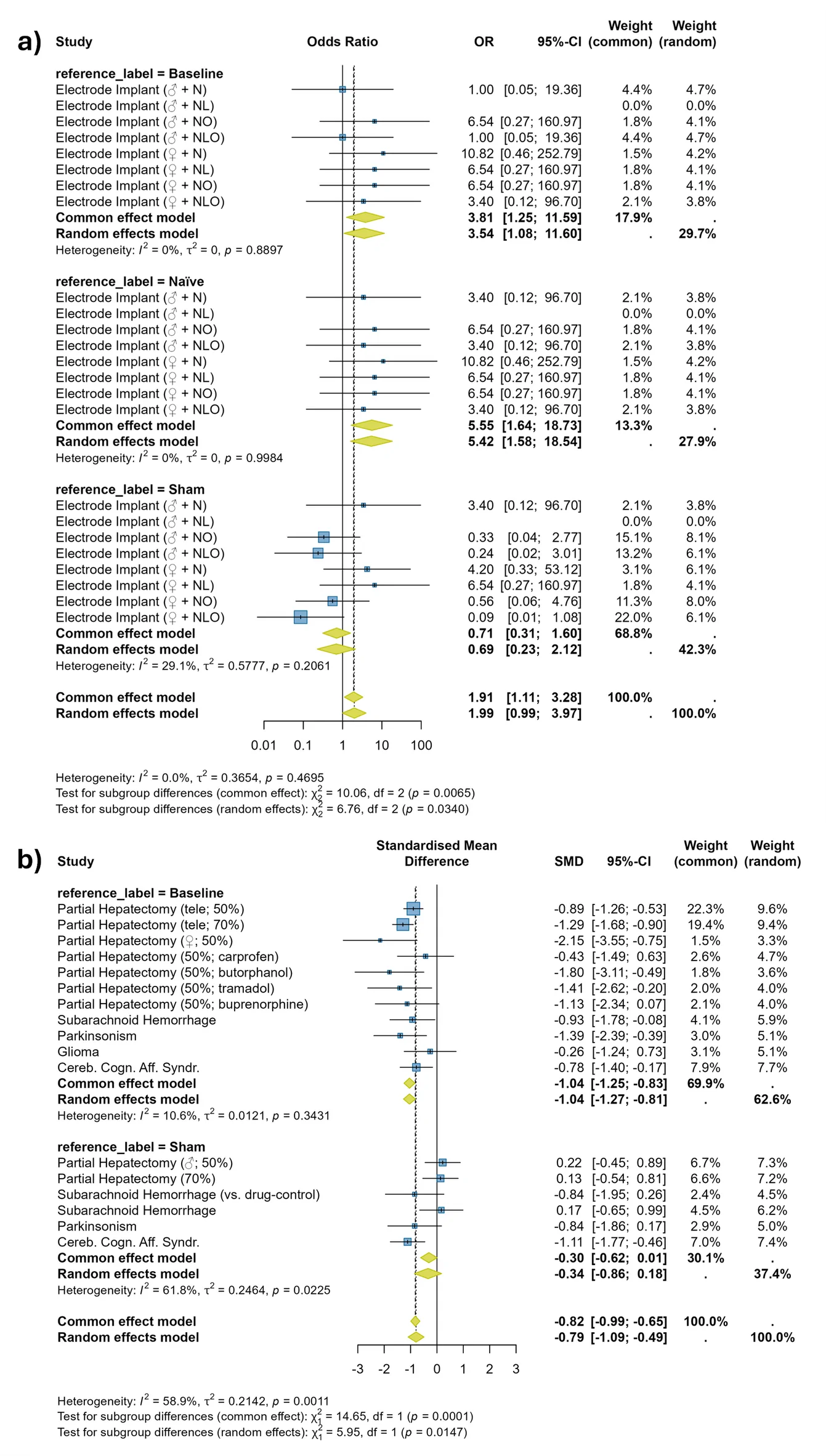
Forest plot depicting a meta-analysis based on Odds Ratios (OR) for changes in the “locomotor” parameter of the Neuroscore in mice (**a**) and the distance moved in the Open field based on Standardized Mean Difference (SMD) in rats (**b**) after different surgical interventions. Data from the earliest evaluation after the surgery (i.e., day 0 or 1) were included in the analysis. Subgroup meta-analysis was performed based on the available reference data. For the Intracerebral Electrode Implantation study, “sham” refers to the respective drug-control groups. Legends in brackets indicate the specific “intervention” group, except for the Subarachnoid Hemorrhage Model, where it indicates the respective sham group used for calculation. Cereb. Cogn. Aff. Syndr., Cerebellar Cognitive Affective Syndrome Model; CI, Confidence Interval; N, Non-steroidal anti-inflammatory drugs; NL, N + Local anesthesia; NO, N + Opioids; NLO, N + Local anesthesia + Opioids; tele, telemetric recording.

The meta-analysis confirmed that animals undergoing surgery often exhibit a decrease in activity during the early post-surgical phase as evaluated by the Clinical Score (Common effect model: 13.25, 95% CI 8.26 to 21.25; Random effects model: 10.60, 95% CI 6.27 to 17.91; *I*^2^ = 4.6%; Fig. 2), the Neuroscore (Common effect model: 1.91, 95% CI 1.11 to 3.28; Random effects model: 1.99, 95% CI 0.99 to 3.97; *I*^2^ = 0%; Fig. 3A), and the OF (Common effect model: -0.82, 95% CI -0.99 to -0.65; Random effects model: -0.79, 95% CI -1.09 to -0.49; *I*^2^ = 58.9%; Fig. 3B).

Notably, the meta-analysis for Neuroscore and OF subgroup analysis confirmed that the outcome of the comparisons depends on the type of reference data. Considering the number of studies analyzing data from the VWR, telemetry, and home cage-based video monitoring, a meta-analysis was not possible. However, these techniques identified a reduction in activity in all studies (Table 2).

#### Neuroscientific and stress tests/techniques

Effect size analysis revealed a change in activity across all studies in this category, except for the study comparing two Biopotential Recording Techniques in rats (Table 3).

The fact that we had access to only one readout parameter for most of the studies included in this category limited our ability to conclude regarding the consistency of the outcomes across techniques (Table 3, Supplementary Tables S13-S14, and Supplementary Figures S12-S14).

#### Non-genetic disease models

A change in activity was detected based on effect size calculations in all studies focusing on non-genetic disease models in at least one of the analyzed readout parameters (Table 4, Supplementary Tables S15-S16, and Supplementary Figures S15-S31). The only exceptions were the Epilepsy Model - Kindling (tethered), the Cerebellar Cognitive Affective Syndrome, and the Glioma Model in rats.

Different techniques for measuring activity in non-genetic disease models yield heterogeneous results, despite being evaluated during the same experimental phase of the modeled disease.

The Colitis (Lab 2), Pancreatic Cancer, Epilepsy Model Post-SE (pilo), Cerebellar Cognitive Affective Syndrome, and Glioma Models exhibited a consistent pattern, either showing a change or no change in activity (Table 4).

**Fig. 4:**
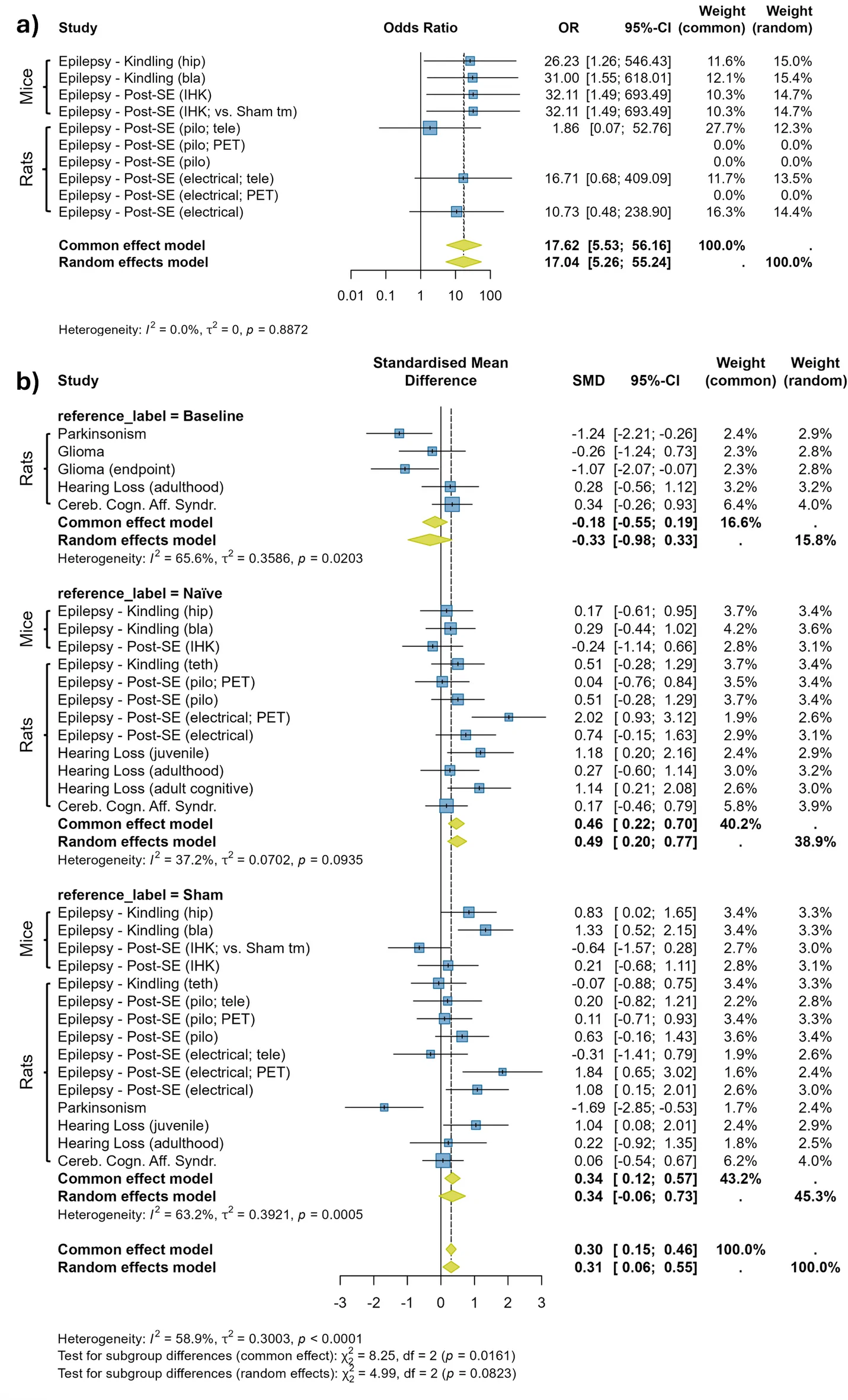
Forest plot depicting a meta-analysis based on Odds Ratios (OR) for changes in the “locomotor” parameter of the Neuroscore (**a**) and the distance moved in the Open field based on Standardized Mean Difference (SMD, **b**) in mice and rats for the non-genetic disease models. Data from a later disease phase of the respective models were selected for analysis. Data from the experimental/intervention group were compared to a sham group(s) in a, while b shows the subgroup analysis considering all reference data available. Legends in brackets indicate the specific “intervention” group, specific experimental cohort (Epilepsy Models – Post-SE and Hearing Loss Model), or specific sham group (Epilepsy Model – Post SE IHK) used for calculation. bla, basolateral amygdala; Cereb. Cogn. Aff. Syndr., Cerebellar Cognitive Affective Syndrome Model; CI, Confidence Interval; hip, hippocampus; IHK, intrahippocampal kainate; pilo, pilorcarpine; SE, Status epilepticus; tele, telemetric recording; teth, tethered recording; sham tm, transmitter-only sham; PET; positron emission tomography.

The meta-analysis results showed an increased risk for any changes in activity across different disease models, as measured by the Neuroscore (Common effect model: 17.62, 95% CI 5.53 to 56.16; Random effects model: 17.04, 95% CI 5.26 to 55.24; *I*^2^ = 0%, Fig. 4A) and the OF (Common effect model: 0.30, 95% CI 0.15 to 0.46; Random effects model: 0.31, 95% CI 0.06 to 0.55; *I*^2^ = 58.9%, Fig. 4B). In contrast to the studies included in the surgical interventions category, these results point towards an increase in activity in the studies considered in these meta-analyses. Subgroup analysis of the OF meta-analysis revealed that the differences were evident regardless of the reference dataset (naïve or sham).

Due to the limited number of studies reporting the technique or a change in the technique, a meta-analysis was not possible for the Clinical Score, VWR, and telemetric data. However, we observed some interesting patterns while comparing the outcomes of different techniques. Except for the Epilepsy Model – Post SE (pilo), all studies evaluating the Clinical Score and the Neuroscore showed inconsistencies regarding changes in activity between these two scoring systems.

Notably, studies evaluating VWR and the same model (Colitis Model) resulted in comparable outcomes regardless of whether the studies were conducted in independent laboratories.

Moreover, it is worth noting that the outcomes of the analysis of telemetric data analysis were inconsistent between the dark and light phases in some of the included studies.

#### Genetically modified disease models

Effect size calculations confirmed changes in activity in all models with genetically modified rodents (Table 3, Supplementary Table S13, and Supplementary Figures S32-S34). However, inconsistent outcomes were observed across activity-related readout parameters for each of the included studies.

**Fig. 5:**
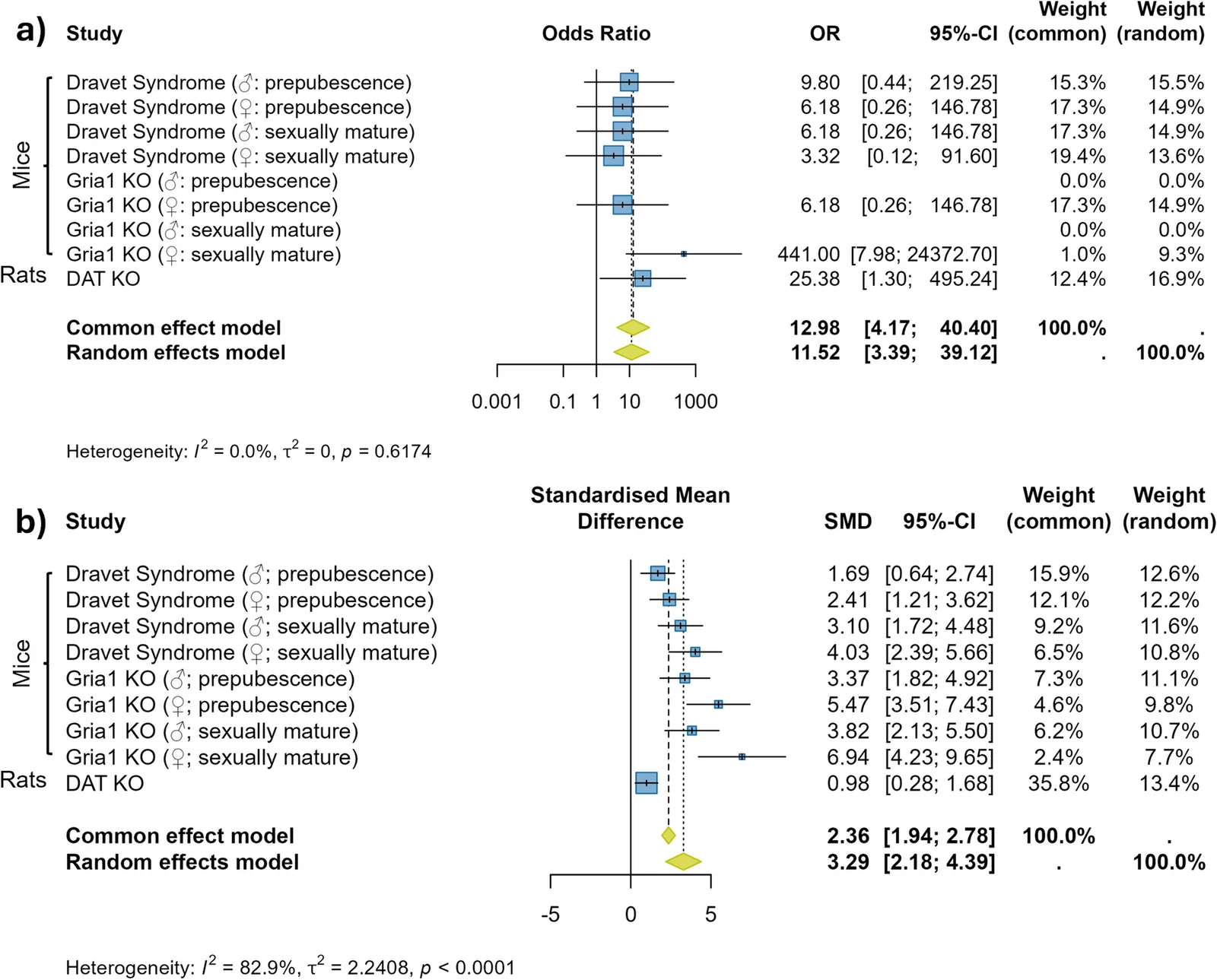
Forest plot depicting a meta-analysis based on Odds Ratios (OR) for changes in the “locomotor” parameter of the Neuroscore (**a**) and the distance moved in the Open field based on Standardized Mean Difference (SMD, **b**) in mice and rats of genetic disease models. The included studies explored the differences vs. wildtype or *Scn1a*^+/+^ animals. Legends in brackets indicate the specific “intervention” group used for calculation. CI, Confidence Interval; DAT, Dopamine transporter; *Gria1*, Glutamate ionotropic receptor AMPA type subunit 1; KO, knockout.

The meta-analysis indicated an increased activity in experimental animals carrying the respective genetic variant, as measured by the Neuroscore (Common effect model: 12.98, 95% CI 4.17 to 40.40; Random effects model: 11.52, 95% CI 3.39 to 39.12; *I*^2^ = 0%, Fig. 5A) or the OF (Common effect model: 2.36, 95% CI 1.94 to 2.78; Random effects model: 3.29, 95% CI 2.18 to 4.39; *I*^2^ = 82.9%, Fig. 5B).

Notably, the Clinical Score outcomes indicated no changes in activity across different studies. The changes observed in the genetically modified animals in home cage-based measurements (VWR and home cage-based video monitoring) were inconsistent across sex or developmental stages of the animals (i.e., *Gria1* KO Model male mice and mature female mice, Table 3 and Supplementary Table S13).

### Performance of different assessment methods to identify activity changes across different models/interventions (ROC and correlation analyses)

For this analysis, we have included only a subset of studies. Moreover, when the experimental setups were harmonized and sufficiently similar, we combined studies that utilized comparable models.

#### Surgical interventions

**Fig. 6:**
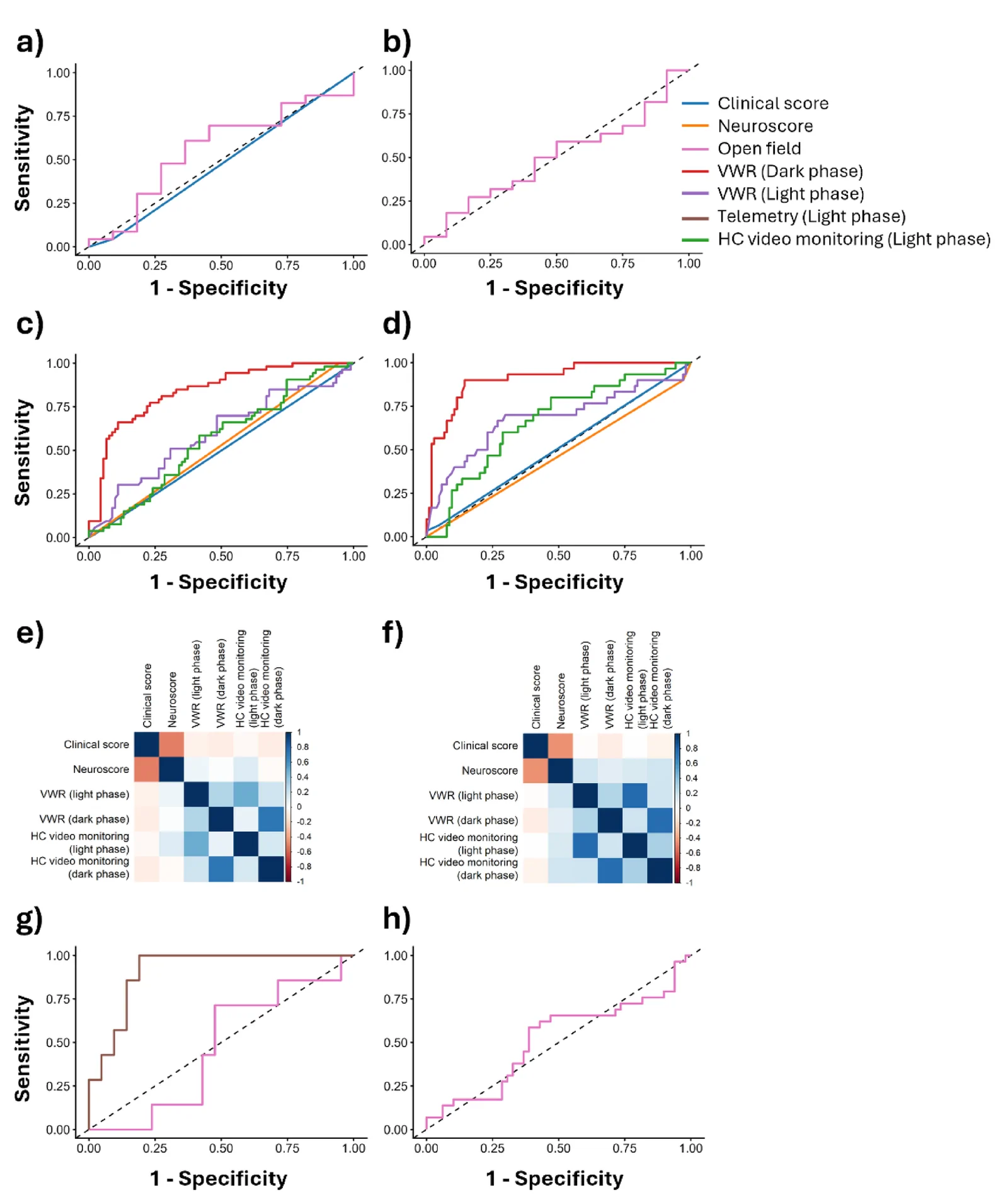
Examples of the Receiver Operating Characteristic (ROC) detecting hypolocomotion and cross-correlation plots for the different experimental categories analyzed. ROC curves for the Partial Hepatectomy intervention (Post-OP day 1: a; Post-OP day 3: b), Intracranial Electrode Implantation surgical intervention (female: c; male: d), Epilepsy Model – Post SE (g), and genetically modified mice (sexually mature, h). Correlation plots illustrating all the parameters evaluated in the Intracranial Electrode Implantation intervention (female: e; male: f). OP, Operation; SE, Status epilepticus; VWR, Voluntary wheel running; HC, Home cage.

In the Partial Hepatectomy study, we observed that the performance of the Clinical Score (AUC: 0.48, 95% CI: 0.38–0.57) and OF (post-surgical day 1: AUC = 0.56, 95% CI: 0.34–0.78; post-surgical day 3: AUC = 0.50, 95% CI: 0.29–0.71) for evaluating a reduction in activity (as defined by the telemetric data) during the post-surgical phase was poor in comparison with telemetry during the active phase (dark phase; Fig. 6, Supplementary Table S17 and Supplementary Figure S35). However, before the surgical intervention, the OF performance was better (AUC = 0.91, 95% CI: 0.76-1.00). Please note that the threshold definition for a relevant change in activity was based on the telemetric data collected during the baseline period.

Moreover, the correlations among data collected with the three techniques for activity analysis (Clinical Score, OF, and Telemetry) were low to negligible, with none reaching statistical significance (Supplementary Table S18 and Supplementary Figure S35).

For the Intracerebral Electrode Implantation study, we considered home cage-based video monitoring data collected from a naïve group as the reference to define the threshold (dark phase, light phase, and complete day, i.e., the sum of activity during the dark and light phases). We observed a reduced detection rate for hypolocomotion when using the light phase (7.6% in females and 6.7% in males) and the complete day activity data (only males: 11.2%) as reference data compared to the detection achieved with our “gold standard” (dark phase: 36.8% in females and 22.4% in males). Consequently, ROC curves were not calculated for these cases. Among the various readout parameters analyzed during the post-surgical phase, the VWR data collected during the dark and light phases performed best in the analyses using the respective continuous video monitoring data as reference, regardless of sex or direction of the change in activity (Supplementary Figure S36 and Supplementary Table S17). Home cage-based video monitoring data collected during the dark phase performed best in the analysis using the complete day continuous video monitoring data as reference (Supplementary Figure S36). The performance of all other readout parameters evaluated was not as good at identifying changes in activity (Fig. 6, Supplementary Figure S37, and Supplementary Table S17).

Correlation analysis revealed a low negative correlation between the two scoring systems (Clinical Score and Neuroscore, Fig. 6 and Supplementary Table S18). Correlations between VWR and home cage-based video monitoring data were mostly low to negligible (all positive), except for the activity recorded during the dark phase in both sexes and during the light phase for males. No further relevant correlations were identified among all other readout parameters.

#### Non-genetic disease models

For this analysis, we have combined two of the Epilepsy Model – Post SE studies (pilocarpine and electrical) because they were conducted with harmonized experimental setups, except for the type of induction used for the initial insult. Threshold values were defined using telemetric data from the baseline period for the dark phase, the light phase, and the complete day (i.e., the sum of the activity during the dark and light phases). The Clinical Score detected no changes in activity across all evaluations in these models and had to be removed from the analysis. Low detection rates (17.9%) for hyper- and hypolocomotion when using data collected during the light phase as the reference data, rather than our “gold standard”, prevented us from calculating the respective ROC curves.

During the chronic phase, telemetric data collected during the light phase performed best (reduced activity: AUC = 0.91, 95% CI: 0.81-1.00, Fig. 6; increased activity: AUC = 0.83, 95% CI: 0.66-1.00) among the evaluated readout parameters, while Neuroscore showed the poorest performance (increased activity: AUC = 0.51, 95% CI: 0.34–0.69, Supplementary Table S17 and Supplementary Figure S38). These results were similar in the analyses using the light and complete day data as references rather than our “gold standard” (Supplementary Figure S39).

The OF data displayed a negligible to low negative correlation with the other readout parameters, including telemetric data from the light and dark phases. A moderate correlation was detected between the telemetric data collected during the dark and light phases (Supplementary Table S18).

#### Genetically modified disease models

For this analysis, we combined data from the Dravet Syndrome and *Gria1* KO Models, and thresholds were defined using home cage-based video monitoring data from the naïve (*Scn1a*+/+ and *Gria1*^*+/+*^) groups. The Clinical Score detected no changes in activity among all the evaluations in these models and had to be removed from the analysis.

The OF data evaluated during the age phase of sexual maturity in mice demonstrated the strongest performance in detecting increased activity (AUC = 0.71, 95% CI: 0.60–0.83, Fig. 6). OF data from the prepubescence age phase and Neuroscore data at both developmental stages displayed inferior performance when evaluating changes in activity (Supplementary Figure S40 and Supplementary Table S17). Most of the correlations ranged from low to negligible among the evaluated readout parameters, with the strongest observed between the Neuroscore and the OF (Supplementary Figure S40 and Supplementary Table S18).

## Discussion

Our consortium’s (DFG FOR2591^24–28^) collection of datasets provided a unique opportunity for assessing activity alterations across a range of experimental interventions and disease models. Studies that implemented different technological approaches enabled a direct comparison of the sensitivity of methods in detecting changes in horizontal activity and locomotion. There is a frequent need for surgical interventions in animal-based research related to the induction of disorders or the implantation of recording devices such as transmitters or electrodes^60–62^. Despite the progress made in pushing multimodal analgesia to minimize the suffering^30,63–65^, there will always be a phase of post-surgical distress related to pain that will likely not be controlled entirely, and to pathophysiological stress and associated discomfort due to the invasive procedure^30,66–69^. Thus, close post-surgical monitoring is essential to control recovery and the success of the analgesic regimen^30,70–72^. Moreover, it is essential to acknowledge that different analgesic compounds, particularly the opioid buprenorphine, may confound behavioral outcomes, including changes in activity patterns^73–76^. Our analysis confirmed a reduction in horizontal activity in the early phase following various surgical procedures, including the implantation of intraperitoneal transmitters or intracerebral electrodes, pancreatic injection of tumor cells, partial hepatectomies, as well as cerebral lesions or cerebral insults induced by thermocoagulation, neurotoxic compounds, or subarachnoid hemorrhages. While these invasive procedures can be associated with different types and intensities of post-surgical pain and distress, analyzing horizontal activity patterns seems to serve as a valuable readout parameter that is consistently affected. The only exception was the Glioma model with stereotactic intracerebral injection of glioma cells^51^, which did not result in a detectable influence on activity as measured by the Clinical Score and OF paradigm. This might be related to a minimally invasive procedure limited to drilling a hole in the skull for the injection without the need for any additional fixation screws. By contrast, more extensive trauma to the skull and surrounding tissue or the administration of a neurotoxic compound could increase the burden and exacerbate the effects on severity grades associated with intracerebral implants. This could be the case in the context of subarachnoid hemorrhage due to the marked increase in intracranial pressure, reduced cerebral blood flow, and inflammation^53^.

In studies employing various methods for assessing horizontal activity, the findings were consistent on some occasions but inconsistent on others. These data already indicate differences in the performance of the multiple methods, which we further evaluated using ROC analysis. However, in this context, it is essential to note that the influencing factors and the information collected vary across the methods applied. A primary aspect is that scoring can be influenced by the presence of the caregiver or experimenter in the room^71,77^. Thus, the interpretation of Manual Scores needs to consider that rodents are flight animals that tend to hide signs of pain and anxiety in the presence of caregivers or experimenters^71,78^. On the other hand, the OF data reflect the animal’s reaction to a novel environment, with the early phase of exposure influenced by exploratory behavior and novelty-associated anxiety levels^79^. Thus, this test can be more sensitive in specific distress scenarios. In this context, it is noteworthy that Zieglowski and colleagues (2020)^56^ have already pointed out that the OF test proved to be the most suitable among three behavioral paradigms for providing information about the post-surgical burden following laparotomy in rats.

Taken together, our meta-analysis across different surgical procedures supports horizontal activity as a valuable readout parameter in multidimensional scoring approaches. This finding aligns with the outcomes of earlier studies focused on specific interventions and the integration of activity-related parameters in various multivariate scoring systems designed to assess post-surgical burden, including pain^18,19,30,53,72^.

In addition to surgical interventions, we had access to some selected datasets that analyzed the impact of different tests and techniques applied in neuroscientific and stress research. Among these, the restraint stress paradigm in mice and the FST in rats exerted relevant effects on activity in the OF or running wheel, respectively. These data suggest that a reduction in activity may reflect the psychological distress associated with these experimental situations. A relevant drop in activity patterns has already been reported as a consequence of exposure to other sub-chronic to chronic stress paradigms in mice, including unpredictable chronic mild stress and chronic social defeat stress^80,81^, whereas restraint stress has also been reported to cause transient increases in locomotor activity^82^.

To our surprise, tethered recording techniques in rats had no significant influence on activity, as assessed by Clinical Scores and home-cage telemetry, arguing against the notion of a relevant mechanical restraint and pronounced distress. This latter finding aligns with the conclusion from the original study^21^, which only identified subtle effects on circadian rhythmicity.

Analysis of datasets across various non-genetic disease models induced by chemical compounds, electrical stimulation, or cancer cells injection revealed frequent alterations in activity patterns, as assessed by different techniques and methods. Notably, the bidirectionality of the alterations in locomotion requires attention, which is often not reflected in simple clinical scoring systems that focus on hypolocomotion as a possible consequence of distress and pain^71,72,83^.

A disease-associated decline in activity and locomotion was evident in datasets from the colitis, late-stage glioma, and Parkinsonism models. While the hypolocomotion might be directly related to the motor dysfunction associated with the symptom spectrum of Parkinson’s disease^84,85^, it probably reflects the general state of sickness related to colitis, resulting in fatigue, loss of appetite, nausea, weight loss, and abdominal pain. Additionally, hypolocomotion in the late-stage glioma model may be associated with the fast growing malignant brain tumor and, depending on its location, with subsequent sudden onset headache, nausea, fatigue, confusion, weight loss, and mood changes^52,86,87^. Notably, the colitis model has served as a basis to validate the assessment of VWR as an informative readout parameter for severity assessment^8,10^. In the glioma model, the original study reported a late decline of locomotor activity two days prior to the humane endpoint^52^. Interestingly, the readout parameter was outperformed by body weight data as an early indicator of deterioration in well-being and for predicting humane endpoints^52^, as weight loss was observed in 100% of all rats at humane endpoint, whereas locomotor activity declined on group level but not in all rats.

In apparent contrast, several models were associated with hyperlocomotion detected by different techniques. These comprised various epilepsy models and a model with hearing loss induced by injection of an ototoxic compound. Considering other reports focused on these models, these findings seem neither study- nor laboratory-specific^88–91^. The number of models associated with increased locomotor activity underscores the conclusion that hyperlocomotion should not be overlooked in the context of severity assessment. Higher levels of activity and locomotion can theoretically reflect a state of increased nervousness, and when reaching extreme levels, even restlessness. Thus, scoring systems and other approaches to assess locomotion should not only focus on a possible reduction, but also capture detailed information about a potential increase.

Our analysis of genetic models of neurodevelopmental and neuropsychiatric disorders supports this conclusion. While there were differences between sexes and age phases, hyperlocomotion was a frequent finding in the three models analyzed. The need to carefully consider age as an influencing factor has earlier been demonstrated by a comprehensive study assessing wheel running and distance moved in the OF^92^. This fact, for instance, should be considered when designing approaches aimed at determining the cumulative burden of models of neurodevelopmental disorders.

A key question for assessment of horizontal activity in the context of both research questions and severity assessment is the selection of methods and techniques. To address this question, we used activity during the dark phase, monitored by radiotelemetry or video-based tracking, as the “gold standard”. The “gold standard” provides precise information about the locomotor behavior of animals during their active phase, when activity levels are highest. In comparison with the “gold standard”, the performance of both scoring systems, the Clinical Score and the more specific and detailed Neuroscore, was generally lower across the interventions and models analyzed (Fig. 6, Supplementary Figure S36-S40, Supplementary Table S17). Moreover, this observation remained true when using alternative references (light phase and complete day). These data demonstrate that common clinical scoring systems, which are routinely applied, as well as more sophisticated scoring systems, may often miss relevant alterations in locomotor activity. Interestingly, this was true for intervention and disease-associated decreases and increases in horizontal activity. In this context, it must be considered that rodents are flight animals that tend to hide pain and weakness in the presence of observers^71,78^. As a consequence, observer-dependent readout parameters can be biased in a relevant manner^28,71,77^.

Along this line, tracking of distance moved in an OF arena also did not show high sensitivity for detecting changes in locomotor activity that can be identified by home cage telemetry or video-based tracking. The lack of a relevant correlation between OF data and home cage monitoring probably reflects the fact that the informative value of OF analysis is influenced by exposure to the novel environment, which can be associated with increased anxiety levels, and by exploratory behavior patterns in addition to basal activity levels of the animals^79^. Thus, continuous tracking or telemetric recording of home-cage activity cannot be replaced by brief monitoring in an OF arena. Notably, respective differences between the readout parameters have already been observed by van’t Land and Hendriksen (1995)^93^, who reported a decrease in nocturnal home-cage activity, but a lack of changes in OF behavior in mice with intraperitoneal injection of Freund’s complete adjuvant.

In this context, it is emphasized that OF data can be of additional relevance as changes in exploration of novel environments can occur as a consequence of distress or pain^22,94,95^.

Although it did not achieve a perfect discrimination, VWR demonstrated the best performance compared to the “gold standard” method in the analyzed example (Fig. 6C-D, Supplementary Figure S37, Supplementary Table S17). Thus, it seems to be the best alternative to continuous video-based tracking or telemetric approaches, which are limited by the invasive nature of the transmitter implants. VWR has been validated in a stress paradigm and various induced and genetic disease models^8–10,80,96^. One drawback is that some reports describe increased levels of stereotypies or aggression in certain strains of mice when exposed to running wheels^97,98^. Thus, care should be taken to control for potential detrimental effects.

The interpretation of the ROC and cross-correlation analyses needs to consider that some of the datasets were generated during the dark phase, i.e. the activity phase of the animals (wheel running and tracking during this phase), and other datasets were collected during the light phase, i.e. the resting phase of the animals (scores, OF, as well as wheel running and tracking during this phase). While changes during the dark phase may better reflect direct disease-associated alterations in activity, data from the light phase can provide information about disturbances in resting and sleep patterns. In this context, we aimed to quantitatively analyze differences in the outcomes between analyses during the light phase and those from tracking (telemetric or video-based) during the dark phase. As we failed to identify a strong correlation between the respective parameters, this comparative analysis confirmed relevant differences in the informative value. This was further validated by lower detection rates when using light-phase and complete day activity data as alternative references in the Intracerebral Electrode Implantation and Epilepsy Model – Post SE studies. A higher sensitivity of locomotor activity during the dark phase compared to that during the light phase has also been reported by other groups investigating the influence of a lung cancer model and a model of myotonic dystrophy type 1^99,100^.

This difference in the informative value of data from dark versus light phases needs to be carefully considered in the context of applying common scoring systems or other methods for severity assessment, which are routinely conducted during the daytime, corresponding to the light phase in most animal facilities. The best option to address this issue is the use of continuous monitoring procedures in the home-cage, allowing 24/7 analysis^11–15,17,40,101–103^, avoiding a restriction to data collected during the resting phase of the animals and avoiding the presence of an observer as a confounding factor. However, the application of these systems in routine monitoring is still facing challenges related to costs and data management.

As we and others have pointed out, it is of utmost importance to keep in mind that a sensitive and robust severity assessment can only be built on an evidence-based design of multidimensional approaches that integrate various readout parameters in an animal-, model/intervention-, and laboratory-specific manner. This has been reflected by the successful design of multivariate schemes, such as the RELative Severity Assessment (RELSA) or the Composite Measure schemes (CMS), which have, among other parameters, integrated activity data (e.g., Talbot et al., 2022^19^; van Dijk et al.^104^; Reiber et al., 2023^105^; Munk et al., 2024^30^), Along this line, Wassermann and colleagues^17^ have demonstrated that integrating only one additional parameter (heart rate) in addition to activity data can already take classification of severity to another level.

In line with the need to integrate various readout parameters into multidimensional schemes, we would like to acknowledge that a more comprehensive analysis of complex activity patterns, such as rearing, grooming, climbing, cage lid hanging, social interaction, burrowing, and nest building, is of particular interest. The development of various automated video analysis tools, built on machine learning and computer vision algorithms^106–109^, is paving the way for the application of comprehensive, continuous monitoring approaches. However, their broad implementation still faces the challenges listed above.

Across a wide range of surgical interventions, stress paradigms, and disease models, reductions or increases in horizontal activity have emerged as sensitive indicators of distress, pathology, or altered physiological states. Traditional scoring systems and short behavioral tests, such as the OF, often showed lower sensitivity in detecting these changes than continuous home-cage telemetry or video tracking during the animals’ active (dark) phase. VWR provided a more reliable alternative but remained less sensitive than continuous monitoring. As a limitation, we would like to emphasize that direct comparisons across techniques were limited to a subset of models, interventions, and studies.

In addition, we would like to highlight that our analysis is based on real-world data generated in laboratories across Germany with diverse hypotheses and research foci. This was inherently associated with heterogeneous methodologies that could affect the animals’ activity, and we could not control them. Among these are: (1) the “total distance moved” in the OF took into account the total duration of exposure, without distinguishing between exploration and locomotion; (2) for some of the parameters analyzed, the measurement included aggregated data from both the day and night phases; and (3) heterogeneous postsurgical analgesic regimens among the included studies.

Nevertheless, our findings emphasize the need for continuous monitoring techniques applied in the home cage, thereby avoiding confounding factors such as time of day and the presence of an observer. These approaches need to be embedded in evidence-based multidimensional severity assessment strategies that integrate continuous activity measures with additional physiological and behavioral parameters.

## Supplementary Information

Below is the link to the electronic supplementary material.

Supplementary Material 1

## Data Availability

The data used in the current study are publicly available in the respective publications, no additional raw data were generated. All other summaries and processed data are within the paper and its Supplementary files.
