# Supplementary material for: Activity as a readout parameter for neurobehavioral research and severity assessment in laboratory rodents: a systematic comparison and meta-analysis of measurement techniques
Source: Sci Rep. 2026 Aug 21;16:26320. doi: 10.1038/s41598-026-65396-6 (PMC13498556; doi:10.1038/s41598-026-65396-6)
Supplement: Supplementary file 1 [file 41598_2026_65396_MOESM1_ESM.docx]

**Activity as a Readout Parameter for Neurobehavioral Research and Severity Assessment in Laboratory Rodents: A Systematic Comparison and Meta-analysis of Measurement Techniques**

Daniel Pérez-Pérez, Miriam Maihöfner, Anna Munk, Maria Reiber, Anika Bach-Hagemann, André Bleich, Peter Gass, Anna Hartmann, Christine Häger, Marie Johne, Simone Kumstel, Ute Lindauer, Anne S. Mallien, Kerstin Schwabe, Leonie Tix, René H. Tolba, Laura Warner, Dietmar Zechner and Heidrun Potschka

**Supplementary information**

*Supplementary figures*

*Supplementary tables*

*Supplementary references*

Figure S1. Scheme of the workflow for study selection and data analysis.

The collection of studies from the DFG FOR 2591 consortium was screened to select relevant datasets based on the model, species, and readout parameters analyzed. Cooperation groups were contacted to gather additional studies or to complete the data from selected studies. Data were pre-processed and organized for analysis. Effect size calculation comparing relevant experimental/intervention groups to respective reference data was performed for all studies. We considered a more detailed set of criteria (based on data quality and completeness, methodological approaches, and overall comparability of the different studies) to select studies for meta-analysis, ROC analysis, and correlation analysis. ROC, Receiver Operating Characteristic. Created in BioRender. Potschka, H. (2026) https://BioRender.com/9jjmeag

Figure S2. Scheme of the process of selection of the “gold standard” or reference data, threshold values, and classification of observations.

The preparation of data for Receiver Operating Characteristic (ROC) analysis consisted of three steps: “gold standard’ or reference data decision, threshold selection, and classification of observations. S.D., Standard Deviation; Q, quartile.

Figure S3. Effects of intracerebral electrode implantation in mice on activity assessed by Clinical Score and the Neuroscore.

Animals receiving the same analgesic scheme but not operated were considered as control (Drug control). Cohen’s H (± 95% CI) effect size for the Clinical Score (top row) and Neuroscore (bottom row) considering the naïve group as reference. CI, Confidence Interval; N; Non-steroidal anti-inflammatory drugs; L, Local anesthetic; O, Opioids. n = 8 per group.

Figure S4. Effects of intracerebral electrode implantation in mice on activity assessed by voluntary wheel running (VWR).

Animals receiving the same analgesic scheme but not operated were considered as control (Drug control). Hedge’s G (± 95% CI) effect size for running distance assessed by the VWR during the light (top row) and dark phase (bottom row) considering the naïve group as reference. CI, Confidence Interval; N; Non-steroidal anti-inflammatory drugs; L, Local anesthetic; O, Opioids. n = 8 per group.

Figure S5. Effects of intracerebral electrode implantation in mice on activity assessed by home-cage-based video monitoring.

Animals receiving the same analgesic scheme but not operated were considered as control (Drug-control). Hedge’s G (± 95% CI) effect size for locomotion assessed by home-cage-based video monitoring during the light (top row) and dark phase (bottom row) considering the naïve group as reference. CI, Confidence Interval; N; Non-steroidal anti-inflammatory drugs; L, Local anesthetic; O, Opioids. n = 8 per group.

Figure S6. Effects of intraperitoneal transmitter implantation in mice on activity assessed by telemetry.

Hedges’ G (± 95% CI) effect size for locomotion assessed by telemetry considering baseline data (bsl, i.e., day 28) as reference (dark phase). CI, Confidence Interval; n = 5-7 per group.

Figure S7. Effect of the induction of a subarachnoid hemorrhage (SAH) in rats on activity assessed by Clinical Score, the Neuroscore, and in the Open field.

Cohen’s H (± 95% CI) effect size for the Clinical Score (a and b) and Neuroscore (c and d) and Hedge’s G (± 95% CI) effect size for distance moved in the Open field (e and f) considering the sham or the drug-control (anesthesia) groups or baseline data (bsl, i.e., day -1) as reference. Animals receiving the same analgesic/anesthetic scheme but not operated were considered as drug-control (anesthesia). CI, Confidence Interval. n = 5 for anesthesia only, 9-19 for sham, and 6-20 for SAH – filament.

Figure S8. Effect size of the Cerebellar Cognitive Affective Syndrome Model in rats on activity assessed in the Open field.

Hedge’s G (± 95% CI) effect size for distance moved in the Open field considering the control groups (naïve or sham, a) or baseline data (bsl, b) as reference. CI, Confidence Interval. n = 18-22 per group.

Figure S9. Effects of partial hepatectomy (PH) in rats on activity assessed by Clinical Score and in the Open field.

Cohen’s H (± 95% CI) effect size for the Clinical Score considering the laparotomy group as reference (a, only male). Hedge’s G (± 95% CI) effect size for distance moved in the Open field considering baseline data (bsl, i.e., mean of day -2 and -1; female: b, male: c) as reference. CI, Confidence Interval. n = 10 for male laparotomy, 7 for female PH 50%, 21-63 for male PH 50%, and 18-63 for male 70%.

Figure S10. Effects of partial hepatectomy (PH) in rats receiving different analgesia schemes on activity assessed in the Open field.

Hedge’s G (± 95% CI) effect size for distance moved in the Open field considering baseline data (bsl, i.e., mean of day -2 and -1) as reference. CI, Confidence Interval. n = 6-7 per group.

Figure S11. Effects of partial hepatectomy (PH) in rats on activity assessed by Clinical Score and telemetry.

Cohen’s H (± 95% CI) effect size of the Clinical Score (a) and Hedges’ G (± 95% CI) effect size for locomotion assessed by telemetry (b, dark phase) considering baseline data (bsl, i.e., day -2) as reference. Data from day two in the 50% partial hepatectomy group and day six in the 70% partial hepatectomy group had to be excluded due to over 20% missing data (b). CI, Confidence Interval. n = 6-10 per group.

Figure S12. Effects of repetitive Restraint stress in mice on activity assessed by voluntary wheel running (VWR).

Hedge’s H (± 95% CI) effect size for running distance assessed by VWR considering either the control group (a) or baseline data (bsl, i.e., day -1, b) as reference. CI, Confidence Interval. n = 8 per experimental group.

Figure S13. Effects of the exposure to the Forced Swim Test (FST) in rats on activity assessed in the Open field.

Hedge’s G (± 95% CI) effect size for distance moved in the Open field considering the different control groups (i.e., Naïve and Sham, a) or pre-FST values (bsl, i.e., day -3, b) as reference. The sham group consisted of animals undergoing the FST and receiving an additional intraperitoneal saline injection. CI, Confidence Interval. n = 24 per group.

Figure S14. Effects of tethered and non-tethered recording techniques in rats on activity assessed by telemetry.

Hedges’ G (± 95% CI) effect size for locomotion assessed by telemetry considering baseline data (bsl, i.e., mean of day -2 and -1) as reference (dark phase). CI, Confidence Interval. Non-tethered: n = 6, Tethered: n = 7.

Figure S15. Effects of hippocampal and amygdala (BLA) kindling in mice on activity assessed by the Neuroscore and in the Open field.

Cohen’s H (± 95% CI, a) effect size for the Neuroscore and Hedge’s G (± 95% CI, b) effect size for distance moved in the Open field considering each of the naïve or sham group as reference. CI, Confidence Interval. n = 11-15 per group.

Figure S16. Effects of the intrahippocampal kainate (IHK) model of temporal lobe epilepsy in rats on activity assessed by Neuroscore and in the Open field.

Cohen’s H (± 95% CI, a) effect size for the Neuroscore and Hedge’s G (± 95% CI, b) effect size for distance moved in the Open field considering the naïve, sham, or sham (tm) group as reference. Animals in the Sham (tm) group received only the implantation of a subcutaneous transmitter, but no intracerebral electrode implant. CI, Confidence Interval; Sham (tm), Transmitter only sham. n = 8-12 per group.

Figure S17. Effects of the intrahippocampal kainate (IHK) model of temporal lobe epilepsy in mice on activity assessed by Clinical Score and telemetry.

Cohen’s H (± 95% CI) effect size of the Clinical Score (a) and Hedges’ G (± 95% CI) effect size of the locomotion assessed by telemetry (light phase: b; dark phase: c) considering the respective sham groups as reference. Animals of the Sham (tm) group received only the implantation of a subcutaneous transmitter, but no intracerebral electrode implant. When two timepoints are listed in a single tick of the x-axis, we only had access to the mean of both respective experimental days. CI, Confidence Interval; Sham (tm), Transmitter only sham. n = 8-12 per group.

Figure S18. Effects of repetitive Dextran Sulfate Sodium (DSS) administration in mice on activity assessed by voluntary wheel running (VWR).

Hedge’s G (± 95% CI) effect size for running distance assessed by VWR considering the DSS 0% group (Sham) (a and c) or baseline data (bls, i.e., day 0, b and d) as reference. Animals were only handled (top row) or exposed to blood sampling by a phlebotomy (bottom row). CI, Confidence Interval. n = 7-8 for DSS 0%, 8-14 for DSS 1%, and 8-9 for DSS 1.5%.

Figure S19. Effects of Dextran Sulfate Sodium (DSS) administration in mice on activity assessed by Clinical Score and voluntary wheel running (VWR).

Cohen’s H (± 95% CI) effect size for the Clinical Score (a) and Hedge’s G (± 95% CI) effect size for running distance assessed by VWR (b) considering baseline data (bsl, i.e., day -1) as reference. CI, Confidence Interval. n = 12.

Figure S20. Effects of orthotopic pancreatic cancer cell implantation and tumor growth in mice on activity assessed by Clinical Score and telemetry.

Cohen’s H (± 95% CI) effect size of the Clinical Score (a) and Hedges’ G (± 95% CI) effect size for locomotion assessed by telemetry (b) considering baseline data (bsl, i.e., day -1) as reference. CI, Confidence Interval. n = 7-10.

Figure S21. Effects of amygdala kindling in rats on activity assessed in the Open field.

Hedge’s G (± 95% CI) effect size for distance moved in the Open field considering the naïve or sham group as reference. CI, Confidence Interval. n = 12 per group.

Figure S22. Effects of amygdala kindling in rats on activity assessed by telemetry.

Hedges’ G (± 95% CI) effect size for locomotion assessed by telemetry considering the respective sham groups (light phase: a; dark phase: b) or baseline data (bsl, i.e., mean of day -3 and -2; dark phase: c) as reference. Animals of the Sham (tm) group received only the implantation of a subcutaneous transmitter, but no intracerebral electrode implant. Data from the kindling group of the dark phase of day three had to be excluded due to over 20% missing data (b and c). CI, Confidence Interval. n = 5-6 per group.

Figure S23. Effects of the pilocarpine-induced post-status epilepticus model of epilepsy in rats on activity assessed by the Neuroscore and in the Open field.

Cohen’s H (± 95% CI, a) effect size for the Neuroscore and Hedge’s G (± 95% CI, b) effect size for distance moved in the Open field considering the naïve or sham group as reference. Original data were generated in three different cohorts (Tethered, PET, and Telemetry) and analyzed accordingly. CI, Confidence Interval; PET, Positron Emission Tomography. n = 12-15 (tethered cohort), 12-13 (PET cohort), and 6-11 (Telemetry cohort) per group.

Figure S24. Effects of the pilocarpine induced post-status epilepticus model of epilepsy in rats on activity assessed by telemetry.

Hedges’ G (± 95% CI) effect size for locomotion assessed by telemetry considering the sham group (light phase: a; dark phase: b) or baseline data (bsl, i.e., mean of day -3 and -2; dark phase: c) as reference. When two timepoints are listed in a single tick of the x-axis, we only had access to the mean of both respective experimental days. CI, Confidence Interval. Sham: n = 6, Pilocarpine SE: n = 11.

Figure S25. Effects of the electrically induced post-status epilepticus model of epilepsy in rats on activity assessed by Clinical Score, the Neuroscore, and in the Open field.

Cohen’s H (± 95% CI) effect size for the Clinical Score (a) and Neuroscore (b), and Hedge’s G (± 95% CI) effect size for distance moved in the Open field (c) considering the naïve or sham group as reference. Original data were generated in three different cohorts (Tethered, PET, and Telemetry) and analyzed accordingly. CI, Confidence Interval; PET, Positron Emission Tomography. n = 10-11 (tethered cohort), 8-12 (PET cohort), and 6-7 (telemetry cohort) per group.

Figure S26. Effects of the electrically induced post-status epilepticus model of epilepsy in rats on activity assessed by telemetry.

Hedges’ G (± 95% CI) effect size for locomotion assessed by telemetry considering the sham group (light phase: a; dark phase: b) or baseline data (bsl, i.e., mean of day -2 and -1; dark phase: c) as reference. When two timepoints are listed in a single tick of the x-axis, we only had access to the mean of both respective experimental days. CI, Confidence Interval. Sham: n = 6, Electrical SE: n = 7.

Figure S27. Effects of hearing loss in rats on activity assessed in the Open field.

Hedge’s G (± 95% CI) effect size for distance moved in the Open field considering the naïve or sham group (a-c) or baseline data (bsl, d) as reference. Original data were generated from three different cohorts (Adult 2nd cohort [a]; juvenile [b]; and Adult 1st cohort [c and d]).CI, Confidence Interval. n = 8-12 (juvenile cohort), 10-11 (adult cohort 1), and 4-12 (adult cohort 2) per group.

Figure S28. Effects of the 6-Hydroxydopamine (6-OHDA) injection and subsequent intracerebral electrode implantation in rats on activity assessed in the Open field.

Hedge’s G (± 95% CI) effect size for distance moved in the Open field considering the vehicle group (per surgery, a) or baseline data (bsl, i.e., day -1, b) as reference. Animals were initially intracerebrally injected and, after 4 weeks, implanted with an intracerebral electrode. CI, Confidence Interval. n = 7-10 per group.

Figure S29. Effects of implanting glioma cells and subsequent tumor resection in rats on activity assessed in the Open field.

Hedge’s G (± 95% CI) effect size for distance moved in the Open field considering baseline data (bsl, i.e., day -1) as reference. Animals were initially intracerebrally injected with glioma cells and, after 1 week, the generated tumor was resected. CI, Confidence Interval. n = 8

Figure S30. Effects of glioblastoma formation during the phase with progressive clinical deterioration approaching the humane endpoint in rats on activity assessed by Clinical Score and in the Open field.

Cohen’s H (± 95% CI) effect size for the Clinical Score (a) and Hedge’s G (± 95% CI) effect size for distance moved in the Open field (b) considering baseline data (bsl, i.e., day -8) as reference. CI, Confidence Interval. n = 5-9 per group.

Figure S31. Effects of glioblastoma formation during the phase with progressive clinical deterioration approaching the humane endpoint in rats on activity assessed by Clinical Score and telemetry.

Cohen’s H (± 95% CI) effect size of Clinical Score (a) and Hedges’ G (± 95% CI) effect size for locomotion assessed by telemetry (dark phase: b) considering baseline data (bsl, approximately day -17, i.e. day -3 prior intracranial tumor cell injection) as reference. CI, Confidence Interval. n = 7.

Figure S32. Effect of Dravet Syndrome in mice on activity assessed by the Neuroscore, in the Open field, and by home-cage-based video monitoring.

Cohen’s H (± 95% CI) for the Neuroscore (a) and Hedge’s G (± 95% CI) for distance moved in the Open field (b) and for locomotion assessed by home-cage-based video monitoring (c) considering the *Scn1a*^+/+^ littermates as reference. CI, Confidence Interval; WT, Wildtype. n = 20 per group and developmental phase (10 female and 10 male).

Figure S33. Effect of the *Gria1* Knockout in mice on activity assessed by the Neuroscore, in the Open field, by voluntary wheel running (VWR), and by home-cage-based video monitoring.

Cohen’s H (± 95% CI) for the Neuroscore (a) and Hedge’s G (± 95% CI) for the distance moved in the Open field (b), running distance assessed by VWR (c), and locomotion assessed by home-cage-based video monitoring (d) considering wildtype littermates as reference. CI, Confidence Interval. n = 18-20 per group and developmental phase (10 female and 8-10 male).

Figure S34. Effects of the genetic knock out of the dopamine transporter in rats on activity assessed by the Neuroscore and in the Open field.

Cohen’s H (± 95% CI) for Neuroscore (A) and Hedge’s G (± 95% CI) for distance moved in the Open field (B) considering wildtype animals as reference. CI, Confidence Interval. n = 16 – 20 per genotype.

Figure S35. Receiver Operating Characteristic (ROC) curves and Cross-correlation plots for the Hepatectomy study.

ROC analysis was conducted for the different post-surgical days in the study. Sensitivity and specificity values to identify hypolocomotion using the Open field during the baseline period (a). Cross-correlation was also analyzed for the two post-surgical days (b = day 1; c = day 3).

Figure S36. Area under the curve (AUC) and 95% Confidence Intervals (CI) across Receiver Operating Characteristic (ROC) curves from different reference data in the Intracerebral Electrode Implantation.

ROC analysis was conducted for different directions of activity changes, using the dark phase (“gold standard”), light phase, and the complete day (i.e., the sum of the dark and light phases) as the reference data. VWR, Voluntary wheel running; HC, Home cage.

Figure S37. Receiver Operating Characteristic (ROC) curves for the Intracerebral Electrode Implantation.

ROC analysis was conducted for the different directions of activity changes. Sensitivity and specificity values to identify hyperlocomotion in female (a) and male (b) mice during the post-surgical period. VWR, Voluntary wheel running; HC, Home cage.

Figure S38. Receiver Operating Characteristic (ROC) curves and Cross-correlation plots for the post-status epilepticus studies (pilocarpine and electrical)

ROC analysis was conducted for the different directions of activity changes. Sensitivity and specificity values to identify hyperlocomotion during the post-surgical period (a). Cross-correlation was calculated across all activity-related readout parameters (b).

Figure S39. Area under the curve (AUC) and 95% Confidence Intervals (CI) across Receiver Operating Characteristic (ROC) curves from different reference data in the post-status epilepticus studies (pilocarpine and electrical)

ROC analysis was conducted for the different directions of activity changes, using the dark phase (“gold standard”) and the complete day (i.e., the sum of the dark and light phases) as the reference data. The detection rate when using the light phase activity data was low (17.9%), and ROC curves were not calculated.

Figure S40. Receiver Operating Characteristic (ROC) curves and Cross-correlation plots for the genetic models of neurological diseases (Dravet Syndrome and *Gria1* KO).

ROC analysis was conducted for the different directions of activity changes and developmental phases of the animals. Sensitivity and specificity values to identify hypolocomotion (a) and hyperlocomotion (b-c) in prepubescent (a-b) and sexually mature (c) genetically modified mice. Correlation was calculated across all evaluated activity-related readout parameters for prepubescent (d) and sexually mature (e) mice. HC, Home cage.

Table S1. Methodological details for all studies with telemetric data.

| **Study ID** | **Type of transmitter and implantation site** | **Software**  **- Acquisition**  **- Analysis** | **Sample size per group** | **Analyzed timeframes** | **Experimental days with available data** | **Day/value considered as Baseline for analysis** | **Intervention**  **(Day 0)** |
| --- | --- | --- | --- | --- | --- | --- | --- |
| Intraperitoneal Transmitter Implantation | ETA-F10; DSI, St Paul, MN, USA (i.p.) | Ponemah v. 5.20  Ponemah v. 5.20, Data Sciences International, USA | Carprofen n = 7  Metamizole n = 6 | Mean of 12 h over dark phase (including 2 h of light phase) | 0, 1, 2, 3, 4, 5, 7,14, 28 | 28 | Transmitter implantation |
| Partial Hepatectomy (PH) | HD-S11; DSI, St Paul, MN, USA (s.c.) | Ponemah v. 6.30  Ponemah v. 6.4, Data Sciences International, USA | 50% PH n = 10  70% PH n = 7 | Mean of 16 h over dark phase (including 4 h of light phase) | -2, 1, 2, 3, 6 | -2 | Partial hepatectomy |
| Epilepsy Model – Kindling (tele) | ETA-F10; DSI, St Paul, MN, USA (s.c.) | Ponemah v. 5.20  Ponemah v. 6.30, Data Sciences International, USA | Sham (tm) n = 6  Sham n = 6  Kindling n = 6 | Mean of light and dark phase | -3, -2, 1 (only dark phase), 2, 3, 14 (only dark phase), 15, 16 | Mean of day  -3 and -2 | Beginning of Amygdala kindling |
| Epilepsy Model – Post SE (IHK)* | HD-X02; DSI, St Paul, MN, USA (s.c.) | Ponemah v. 5.20  Ponemah v. 6.41, Data Sciences International, USA | Sham (tm) n = 8  Sham n = 8  IHK n = 12 | Mean of light and dark phase | 1, 2, 3, 4 (“post-SE”), mean of 14 and 15 (“2 weeks post-SE”), mean of 28 and 29 (“4 weeks post-SE”) |  | Surgical implantation of an intracerebral electrode, intracerebral injection of kainic acid (SE induction), and subcutaneous transmitter implantation |
| Epilepsy Model – Post SE (pilo) | HD-X02; DSI, St Paul, MN, USA (s.c.) | Ponemah v. 5.20  Ponemah v. 6.41, Data Sciences International, USA | Sham n = 6  SE n = 11 | Mean of light and dark phase | -3, -2, mean of 5 and 6 (“early post-insult”),  mean of 26 and 27 (“latency), mean of 63 and 64 (“chronic”) | Mean of day  -3 and -2 | Chemical SE induction |
| Epilepsy Model – Post SE (electrical) | HD-X02; DSI, St Paul, MN, USA (s.c.) | Ponemah v. 5.20  Ponemah v. 6.41, Data Sciences International, USA | Sham n = 6  SE n = 7 | Mean of light and dark phase | -2, -1, mean of 5 and 6 (“early post-insult”),  mean of 26 and 27 (“latency), mean of 61 and 62 (“chronic”) | Mean of day  -2 and -1 | Electrical SE induction |
| Biopotential Recording Techniques | HD-X02; DSI, St Paul, MN, USA (s.c.) | Ponemah v. 5.20  Ponemah v. 6.41, Data Sciences International, USA | Non-tethered n = 6  Tethered n = 7 | Mean of light and dark phase | -2, -1, 0, 1, 2, 5, 6, 7, 11, 12, 13 | Mean of day  -2 and -1 | Beginning of tethered/non-tethered recordings |
| Glioma Model (endpoint) | ETA-F10; DSI, St Paul, MN, USA (s.c.) | Ponemah v. 5.2  Ponemah v. 6.41, Data Sciences International, USA | Glioma n = 8 | Mean of 8 h during dark phase | -8, -7, -6, -5, -4, -3, -2, -1, 0 | Approxi-mately day  -17 (i.e., day  -3 to cell implantation) | Reaching humane endpoint |
| Pancreatic Cancer Model | ETA-F10; DSI, St Paul, MN, USA (i.p.) | Ponemah v. 5.2, Data Sciences International, USA  Excel (Microsoft, Redmont, USA) | Pancreatic cancer n = 10 | Mean of dark phase | -1, 0, 1, 2, 3, 4, 18, 34 | -1 | Pancreatic cancer cell injection |

*, In this model we had full access to the raw data (including individual days for the “post-SE” phase); in the original publication, mean values per phase were reported. i.p., intraperitoneal; USA, United States of America; s.c., subcutaneous; tele, telemetry; tm, transmitter; IHK, intrahippocampal kainate; SE, Status epilepticus; pilo, pilocarpine. Baseline data include data from the end of the laboratory-specific recovery phase (note that telemetric data can only be collected following surgery). The unit of measurement for all the studies was counts per minute (counts/minute).

Table S2. Methodological details for the assessment of activity-related Clinical Scores in each study.

| **Study ID** | **Parameter(s) of the Clinical Score used for defining a change in activity*** | **Threshold for activity-related scoring** |
| --- | --- | --- |
| Intraperitoneal Transmitter Implantation | Activity:  4 - 5 Less active  6 Hardly active  6+ Lethargic  Aktivität:  4 – 5 Weniger aktiv  6 Kaum aktiv  6+ Lethargisch | Score ≥ 4 for decreased activity |
| Partial Hepatectomy | Spontaneous behavior and willingness to move:  1: minor deviations from normal behavior; protective posture of the operated limb during rearing  5: unusual behavior; motor abnormalities (stilt gait, protective posture of the operated limb in the gait); reduced willingness to move (only after impulsion)  10: isolation; apathy; strongly reduced willingness to walk; behavioral stereotypes/aggressiveness; coordination disorders  20: pain noise during grabbing; lethargy; no readiness to move; automutilation | Score ≥ 1 for decreased activity |
|  |  |  |
|  |  |  |
| DAT KO Model | 0 Species-typical movements, curious, normal posture  1 Reduced activity, slightly reduced reaction to environmental stimuli  2 Reduced activity, reduced reaction to environmental stimuli  3 No activity, no reaction to environmental stimuli and/or arched back  0 Arttypische Bewegungen, neugierig, normale Haltung  1 Reduzierte Aktivität, verhaltene Reaktion auf Umweltreize  2 Reduzierte Aktivität, herabgesetzte Reaktion auf Umweltreize  3 Keine Aktivität, keine Reaktion auf Umweltreize und/oder aufgekrümmter Rücken | Score ≥ 1 for decreased activity |
| Colitis Model (Lab 2) |  |  |
| Subarachnoid Hemorrhage Model | 0 Normal movement  1 Reluctance of movement, slightly abnormal walking movement  5 Lethargy, Apathy, abnormal walking movement  10 Strongly impaired movement  30 Not moving at all most of the time (>6h, repetitive surveillance) | Score ≥ 1 for decreased activity |
| Epilepsy Model – Kindling (hp and bla) | Behavior:  1 Reduced reaction, limited or excessive activity  2 Hunched posture, partial isolation from the group  3 Apathetic, no reaction or aggression during handling, isolation, occurrence of stereotypical behaviors  Motor:  1 Mobility reduced or increased, occurrence of altered movement patterns  2 Strongly limited movement, presence of paralysis on individual extremities  3 Total immobility  Verhalten:  1 Verminderte Reaktionen, eingeschränkte oder überhöhte Aktivität  2 Kauerhaltung, teilweises Absondern von der Gruppe  3 Apathisch, keine Reaktion o. Aggressivität bei Handling, Isolation, Auftreten stereotyper Verhaltensweisen  Motorik:  1 Bewegt sich reduziert o. übermäßig, Auftreten veränderter Bewegungsmuster  2 Stark eingeschränkte Bewegung, Vorliegen von Lähmungen an einzelnen Extremitäten  3 Völlige Bewegungslosigkeit | Score ≥1 in any of the categories (an annotation indicated directionality) |
| Epilepsy Model – Post SE (IHK) |  |  |
| Intracerebral Electrode Implantation |  |  |
| Epilepsy Model – Kindling (teth) | Behavior:  1 Reduced reaction, limited or excessive activity  2 Hunched posture, partial isolation from the group  3 Apathetic, no reaction or aggression during handling, isolation, occurrence of stereotypical behaviors  Motor:  1 Mobility reduced or increased, occurrence of altered movement patterns  2 Strongly limited movement, presence of paralysis on individual extremities or convulsions  3 Total immobility  Verhalten:  1 Verminderte Reaktionen, eingeschränkte oder überhöhte Aktivität  2 Kauerhaltung, teilweises Absondern von der Gruppe  3 Apathisch, keine Reaktion o. Aggressivität bei Handling, Isolation, Auftreten stereotyper Verhaltensweisen  Motorik:  1 Bewegt sich reduziert o. übermäßig, Auftreten veränderter Bewegungsmuster  2 Stark eingeschränkte Bewegung, Vorliegen von Lähmungen an einzelnen Extremitäten oder Krämpfe  3 Völlige Bewegungslosigkeit | Score ≥1 in any of the categories |
| Epilepsy Model – Kindling (tele) |  |  |
| Dravet Syndrome Model | Behavior:  1 Reduced reaction, limited or excessive activity  2 Hunched posture, partial isolation from the group  3 Apathetic, no reaction or aggression during handling, isolation, occurrence of stereotypical behaviors  Motor:  1 Mobility reduced or increased, occurrence of altered movement patterns  2 Strongly limited movement, presence of paralysis on individual extremities  3 Total immobility  Verhalten:  1 Verminderte Reaktionen, eingeschränkte oder überhöhte Aktivität  2 Kauerhaltung, teilweises Absondern von der Gruppe  3 Apathisch, keine Reaktion o. Aggressivität bei Handling, Isolation, Auftreten stereotyper Verhaltensweisen  Motorik:  1 Bewegt sich reduziert o. übermäßig, Auftreten veränderter Bewegungsmuster  2 Stark eingeschränkte Bewegung, Vorliegen von Lähmungen an einzelnen Extremitäten  3 Völlige Bewegungslosigkeit | Score ≥1 in any of the categories. |
| *Gria1* KO Model |  |  |
| Epilepsy Model – Post SE (pilo) |  |  |
| Epilepsy Model – Post SE (electrical) |  |  |
| Biopotential Recording Techniques |  |  |
| Parkinsonism Model | 2 active with some interruptions of activity; exploratory behavior  3 prolonged lack of activity, limited exploratory behavior upon external stimuli, intact muscle tonus  4 ataxia, no exploratory behavior, severely limited reaction to external stimuli, decreased muscle tone, hunched posture, ruffled fur, delayed righting reflex  5 no righting reflex, premortal stage | Score ≥2 for decreased activity |
| Glioma Model |  |  |
| Glioma Model (endpoint) |  |  |
| Hearing Loss Model |  |  |
|  |  |  |
|  |  |  |
| Cerebellar Cognitive Affective Syndrome Model |  |  |
| Pancreatic Cancer Model | III. Spontaneous behavior:  2: III-a the animal is passive or overactive  4: III-b pronounced apathy, hyperkinetic, or isolation  IV. Flight behavior after contact:  2: IV-a animal is passive or overactive  5: IV-b distinct apathy or hyperkinetic | Score ≥2 in any of the categories (an annotation indicated directionality) |

*, In cases where we provide two versions, the English version is an unofficial translation because we only had access to the original German version. Otherwise, the English version is provided. DAT, Dopamine Transporter; hp, hippocampus; bla, basolateral amygdala; IHK, intrahippocampal kainate; teth, tethered; tele, telemetry; SE, Status epilepticus; pilo, pilocarpine.

Table S3. Methodological details from each cooperation partner for the Open field data.

| **Study ID** | **Color, shape, and size of the arena** | **Recording/Evaluation software** | **Duration of recording** | **Experimental room variables**  **- Light intensity**  **- Cleaning between trials**  **- Min. habituation time** | **Training, retraining, and baseline measures** | **Test repetitions** |
| --- | --- | --- | --- | --- | --- | --- |
| Partial Hepatectomy | Dark Square  72 x 72 x 40 cm | NOLDUS EthoVision XT 14 | 10 min | 150 lux*  Inciding Perfekt 5%  10 min | Training (3 times)  Retraining (2 times, baseline) | 1-4 times |
|  |  |  |  |  |  |  |
|  |  |  |  | 150 lux*  Antifect® N liquid  10 min | Training (2 times, baseline) | 1-4 times |
| DAT KO Model | Gray Square  50 x 50 x 50 cm | NOLDUS EthoVision XT | 60 min | 25 lux  70% ethanol  30 min | Training (1 time, baseline) | 1 time |
| Colitis Model (Lab 2) | White Square  50 x 50 cm | NOLDUS EthoVision XT 8.0 | 10 min | 25 lux  70% ethanol  30 min | No training | 1 time |
| Forced Swim Test | Gray Square  50 x 50 x 50 cm | NOLDUS EthoVision XT 15 |  | 25 lux  70% ethanol  30 min | Training/  baseline  (1 time) | 2 times |
| Subarachnoid Hemorrhage Model | Gray Square  72 x 72 x 50 cm | NOLDUS EthoVision XT 15 | 10 min | No measure  Antifect®  No habituation | Training (4 times)  Retraining (1 time, baseline) | 4 times |
| Epilepsy Model – Kindling (hp and bla) | Black Cylindrical  60 Ø x 40 cm | NOLDUS EthoVision 8.5 | 10 min | 12 lux  0.1% acetic acid  30 min | No training | 1 time |
| Epilepsy Model – Post SE (IHK) |  |  |  |  |  |  |
| Dravet Syndrome Model | White Cylindrical  60 Ø x 40 cm | NOLDUS EthoVision 8.5 | 15 min | 20 lux  0.1% acetic acid  30 min | No training | 2 times |
| *Gria1* KO Model |  |  |  |  | No training | 2 times |
| Epilepsy Model – Post SE (electrical) | Black Cylindrical  85 Ø cm | NOLDUS EthoVision 8.5 | 10 min | 10-20 lux  0.1% acetic acid  30 min- | No training | 1 time |
| Epilepsy Model – Post SE (pilo) | Black Cylindrical  85 Ø cm | NOLDUS EthoVision 8.5 | 10 min | 10-20 lux  0.1% acetic acid  - | No training | 1 time |
| Epilepsy Model – Kindling (teth) |  |  |  |  |  |  |
| Parkinsonism Model | Dark Square  60 x 60 x 30 cm | TopScan TM 1.0, Clever Sys. | 10 min | 10-20 lux  10% Bacillol®  30 min | Training (2 times) | 4 times |
| Glioma Model |  |  |  |  |  |  |
| Hearing Loss Model | Dark Square  60 x 60 x 30 cm | TopScan TM 2.0, Clever Sys. | 10 min | 10-20 lux  10% Bacillol®  30 min | Training (2 times) | 14 times |
|  |  |  |  |  |  |  |
| Cerebellar Cognitive Affective Syndrome Model | Dark Squared  60 x 60 x 30 cm | TopScan TM 1.0, Clever Sys. | 10 min | 10-20 lux  10% Bacillol®  30 min | Training (1 time) | 9 times |

*, measured in the housing room 1 m above floor level, the other lux measurements were from the floor of the arena. DAT, Dopamine Transporter; hp, hippocampus; bla, basolateral amygdala; SE, Status epilepticus; IHK, intrahippocampal kainate; pilo, pilocarpine; teth, tethered.

Table S4. Methodological details from each cooperation partner for the voluntary wheel running data.

| **Study ID** | **Type of access to the wheel** | **Orientation and diameter of the wheel** | **Manufacturer** | **Recording/Analysis software** | **Duration of monitoring (specific times)** | **Habituation/Adaptation phase** |
| --- | --- | --- | --- | --- | --- | --- |
| Colitis Model (Lab 1) | Free | vertical  11.5 cm | Revolyzer 3TS system | Revolyzer 3TS system  DASYLab 11.0 | 20 h (12:00 - 08:00 h) | 14 days |
| Restraint Stress Model |  |  |  |  |  |  |
| Colitis Model (Lab 2) | Free | vertical  11.5 cm | NX-8441-675 | Tachometer | 24 h | 16 days |
| *Gria1* KO Model | Free | vertical  15 cm | PhenoWheel, Noldus | NOLDUS EthoVision XT 15 | 4 d (~24 h) | No habituation phase |
| Intracerebral Electrode Implantation | Free | vertical  15 cm | PhenoWheel, Noldus | NOLDUS EthoVision XT 15 | 4 d (24 h) | No habituation phase |

Table S5. Methodological details from each cooperation partner for the home-cage-based video monitoring.

| **Study ID** | **Type of system** | **Habituation/**  **Adaptation (h)** | **Software** | **Total duration of monitoring and measuring unit** | **Number of animals in the home cage** | **Enrichment** | **Reference data** |
| --- | --- | --- | --- | --- | --- | --- | --- |
| Intracerebral Electrode Implantation | PhenoTyper, Noldus, Wageningen, Netherlands | 0 | Ethovision XT 15 (Noldus, Wageningen, the Netherlands, RRID:SCR_000441 | 4 days (cm) | 1 | Bedding material, Nestlets,  Mouse house | Naïve |
| Dravet Syndrome Model | PhenoTyper, Noldus, Wageningen, Netherlands | 0 | Ethovision XT 15 (Noldus, Wageningen, the Netherlands, RRID:SCR_000441 | Prepubescence: 4 days (cm/hour)  Sexual maturity: 4 days (cm/hour) | 2 | Bedding material, Nestlets,  Mouse house, two drinking bottles | Wildtype (*Scn1a*^+/+^) |
| *Gria1* KO Model | PhenoTyper, Noldus, Wageningen, Netherlands | 60-64 | Ethovision XT 15 (Noldus, Wageningen, the Netherlands, RRID:SCR_000441 | Prepubescence: 4 days  Sexual maturity: 4 days (cm/hour) | 2 | Bedding material, Nestlets,  Mouse house, two drinking bottles | Wildtype |

Table S6. R-packages used for the analysis.

| **Analysis step** | **Package name** | **Version** | **Citation** |
| --- | --- | --- | --- |
| Data import, wrangling, preprocessing, and plotting | “tidyverse” | 2.0.0 | Wickham, et al., 2019^1^ |
| String operations | “glue” | 1.8.0 | Hester & Bryan, 2024^2^ |
| Effect size and 95% CI calculation | “effectsize” | 1.0.1 | Shachar, et al., 2020^3^ |
| Meta-analysis (calculation and plotting) | “meta” | 8.2-0 | Balduzzi, et al., 2019^4^ |
| ROC analysis (AUC and 95% CI) | “pROC” | 1.19.0.1 | Robin, et al., 2011^5^ |
| Plot composition | “patchwork” | 1.3.2 | Pedersen, 2025^6^ |
| Correlation matrices calculations | “Hmisc” | 5.2-4 | Harrell, 2025^7^ |
| Correlation heat maps | “corrplot” | 0.95 | Wie & Simko, 2024^8^ |
| Other statistical analyses (normality tests, summary statistics, etc.) | “rstatix” | 0.7.3 | Kassambara, 2025^9^ |

CI, Confidence Interval; AUC, Area under the curve.

Table S7. Funding, quality characteristics and legal aspects of the included studies.

| **Study ID** | **Reference** | **Ethical approval*** | **Funding** | **Randomization** | **Blinding** | **Sample size calculation** |
| --- | --- | --- | --- | --- | --- | --- |
| Colitis Model (Lab 1)  Restraint Stress Model | Häger et al., 2018^10^ | 15/1905 | DFG FOR 2591 (BL953/10-1) | Group allocation | Not blinded | Power analysis |
| Intraperitoneal Transmitter Implantation | Talbot et al., 2022^11^ | 15/1905 | DFG FOR 2591 (BL953/10-1; BL953/10-2; HA6483/1-2; BL953/11-1; BL953/11-2) | Group allocation | Not blinded | Power analysis |
| Partial Hepatectomy | Zieglowski et al., 2021^12^ | 84–02.04.2017.A304 | DFG FOR 2591 (TO 542/5-1) | Group allocation | Not blinded | Power analysis |
|  | Zieglowski et al., 2020^13^ | 84–02.04.2017.A304 | DFG FOR 2591 (TO 542/5-1) | Group allocation  Surgical procedure | Not blinded | Power analysis |
|  | Kümmecke et al., 2023^14^ | 84–02.04.2017.A304 | DFG FOR 2591 (TO 542/5-1; TO 542/6-1) | N.A. | Not blinded | Power analysis |
| DAT KO Model | Mallien et al., 2022^15^ | 35-9185-81-G-143-19 | DFG FOR2591 (GA-427/12-2; PO681/9-2)  ERANET (01EW1911 UNMET; EJPRD2019-220) | Order of acoustic trial (pseudorandom) | Blinded | Power analysis |
| Colitis Model (Lab 2) | Mallien et al., 2020^16^ | 35-9185-81-G-143-19 | DFG FOR2591 (project 05)  Ingeborg Ständer Foundation  Research Fund of the UPK Basel | Group allocation (pseudorandom) | Blinded | Power analysis |
| Forced Swim Test | Becker et al., 2023^17^ | 35-9185-81-G-15-21 | DFG FOR 2591 (GA-427/12-2; BL953/11-2; PO681/9-2) | Group allocation | Blinded | Power analysis |
| Subarachnoid Hemorrhage Model | Bach-Hagemann et al., 2023^18^ | 81-02.04.2017.A457 | DFG FOR2591 (LI 588/5-1; LI 588/5-2) | Group allocation (suspended) | Blinded with unblinding event | N.A. |
| Epilepsy Model – Kindling (hp and bla) | Boldt et al., 2021^19^ | ROB-55.2-2532.Vet_02-17-68 | DFG FOR 2591 (PO681/9‐2) | Cage allocation  Cage order | Blinded (one technique) | Power analysis |
| Epilepsy Model – Kindling (teth) | Möller et al., 2018^20^ | ROB-55.2-1-54-2531-119-14 | DFG FOR 2591 (PO681/9-1) | Group allocation  Experimental order | Blinded (one technique) | N.A. |
| Epilepsy Model – Kindling (tele) | Möller et al., 2019^21^ | ROB-55.2-1-54-2532-105-16 | DFG FOR 2591 (PO681/9-1) | Group allocation  Experimental order | Not blinded | N.A. |
| Epilepsy Model – Post SE (IHK) | Buchecker et al., 2023^22^ | ROB-55.2-2532.Vet_02-17-86 | DFG FOR 2591 (PO681/9‐2; BL953/11-2) | Group allocation  Order for behavioral tests | Blinded (one technique) | N.A. |
| Dravet Syndrome Model | Reiber et al., 2022^23^ | ROB-55.2-2532.Vet_02-19-157 | DFG FOR 2591 (PO681/9‐2) | Group allocation  Order for behavioral tests  Cage order | Blinded (except for one analysis) | Power analysis |
| *Gria1* KO Model | Reiber et al., 2022^24^ | ROB-55.2-2532.Vet_02-19-157 | DFG FOR 2591 (PO681/9-2; GA427/12-2) | Group allocation  Order for behavioral tests  Cage order | Blinded (except for one analysis) | Power analysis |
| Intracerebral Electrode Implantation | Munk et al., 2024^25^ | ROB-55.2-2532.Vet_02-19-157 | DFG FOR 2591 (321137804; GZ: PO681/9-2, 9-3; GZ: TA2072/1-1; GZ: BA5768/2-1; GZ: ME3737/24-1)  Projekt DEAL | Group allocation  Subgroup order for testing | Blinded analysis | Power analysis |
| Epilepsy Model – Post SE (pilo) | Koska et al., 2019^26^ | ROB-55.2-1-54-2532-105-16 | DFG FOR 2591 (PO681/9-2) | Group allocation | Not blinded | N.A. |
|  | Di Liberto et al., 2018^27^ |  |  |  |  |  |
| Epilepsy Model – Post SE (electrical) | Seiffert et al., 2019^28^ | 55.2-1-54-2532-105-2016 | DFG FOR 2591 (PO681/9-1) | Group allocation | Not blinded | N.A. |
|  | van Dijk et al., 2018^29^ | 55.2-1-54-2532-011-2015 |  |  |  |  |
| Biopotential Recording Techniques | Aulehner et al., 2022^30^ | ROB-55.2-2532.Vet_02-16-105 | DFG FOR 2591 (PO681/9-1; PO681/9-2) | Group allocation | N.A. | Power analysis |
| Parkinsonism Model | Riedesel et al., 2023^31^ | AZ 14/1533  AZ 16/2315 | DFG FOR 2591 (SCHW1176/7-1) | Group allocation  Testing order | Blinded (one analysis) | Power analysis |
| Glioma Model |  |  |  |  |  |  |
| Hearing Loss Model | Johne et al., 2022^32^ | AZ 18/2874 | DFG FOR 2591 (EXC 2177/1; Project ID 390895286) | Group allocation | Partially blinded | Power analysis |
|  | Jelinek et al., 2024^33^ | AZ 18/2874 | DFG FOR 2591 (EXC 2177/1; Project ID 390895286) | Group allocation | Blinded | Power analysis |
|  | Stenzel et al., 2025^34^ | AZ 18/2874 | EXC 2177/1; Project ID 390895286 | Group allocation | Blinded (one analysis) | Power analysis |
| Cerebellar Cognitive Affective Syndrome Model | Helgers et al., 2020^35^ | AZ 14/1533  AZ 16/2315 | Stiftung Neurochirurgische Forschung | Group allocation | Partially blinded | Power analysis |
| Glioma Model (endpoint) | Helgers et al., 2020^36^ | AZ 14/1533  AZ 16/2315 | DFG FOR 2591 (SCHW1176/7-1) | Behavioral testing | Partially blinded | Power analysis |
| Pancreatic Cancer Model | Kumstel et al., 2019^37^ | Az. 7221.3-1-062/16 | DFG FOR 2591 (321137804; ZE 712/1-1; VO 450/15-1) | N.A. | Not blinded | Power analysis |

*, According to the authors of the original studies all experiments were conducted in line with the Directive 2010/63/EU, listed license number of animal allowance by local authorities: LAVES, Darmstadt ethics committee for animal research, LANUK, Regierungspräsidium Karlsruhe, LANUV, Government of Upper Bavaria, or Mecklenburg-Vorpommern. DFG, Deutsche Forschungsgemeinschaft; FOR, Forschungsgruppe; DAT, Dopamine Transporter; hp, hippocampus; bla, basolateral amygdala; teth, tethered; tele, telemetry; SE, Status epilepticus; IHK, intrahippocampal kainate; pilo, pilocarpine.

Table S8. Methodological details of the animals, housing and husbandry, and reference data included in the analysis.

| **Study ID** | **Reference(s)** | **Animals** | | | **Housing and husbandry** | | | |
| --- | --- | --- | --- | --- | --- | --- | --- | --- |
|  |  | **Species** | **Strain** | **Sex** | **Cage**  **- Size/type**  **- Group size**  **- Enrichment(s)** | **Diet**  **- Type**  **- Access** | **Environment**  **- Temperature**  **- Humidity**  **- Light dark cycle** | **Handling**  **- Type**  **- Sex of caregiver(s) and experimenter(s)** |
| Intracerebral Electrode Implantation | Munk et al., 2024^25^ | Mouse | C57BL/6J | Female and male | Macrolon type III cage (Zoonlab)  Group- and single-housed  Nestlet, mouse house | Diet (ssniff Spezialdiäten GmbH) and tap water ad libitum | 20–24 °C  45–65%  12:12 h light/dark cycle | Tail  Females and males |
| Intraperitoneal Transmitter Implantation | Talbot et al., 2022^11^ | Mouse | C57BL/6J | Female | Macrolon type-II cage, 360 cm^2^ (Tecniplast)  Pair-housed  Nesting material | Standard rodent food (1324, Altromin) and autoclaved distilled water ad libitum | 21–23 °C  50–60%  14:10 h light/dark cycle | Tail  Only females |
| Subarachnoid Hemorrhage Model | Bach-Hagemann et al., 2023^18^ | Rat | Wistar | Male | Type 2000 cage (Tecniplast)  Temporal group-housed  Nestlet | Standard diet (V1534-300, ssniff Spezialdiäten GmbH) and water ad libitum | 20–24 °C  50–60%  12:12 h light/dark cycle | 1-hand grasping  Only females |
| Cerebellar Cognitive Affective Syndrome Model | Helgers et al., 2020^35^ | Rat | Sprague-Dawley | Male | Macrolon Type IV cage  Group-housed  Cardbox, Woodpieces | Standard rodent diet (Altromin) 14g per day and tap water ad libitum | 20–24 °C  45–65%  14:10 h light/dark cycle | -  Females and males |
| Partial Hepatectomy | Zieglowski et al., 2021^12^ | Rat | Wistar-Han | Male | Type 2000 filter-top cage (Tecniplast)  Group-housed  Nestlet, tunnel | Standard diet (V1534-300, ssniff Spezialdiäten GmbH) and sweetened (Ja! Süßstoff flüssig, Rio Mints & Sweeteners B.V.) drinking water ad libitum | 20–24 °C  30–70%  12:12 h light/dark cycle | Tunnel  Only females |
|  | Zieglowski et al., 2020^13^ |  |  |  |  |  |  |  |
|  | Kümmecke et al., 2023^14^ |  |  | Female and male | Type 2000 filter-top cage (Tecniplast)  Group-housed  Tunnel |  |  |  |
| Epilepsy Model – Kindling (hp and bla) | Boldt et al., 2021^19^ | Mouse | HsdWin:NMRI | Female | Macrolon Type III cage (Zoonlab)  Single-housed  Nestlet, mouse house | Diet (ssniff Spezialdiäten GmbH) and tap water ad libitum | 20–24 °C  45–65%  12:12 h light/dark cycle | Tail  Females and males |
| Epilepsy Model – Post SE (IHK) | Buchecker et al., 2023^22^ | Mouse | NMRI | Female | Macrolon type III cage (Zoonlab)  Single-housed  Nestlet, house | Diet (ssniff Spezialdiäten GmbH) and tap water ad libitum | 20–24 °C  45–65%  12:12 h light/dark cycle | Tail  Females and males |
| Colitis Model (Lab 1) | Häger et al., 2018^10^ | Mouse | C57BL/6J | Female | Macrolon cage, 360 cm^2^  Single-housed  Running wheels | Standard rodent food (1324, Altromin) and autoclaved water ad libitum | 21–23 °C  50–60%  14:10 h light/dark cycle | Tail  Only females |
| Colitis Model (Lab 2) | Mallien et al., 2020^16^ | Mouse | C57BL/6J | Male | Macrolon type II  Single-housed  Nesting material | Diet (LASQC diet Rod16 HiHyg, Altromin) and tap water ad libitum | 21–25 °C  45–55%  12:12 h light/dark cycle | Tail  Only females |
| Pancreatic Cancer Model | Kumstel et al., 2019^37^ | Mouse | C57BL/6J | Male | Type III cage  Single-housed  Nesting material, paper roll, wooden stick | Diet and tap water ad libitum | 19–23 °C  40–80%  12:12 h light/dark cycle | Tail  Only female |
| Epilepsy Model – Kindling (teth) | Möller et al., 2018^20^ | Rat | Sprague-Dawley | Female | Macrolon type III cage (Ehret GmbH)  Single-housed  Enviro Dri® | Diet (R/M Haltung, ssniff Spezialdiäten GmbH) and tap water ad libitum | 20–24 °C  45–65%  12:12 h light/dark cycle | -  Females and males |
| Epilepsy Model – Kindling (tele) | Möller et al., 2019^21^ |  |  |  |  |  |  |  |
| Epilepsy Model – Post SE (pilo) | Koska et al., 2019^26^ | Rat | Sprague-Dawley | Female | Macrolon type III cage (Ehret GmbH)  Single-housed  Enviro Dri® | Diet (ssniff Spezialdiäten GmbH) and tap water ad libitum | 22–24 °C  45–65%  12:12 h light/dark cycle | -  Females and males |
|  | Di Liberto et al., 2018^27^ |  |  |  |  |  |  |  |
| Epilepsy Model – Post SE (electrical) | Seiffert et al., 2019^28^ | Rat | Sprague-Dawley | Female | Macrolon type III cage  Single-housed  Enviro Dri® | Diet (R/M Haltung, ssniff Spezialdiäten GmbH) and tap water ad libitum | 20–24 °C  45–65%  12:12 h light/dark cycle | -  Females and males |
|  | van Dijk et al., 2018^29^ |  |  |  |  |  |  |  |
| Hearing Loss Model | Johne et al., 2022^32^ | Rat | Sprague-Dawley | Male | Macrolon Type IV cage  Group-housed  Cardbox, Woodpieces | Standard rodent diet (Altromin) 14-16g per day and tap water ad libitum | 20–24 °C  45–65%  14:10 h light/dark cycle | -  Females and males |
|  | Jelinek et al., 2024^33^ |  |  |  |  |  |  |  |
|  | Stenzel et al., 2025^34^ |  |  |  | Macrolon Type IV cage  Group-housed  - |  |  | -  - |
| Parkinsonism Model | Riedesel et al., 2023^31^ | Rat | Sprague-Dawley | Male | Type IV and Type III (Tecniplast)  Temporal group-housed  Nesting material | 1324 TPF (Altromin) and bottled water ad libitum | 20–24 °C  45–65%  14:10 h light/dark cycle | -  Females and males |
| Glioma Model | Riedesel et al., 2023^31^ | Rat | BDIX | Male | Type IV and Type III (Tecniplast)  Temporal group-housed  Nesting material | 1324 TPF (Altromin) and bottled water ad libitum | 20–24 °C  45–65%  14:10 h light/dark cycle | -  Females and males |
| Glioma Model (endpoint) | Helgers et al., 2020^36^ | Rat | BDIX | Male | Macrolon Type IV cage  Group-housed (n = 2-4)  Enviro Dri® | Diet (1324 TPF, Altromin Spezialfutter GmbH&Co. KG) and tap water ad libitum | 20–24 °C  45–65%  14:10 h light/dark cycle | -  Females and males |
| Dravet Syndrome Model | Reiber et al., 2022^23^ | Mouse | B6(Cg)-*Scn1a*^tm1.1Dsf^/J x 129S1/Sv-Hprt^tm1(CAG-cre)Mnn^/J | Female and male | Macrolon type III cage (Zoonlab) and Phenotyper (Noldus)  Pair-housed  Nestlets, mouse house | Diet (ssniff Spezialdiäten GmbH) and tap water ad libitum | 22–24 °C  45–60%  12:12 h light/dark cycle | Tail  Only females |
| *Gria1* KO Model | Reiber et al., 2022^24^ | Mouse | B6N.129-*Gria1* ^tm1Rsp^ /J | Female and male | Macrolon type III cage (Zoonlab) and Phenotyper (Noldus)  Pair-housed  Nestlets, mouse house | Diet (ssniff Spezialdiäten GmbH) and tap water ad libitum | 22–24 °C  45–60%  12:12 h light/dark cycle | Tail  Only females |
| DAT KO Model | Mallien et al., 2022^15^ | Rat | Wistar-Han  DAT^-/-^ and DAT^+/+-^ | Male | Type IV cage, 59.8 x 38 x 20 cm (Tecniplast)  Pair-housed  Tunnel, gnawing stick, paper, sizzle nest | Diet (LASQC diet Rod16 HiHyg, Altromin) and tap water ad libitum | 21–23 °C  40–50%  12:12 h light/dark cycle | -  Only females |
| Restraint Stress Model | Häger et al., 2018^10^ | Mouse | C57BL/6J | Female | Macrolon cage, 360 cm^2^  Single-housed  Running wheels | Standard rodent food (1324, Altromin) and autoclaved water ad libitum | 21–23 °C  50–60%  14:10 h light/dark cycle | Tail  Only females |
| Forced Swim Test | Becker et al., 2023^17^ | Rat | Wistar-Han | Female and male | Type IV cage (Tecniplast)  Pair-housed  Gnawing stick, paper, sizzle nest | Diet (LASQC diet Rod16 HiHyg, Altromin) and tap water ad libitum | 21–24 °C  40–50%  12:12 h light/dark cycle | -  Only females |
| Biopotential Recording Techniques | Aulehner et al., 2022^30^ | Rat | Sprague-Dawley | Female | Macrolon type III cage (Ehret GmbH)  Single-housed  Enviro Dri® | Pelleted diet (ssniff Spezialdiäten GmbH) and tap water ad libitum | 22–24 °C  45–65%  12:12 h light/dark cycle | -  Females and males |

The symbol (-) indicates that the data could not be retrieved or do not apply. hp, hippocampus; bla, basolateral amygdala; SE, Status epilepticus; IHK, intrahippocampal kainate; teth, tethered; tele, telemetry; pilo, pilocarpine; DAT, Dopamine Transporter.

Table S9. Information about the anesthesia and analgesia protocols used in the studies included in the surgical interventions category.

| Study ID | Anesthesia | Analgesia | |
| --- | --- | --- | --- |
|  |  | Pre- or intra-surgery* | Post-surgery |
| Intracerebral Electrode Implantation | Isoflurane (4% induction and 1.8-2% maintenance) | Carprofen 25 mg/kg via drinking water for 20-24 h and  Buprenorphine 1.2 mg/kg s.c. and/or  Bupivacaine 0.25% (8 mg/kg) + epinephrine 0.00025% s.c. | Carprofen 25 mg/kg via drinking water for 96 h |
| Intraperitoneal Transmitter Implantation | Isoflurane (5% induction and 1.5-2.5% maintenance) | Lidocaine (25 mg/g) + Prilocaine (25 mg/g) epidermal and  Metamizole 200 mg/kg s.c. or  Carprofen 5 mg/kg s.c. | Metamizole 200 mg/kg via drinking water for 72 h or  Carprofen 2.5 mg/kg s.c. BID for 72 h |
| Subarachnoid Hemorrhage (SAH) Model | Isoflurane (4% induction and 2% maintenance) | Fentanyl 0.015 mg/kg s.c. and  Buprenorphine 0.03 mg/kg s.c. and  Ropivacaine hydrochloride (2 mg/ml) s.c. | Buprenorphine 0.03 mg/kg s.c. BID for 72 h |
| Partial Hepatectomy (PH) | Isoflurane (5% induction and 2% maintenance) | Metamizole 100 mg/kg s.c. | Metamizole 400 mg/kg via drinking water for 72 h |
| Pancreatic Cancer Model | Isoflurane (4% induction and 1.2-2% maintenance) | Carprofen 5 mg/kg s.c. | Metamizole (1250 mg/L) via drinking water for 37 days |
| Cerebellar Cognitive Affective Syndrome Model | Chloral hydrate 360 mg/kg i.p. | Carprofen 5 mg/kg s.c. and  Xylocaine (2%) s.c. | Carprofen 2.5 mg/kg s.c. QD for 48 h |
| Epilepsy Model – Post SE (IHK) | Chloral hydrate 500 mg/kg i.p. | Meloxicam 5 mg/kg s.c. and  Bupivacaine 0.25% (8 mg/kg) + epinephrine 0.00025% s.c. and  Bupivacaine 0.25% (8 mg/kg) s.c. | Meloxicam 5 mg/kg s.c. QD for 24 h |
| Parkinsonism Model | Chloral hydrate 360 mg/kg i.p. | Carprofen 5 mg/kg s.c. and  Xylocaine 1% s.c. | Carprofen 5 mg/kg s.c. QD for 48 h |
| Glioma Model | Chloral hydrate 360 mg/kg i.p. | Carprofen 5 mg/kg s.c. and  Xylocaine 1% s.c. | Carprofen 5 mg/kg s.c. QD for 48 h |

* Compounds were administered as a single dose (except when the dosing scheme is specified) either before or during the surgical intervention.

h, hours; s.c., subcutaneously; BID, twice daily; i.p., intraperitoneally; QD, once daily.

Table S10. Details on the inclusion and exclusion of the different studies per readout parameter.

| **Readout parameter** | **Number of datasets** | | | **Reasons for exclusion** |
| --- | --- | --- | --- | --- |
|  | **Identified** | **Included** | **Excluded** |  |
| Clinical Score | 36 | 20 | 16 | Unpublished data (9)  Data will be used on other projects (3)  Different species (1)  No access to “activity-related” scores (1)  No reference data for comparison (2) |
| Neuroscore | 15 | 13 | 2 | Unpublished data (2) |
| Open field | 30 | 18 | 12 | Unpublished data (6)  Data not shared on time (3)  Different species (2)  No reference data for comparison (1) |
| Voluntary wheel running | 13 | 6 | 7 | Unpublished data (5)  Data will be used on other projects (4)  No reference data for comparison (1) |
| Home-cage-based video monitoring | 3 | 3 | 0 |  |
| Telemetry | 24 | 9 | 15 | Unpublished data (12)  Different species (2)  Sample size too small (1) |

Table S11. Effect sizes and confidence intervals for the studies included in the “surgical interventions” category.

| **Model ID** | **Readout parameter** | **Experimental/ Intervention group** | **Reference data** | **Experimental day(s)** | **Sample size Exp/Int group** | **Sample size Reference data** | **Effect size** | **CI lower** | **CI higher** |
| --- | --- | --- | --- | --- | --- | --- | --- | --- | --- |
| Intracerebral Electrode Implantation | Clinical Score | Surgery + N (Female) | Naïve | 1 | 8 | 8 | 1.05 | 0.07 | 2.03 |
| Intracerebral Electrode Implantation | Clinical Score | Surgery + NL (Female) | Naïve | 1 | 8 | 8 | 1.05 | 0.07 | 2.03 |
| Intracerebral Electrode Implantation | Clinical Score | Surgery + NL (Female) | Naïve | 2 | 8 | 8 | 0.72 | -0.26 | 1.70 |
| Intracerebral Electrode Implantation | Clinical Score | Surgery + NL (Female) | Naïve | 3 | 8 | 8 | 0.72 | -0.26 | 1.70 |
| Intracerebral Electrode Implantation | Clinical Score | Surgery + NLO (Female) | Naïve | 1 | 8 | 8 | 0.72 | -0.26 | 1.70 |
| Intracerebral Electrode Implantation | Clinical Score | Surgery + NO (Female) | Naïve | 1 | 8 | 8 | 1.32 | 0.34 | 2.30 |
| Intracerebral Electrode Implantation | Clinical Score | Surgery + NO (Female) | Naïve | 2 | 8 | 8 | 0.72 | -0.26 | 1.70 |
| Intracerebral Electrode Implantation | Clinical Score | Surgery + N (Male) | Naïve | 1 | 8 | 8 | 1.05 | 0.07 | 2.03 |
| Intracerebral Electrode Implantation | Clinical Score | Surgery + N (Male) | Naïve | 2 | 8 | 8 | 1.32 | 0.34 | 2.30 |
| Intracerebral Electrode Implantation | Clinical Score | Surgery + N (Male) | Naïve | 3 | 8 | 8 | 1.05 | 0.07 | 2.03 |
| Intracerebral Electrode Implantation | Clinical Score | Surgery + NL (Male) | Naïve | 1 | 8 | 8 | 1.05 | 0.07 | 2.03 |
| Intracerebral Electrode Implantation | Clinical Score | Surgery + NL (Male) | Naïve | 2 | 8 | 8 | 1.05 | 0.07 | 2.03 |
| Intracerebral Electrode Implantation | Clinical Score | Surgery + NLO (Male) | Naïve | 1 | 8 | 8 | 0.72 | -0.26 | 1.70 |
| Intracerebral Electrode Implantation | Clinical Score | Surgery + NLO (Male) | Naïve | 2 | 8 | 8 | 0.72 | -0.26 | 1.70 |
| Intracerebral Electrode Implantation | Clinical Score | Surgery + NO (Male) | Naïve | 1 | 8 | 8 | 1.05 | 0.07 | 2.03 |
| Intracerebral Electrode Implantation | Clinical Score | Surgery + N (Female) | Sham (drug-control) | 1 | 8 | 8 | 1.05 | 0.07 | 2.03 |
| Intracerebral Electrode Implantation | Clinical Score | Surgery + N (Male) | Sham (drug-control) | 1 | 8 | 8 | 1.05 | 0.07 | 2.03 |
| Intracerebral Electrode Implantation | Clinical Score | Surgery + N (Male) | Sham (drug-control) | 2 | 8 | 8 | 1.32 | 0.34 | 2.30 |
| Intracerebral Electrode Implantation | Clinical Score | Surgery + N (Male) | Sham (drug-control) | 3 | 8 | 8 | 1.05 | 0.07 | 2.03 |
| Intracerebral Electrode Implantation | Clinical Score | Surgery + NL (Female) | Sham (drug-control) | 1 | 8 | 8 | 1.05 | 0.07 | 2.03 |
| Intracerebral Electrode Implantation | Clinical Score | Surgery + NL (Female) | Sham (drug-control) | 2 | 8 | 8 | 0.72 | -0.26 | 1.70 |
| Intracerebral Electrode Implantation | Clinical Score | Surgery + NL (Female) | Sham (drug-control) | 3 | 8 | 8 | 0.72 | -0.26 | 1.70 |
| Intracerebral Electrode Implantation | Clinical Score | Surgery + NL (Male) | Sham (drug-control) | 1 | 8 | 8 | 1.05 | 0.07 | 2.03 |
| Intracerebral Electrode Implantation | Clinical Score | Surgery + NL (Male) | Sham (drug-control) | 2 | 8 | 8 | 1.05 | 0.07 | 2.03 |
| Intracerebral Electrode Implantation | Clinical Score | Surgery + NO (Female) | Sham (drug-control) | 1 | 8 | 8 | 1.32 | 0.34 | 2.30 |
| Intracerebral Electrode Implantation | Clinical Score | Surgery + NO (Female) | Sham (drug-control) | 2 | 8 | 8 | 0.72 | -0.26 | 1.70 |
| Intracerebral Electrode Implantation | Clinical Score | Surgery + NO (Male) | Sham (drug-control) | 1 | 8 | 8 | 1.05 | 0.07 | 2.03 |
| Intracerebral Electrode Implantation | Clinical Score | Surgery + NLO (Female) | Sham (drug-control) | 1 | 8 | 8 | 0.72 | -0.26 | 1.70 |
| Intracerebral Electrode Implantation | Clinical Score | Surgery + NLO (Male) | Sham (drug-control) | 1 | 8 | 8 | 0.72 | -0.26 | 1.70 |
| Intracerebral Electrode Implantation | Clinical Score | Surgery + NLO (Male) | Sham (drug-control) | 2 | 8 | 8 | 0.72 | -0.26 | 1.70 |
| Intracerebral Electrode Implantation | Clinical Score | Surgery + N (Female) | Baseline | 1 | 8 | 8 | 1.05 | 0.07 | 2.03 |
| Intracerebral Electrode Implantation | Clinical Score | Surgery + NL (Female) | Baseline | 1 | 8 | 8 | 1.05 | 0.07 | 2.03 |
| Intracerebral Electrode Implantation | Clinical Score | Surgery + NL (Female) | Baseline | 2 | 8 | 8 | 0.72 | -0.26 | 1.70 |
| Intracerebral Electrode Implantation | Clinical Score | Surgery + NL (Female) | Baseline | 3 | 8 | 8 | 0.72 | -0.26 | 1.70 |
| Intracerebral Electrode Implantation | Clinical Score | Surgery + NLO (Female) | Baseline | 1 | 8 | 8 | 0.72 | -0.26 | 1.70 |
| Intracerebral Electrode Implantation | Clinical Score | Surgery + NO (Female) | Baseline | 1 | 8 | 8 | 1.32 | 0.34 | 2.30 |
| Intracerebral Electrode Implantation | Clinical Score | Surgery + NO (Female) | Baseline | 2 | 8 | 8 | 0.72 | -0.26 | 1.70 |
| Intracerebral Electrode Implantation | Clinical Score | Surgery + N (Male) | Baseline | 1 | 8 | 8 | 1.05 | 0.07 | 2.03 |
| Intracerebral Electrode Implantation | Clinical Score | Surgery + N (Male) | Baseline | 2 | 8 | 8 | 1.32 | 0.34 | 2.30 |
| Intracerebral Electrode Implantation | Clinical Score | Surgery + N (Male) | Baseline | 3 | 8 | 8 | 1.05 | 0.07 | 2.03 |
| Intracerebral Electrode Implantation | Clinical Score | Surgery + NL (Male) | Baseline | 1 | 8 | 8 | 1.05 | 0.07 | 2.03 |
| Intracerebral Electrode Implantation | Clinical Score | Surgery + NL (Male) | Baseline | 2 | 8 | 8 | 1.05 | 0.07 | 2.03 |
| Intracerebral Electrode Implantation | Clinical Score | Surgery + NLO (Male) | Baseline | 1 | 8 | 8 | 0.72 | -0.26 | 1.70 |
| Intracerebral Electrode Implantation | Clinical Score | Surgery + NLO (Male) | Baseline | 2 | 8 | 8 | 0.72 | -0.26 | 1.70 |
| Intracerebral Electrode Implantation | Clinical Score | Surgery + NO (Male) | Baseline | 1 | 8 | 8 | 1.05 | 0.07 | 2.03 |
| Intracerebral Electrode Implantation | Neuroscore | Sham + N (Female) | Naïve | 0 | 8 | 8 | 0.72 | -0.26 | 1.70 |
| Intracerebral Electrode Implantation | Neuroscore | Sham + N (Female) | Naïve | 1 | 8 | 8 | 0.72 | -0.26 | 1.70 |
| Intracerebral Electrode Implantation | Neuroscore | Sham + NLO (Female) | Naïve | 0 | 8 | 8 | 1.82 | 0.84 | 2.80 |
| Intracerebral Electrode Implantation | Neuroscore | Sham + NO (Female) | Naïve | 0 | 8 | 8 | 1.32 | 0.34 | 2.30 |
| Intracerebral Electrode Implantation | Neuroscore | Surgery + N (Female) | Naïve | 0 | 8 | 8 | 1.32 | 0.34 | 2.30 |
| Intracerebral Electrode Implantation | Neuroscore | Surgery + N (Female) | Naïve | 1 | 8 | 8 | 1.32 | 0.34 | 2.30 |
| Intracerebral Electrode Implantation | Neuroscore | Surgery + N (Female) | Naïve | 3 | 8 | 8 | 1.32 | 0.34 | 2.30 |
| Intracerebral Electrode Implantation | Neuroscore | Surgery + NL (Female) | Naïve | 0 | 8 | 8 | 1.05 | 0.07 | 2.03 |
| Intracerebral Electrode Implantation | Neuroscore | Surgery + NL (Female) | Naïve | 1 | 8 | 8 | 1.05 | 0.07 | 2.03 |
| Intracerebral Electrode Implantation | Neuroscore | Surgery + NL (Female) | Naïve | 2 | 8 | 8 | 0.72 | -0.26 | 1.70 |
| Intracerebral Electrode Implantation | Neuroscore | Surgery + NLO (Female) | Naïve | 0 | 8 | 8 | 0.72 | -0.26 | 1.70 |
| Intracerebral Electrode Implantation | Neuroscore | Surgery + NLO (Female) | Naïve | 1 | 8 | 8 | 1.05 | 0.07 | 2.03 |
| Intracerebral Electrode Implantation | Neuroscore | Surgery + NO (Female) | Naïve | 0 | 8 | 8 | 1.05 | 0.07 | 2.03 |
| Intracerebral Electrode Implantation | Neuroscore | Surgery + NO (Female) | Naïve | 1 | 8 | 8 | 1.05 | 0.07 | 2.03 |
| Intracerebral Electrode Implantation | Neuroscore | Surgery + NO (Female) | Naïve | 2 | 8 | 8 | 0.72 | -0.26 | 1.70 |
| Intracerebral Electrode Implantation | Neuroscore | Sham + NLO (Male) | Naïve | 0 | 8 | 8 | 1.32 | 0.34 | 2.30 |
| Intracerebral Electrode Implantation | Neuroscore | Sham + NO (Male) | Naïve | 0 | 8 | 8 | 1.57 | 0.59 | 2.55 |
| Intracerebral Electrode Implantation | Neuroscore | Surgery + N (Male) | Naïve | -4 | 8 | 8 | 0.72 | -0.26 | 1.70 |
| Intracerebral Electrode Implantation | Neuroscore | Surgery + N (Male) | Naïve | 0 | 8 | 8 | 0.72 | -0.26 | 1.70 |
| Intracerebral Electrode Implantation | Neuroscore | Surgery + N (Male) | Naïve | 1 | 8 | 8 | 1.32 | 0.34 | 2.30 |
| Intracerebral Electrode Implantation | Neuroscore | Surgery + N (Male) | Naïve | 2 | 8 | 8 | 1.05 | 0.07 | 2.03 |
| Intracerebral Electrode Implantation | Neuroscore | Surgery + N (Male) | Naïve | 3 | 8 | 8 | 0.72 | -0.26 | 1.70 |
| Intracerebral Electrode Implantation | Neuroscore | Surgery + N (Male) | Naïve | 4 | 7 | 8 | 0.78 | -0.24 | 1.79 |
| Intracerebral Electrode Implantation | Neuroscore | Surgery + NL (Male) | Naïve | 1 | 8 | 8 | 1.05 | 0.07 | 2.03 |
| Intracerebral Electrode Implantation | Neuroscore | Surgery + NL (Male) | Naïve | 2 | 8 | 8 | 0.72 | -0.26 | 1.70 |
| Intracerebral Electrode Implantation | Neuroscore | Surgery + NLO (Male) | Naïve | -4 | 8 | 8 | 0.72 | -0.26 | 1.70 |
| Intracerebral Electrode Implantation | Neuroscore | Surgery + NLO (Male) | Naïve | 0 | 8 | 8 | 0.72 | -0.26 | 1.70 |
| Intracerebral Electrode Implantation | Neuroscore | Surgery + NLO (Male) | Naïve | 2 | 8 | 8 | 0.72 | -0.26 | 1.70 |
| Intracerebral Electrode Implantation | Neuroscore | Surgery + NO (Male) | Naïve | 0 | 8 | 8 | 1.05 | 0.07 | 2.03 |
| Intracerebral Electrode Implantation | Neuroscore | Surgery + NO (Male) | Naïve | 1 | 8 | 8 | 1.05 | 0.07 | 2.03 |
| Intracerebral Electrode Implantation | Neuroscore | Surgery + NO (Male) | Naïve | 2 | 8 | 8 | 0.72 | -0.26 | 1.70 |
| Intracerebral Electrode Implantation | Neuroscore | Surgery + N (Female) | Sham (drug-control) | 0 | 8 | 8 | 0.60 | -0.38 | 1.58 |
| Intracerebral Electrode Implantation | Neuroscore | Surgery + N (Female) | Sham (drug-control) | 1 | 8 | 8 | 0.60 | -0.38 | 1.58 |
| Intracerebral Electrode Implantation | Neuroscore | Surgery + N (Female) | Sham (drug-control) | 3 | 8 | 8 | 1.32 | 0.34 | 2.30 |
| Intracerebral Electrode Implantation | Neuroscore | Surgery + N (Male) | Sham (drug-control) | -4 | 8 | 8 | 0.72 | -0.26 | 1.70 |
| Intracerebral Electrode Implantation | Neuroscore | Surgery + N (Male) | Sham (drug-control) | 0 | 8 | 8 | 0.72 | -0.26 | 1.70 |
| Intracerebral Electrode Implantation | Neuroscore | Surgery + N (Male) | Sham (drug-control) | 1 | 8 | 8 | 1.32 | 0.34 | 2.30 |
| Intracerebral Electrode Implantation | Neuroscore | Surgery + N (Male) | Sham (drug-control) | 2 | 8 | 8 | 1.05 | 0.07 | 2.03 |
| Intracerebral Electrode Implantation | Neuroscore | Surgery + N (Male) | Sham (drug-control) | 3 | 8 | 8 | 0.72 | -0.26 | 1.70 |
| Intracerebral Electrode Implantation | Neuroscore | Surgery + N (Male) | Sham (drug-control) | 4 | 7 | 8 | 0.78 | -0.24 | 1.79 |
| Intracerebral Electrode Implantation | Neuroscore | Surgery + NL (Female) | Sham (drug-control) | 0 | 8 | 8 | 1.05 | 0.07 | 2.03 |
| Intracerebral Electrode Implantation | Neuroscore | Surgery + NL (Female) | Sham (drug-control) | 1 | 8 | 8 | 1.05 | 0.07 | 2.03 |
| Intracerebral Electrode Implantation | Neuroscore | Surgery + NL (Female) | Sham (drug-control) | 2 | 8 | 8 | 0.72 | -0.26 | 1.70 |
| Intracerebral Electrode Implantation | Neuroscore | Surgery + NL (Male) | Sham (drug-control) | 1 | 8 | 8 | 1.05 | 0.07 | 2.03 |
| Intracerebral Electrode Implantation | Neuroscore | Surgery + NL (Male) | Sham (drug-control) | 2 | 8 | 8 | 0.72 | -0.26 | 1.70 |
| Intracerebral Electrode Implantation | Neuroscore | Surgery + NO (Female) | Sham (drug-control) | 0 | 8 | 8 | -0.27 | -1.25 | 0.71 |
| Intracerebral Electrode Implantation | Neuroscore | Surgery + NO (Female) | Sham (drug-control) | 1 | 8 | 8 | 1.05 | 0.07 | 2.03 |
| Intracerebral Electrode Implantation | Neuroscore | Surgery + NO (Female) | Sham (drug-control) | 2 | 8 | 8 | 0.72 | -0.26 | 1.70 |
| Intracerebral Electrode Implantation | Neuroscore | Surgery + NO (Male) | Sham (drug-control) | 0 | 8 | 8 | -0.52 | -1.50 | 0.46 |
| Intracerebral Electrode Implantation | Neuroscore | Surgery + NO (Male) | Sham (drug-control) | 1 | 8 | 8 | 1.05 | 0.07 | 2.03 |
| Intracerebral Electrode Implantation | Neuroscore | Surgery + NO (Male) | Sham (drug-control) | 2 | 8 | 8 | 0.72 | -0.26 | 1.70 |
| Intracerebral Electrode Implantation | Neuroscore | Surgery + NLO (Female) | Sham (drug-control) | 0 | 8 | 8 | -1.10 | -2.08 | -0.12 |
| Intracerebral Electrode Implantation | Neuroscore | Surgery + NLO (Female) | Sham (drug-control) | 1 | 8 | 8 | 1.05 | 0.07 | 2.03 |
| Intracerebral Electrode Implantation | Neuroscore | Surgery + NLO (Male) | Sham (drug-control) | -4 | 8 | 8 | 0.72 | -0.26 | 1.70 |
| Intracerebral Electrode Implantation | Neuroscore | Surgery + NLO (Male) | Sham (drug-control) | 0 | 8 | 8 | -0.60 | -1.58 | 0.38 |
| Intracerebral Electrode Implantation | Neuroscore | Surgery + NLO (Male) | Sham (drug-control) | 2 | 8 | 8 | 0.72 | -0.26 | 1.70 |
| Intracerebral Electrode Implantation | Neuroscore | Sham + N (Female) | Baseline | 0 | 8 | 8 | 0.72 | -0.26 | 1.70 |
| Intracerebral Electrode Implantation | Neuroscore | Sham + N (Female) | Baseline | 1 | 8 | 8 | 0.72 | -0.26 | 1.70 |
| Intracerebral Electrode Implantation | Neuroscore | Sham + NLO (Female) | Baseline | 0 | 8 | 8 | 1.82 | 0.84 | 2.80 |
| Intracerebral Electrode Implantation | Neuroscore | Sham + NO (Female) | Baseline | 0 | 8 | 8 | 1.32 | 0.34 | 2.30 |
| Intracerebral Electrode Implantation | Neuroscore | Surgery + N (Female) | Baseline | 0 | 8 | 8 | 1.32 | 0.34 | 2.30 |
| Intracerebral Electrode Implantation | Neuroscore | Surgery + N (Female) | Baseline | 1 | 8 | 8 | 1.32 | 0.34 | 2.30 |
| Intracerebral Electrode Implantation | Neuroscore | Surgery + N (Female) | Baseline | 3 | 8 | 8 | 1.32 | 0.34 | 2.30 |
| Intracerebral Electrode Implantation | Neuroscore | Surgery + NL (Female) | Baseline | 0 | 8 | 8 | 1.05 | 0.07 | 2.03 |
| Intracerebral Electrode Implantation | Neuroscore | Surgery + NL (Female) | Baseline | 1 | 8 | 8 | 1.05 | 0.07 | 2.03 |
| Intracerebral Electrode Implantation | Neuroscore | Surgery + NL (Female) | Baseline | 2 | 8 | 8 | 0.72 | -0.26 | 1.70 |
| Intracerebral Electrode Implantation | Neuroscore | Surgery + NLO (Female) | Baseline | 0 | 8 | 8 | 0.72 | -0.26 | 1.70 |
| Intracerebral Electrode Implantation | Neuroscore | Surgery + NLO (Female) | Baseline | 1 | 8 | 8 | 1.05 | 0.07 | 2.03 |
| Intracerebral Electrode Implantation | Neuroscore | Surgery + NO (Female) | Baseline | 0 | 8 | 8 | 1.05 | 0.07 | 2.03 |
| Intracerebral Electrode Implantation | Neuroscore | Surgery + NO (Female) | Baseline | 1 | 8 | 8 | 1.05 | 0.07 | 2.03 |
| Intracerebral Electrode Implantation | Neuroscore | Surgery + NO (Female) | Baseline | 2 | 8 | 8 | 0.72 | -0.26 | 1.70 |
| Intracerebral Electrode Implantation | Neuroscore | Sham + NLO (Male) | Baseline | 0 | 8 | 8 | 1.32 | 0.34 | 2.30 |
| Intracerebral Electrode Implantation | Neuroscore | Sham + NO (Male) | Baseline | 0 | 8 | 8 | 1.57 | 0.59 | 2.55 |
| Intracerebral Electrode Implantation | Neuroscore | Surgery + N (Male) | Baseline | 0 | 8 | 8 | 0.00 | -0.98 | 0.98 |
| Intracerebral Electrode Implantation | Neuroscore | Surgery + N (Male) | Baseline | 1 | 8 | 8 | 0.60 | -0.38 | 1.58 |
| Intracerebral Electrode Implantation | Neuroscore | Surgery + N (Male) | Baseline | 2 | 8 | 8 | 0.32 | -0.66 | 1.30 |
| Intracerebral Electrode Implantation | Neuroscore | Surgery + N (Male) | Baseline | 3 | 8 | 8 | 0.00 | -0.98 | 0.98 |
| Intracerebral Electrode Implantation | Neuroscore | Surgery + N (Male) | Baseline | 4 | 7 | 8 | 0.05 | -0.96 | 1.07 |
| Intracerebral Electrode Implantation | Neuroscore | Surgery + NL (Male) | Baseline | 1 | 8 | 8 | 1.05 | 0.07 | 2.03 |
| Intracerebral Electrode Implantation | Neuroscore | Surgery + NL (Male) | Baseline | 2 | 8 | 8 | 0.72 | -0.26 | 1.70 |
| Intracerebral Electrode Implantation | Neuroscore | Surgery + NLO (Male) | Baseline | 0 | 8 | 8 | 0.00 | -0.98 | 0.98 |
| Intracerebral Electrode Implantation | Neuroscore | Surgery + NLO (Male) | Baseline | 2 | 8 | 8 | 0.00 | -0.98 | 0.98 |
| Intracerebral Electrode Implantation | Neuroscore | Surgery + NO (Male) | Baseline | 0 | 8 | 8 | 1.05 | 0.07 | 2.03 |
| Intracerebral Electrode Implantation | Neuroscore | Surgery + NO (Male) | Baseline | 1 | 8 | 8 | 1.05 | 0.07 | 2.03 |
| Intracerebral Electrode Implantation | Neuroscore | Surgery + NO (Male) | Baseline | 2 | 8 | 8 | 0.72 | -0.26 | 1.70 |
| Intracerebral Electrode Implantation | VWR (light phase) | Sham + N (Female) | Naïve | 2 | 8 | 8 | 0.51 | -0.44 | 1.45 |
| Intracerebral Electrode Implantation | VWR (light phase) | Sham + N (Female) | Naïve | 3 | 8 | 8 | -0.54 | -1.48 | 0.42 |
| Intracerebral Electrode Implantation | VWR (light phase) | Sham + NL (Female) | Naïve | 2 | 8 | 8 | 0.91 | -0.09 | 1.88 |
| Intracerebral Electrode Implantation | VWR (light phase) | Sham + NL (Female) | Naïve | 3 | 8 | 8 | 0.45 | -0.50 | 1.39 |
| Intracerebral Electrode Implantation | VWR (light phase) | Sham + NLO (Female) | Naïve | 2 | 8 | 8 | 0.72 | -0.25 | 1.68 |
| Intracerebral Electrode Implantation | VWR (light phase) | Sham + NLO (Female) | Naïve | 3 | 8 | 8 | 0.09 | -0.84 | 1.01 |
| Intracerebral Electrode Implantation | VWR (light phase) | Sham + NO (Female) | Naïve | 2 | 8 | 8 | 0.48 | -0.47 | 1.41 |
| Intracerebral Electrode Implantation | VWR (light phase) | Sham + NO (Female) | Naïve | 3 | 8 | 8 | 0.27 | -0.67 | 1.19 |
| Intracerebral Electrode Implantation | VWR (light phase) | Surgery + N (Female) | Naïve | 2 | 8 | 8 | 0.77 | -0.21 | 1.73 |
| Intracerebral Electrode Implantation | VWR (light phase) | Surgery + N (Female) | Naïve | 3 | 8 | 8 | -0.17 | -1.09 | 0.76 |
| Intracerebral Electrode Implantation | VWR (light phase) | Surgery + NL (Female) | Naïve | 2 | 8 | 8 | 0.81 | -0.17 | 1.77 |
| Intracerebral Electrode Implantation | VWR (light phase) | Surgery + NL (Female) | Naïve | 3 | 8 | 8 | 0.28 | -0.66 | 1.20 |
| Intracerebral Electrode Implantation | VWR (light phase) | Surgery + NLO (Female) | Naïve | 2 | 8 | 8 | 0.64 | -0.32 | 1.59 |
| Intracerebral Electrode Implantation | VWR (light phase) | Surgery + NLO (Female) | Naïve | 3 | 8 | 8 | 0.62 | -0.34 | 1.57 |
| Intracerebral Electrode Implantation | VWR (light phase) | Surgery + NO (Female) | Naïve | 2 | 8 | 8 | -0.32 | -1.25 | 0.62 |
| Intracerebral Electrode Implantation | VWR (light phase) | Surgery + NO (Female) | Naïve | 3 | 8 | 8 | -0.22 | -1.15 | 0.71 |
| Intracerebral Electrode Implantation | VWR (light phase) | Sham + N (Male) | Naïve | 2 | 8 | 8 | 0.51 | -0.45 | 1.44 |
| Intracerebral Electrode Implantation | VWR (light phase) | Sham + N (Male) | Naïve | 3 | 8 | 8 | 0.27 | -0.67 | 1.19 |
| Intracerebral Electrode Implantation | VWR (light phase) | Sham + NL (Male) | Naïve | 2 | 8 | 8 | 0.86 | -0.13 | 1.82 |
| Intracerebral Electrode Implantation | VWR (light phase) | Sham + NL (Male) | Naïve | 3 | 8 | 8 | 0.59 | -0.37 | 1.53 |
| Intracerebral Electrode Implantation | VWR (light phase) | Sham + NLO (Male) | Naïve | 2 | 8 | 8 | 0.61 | -0.36 | 1.55 |
| Intracerebral Electrode Implantation | VWR (light phase) | Sham + NLO (Male) | Naïve | 3 | 8 | 8 | -0.45 | -1.38 | 0.50 |
| Intracerebral Electrode Implantation | VWR (light phase) | Sham + NO (Male) | Naïve | 2 | 8 | 8 | 0.38 | -0.57 | 1.31 |
| Intracerebral Electrode Implantation | VWR (light phase) | Sham + NO (Male) | Naïve | 3 | 8 | 8 | 0.33 | -0.61 | 1.26 |
| Intracerebral Electrode Implantation | VWR (light phase) | Surgery + N (Male) | Naïve | 2 | 8 | 8 | -0.34 | -1.27 | 0.60 |
| Intracerebral Electrode Implantation | VWR (light phase) | Surgery + N (Male) | Naïve | 3 | 8 | 8 | 0.22 | -0.71 | 1.15 |
| Intracerebral Electrode Implantation | VWR (light phase) | Surgery + NL (Male) | Naïve | 2 | 8 | 8 | 0.88 | -0.11 | 1.85 |
| Intracerebral Electrode Implantation | VWR (light phase) | Surgery + NL (Male) | Naïve | 3 | 8 | 8 | 0.43 | -0.52 | 1.36 |
| Intracerebral Electrode Implantation | VWR (light phase) | Surgery + NLO (Male) | Naïve | 2 | 8 | 8 | 0.32 | -0.62 | 1.24 |
| Intracerebral Electrode Implantation | VWR (light phase) | Surgery + NLO (Male) | Naïve | 3 | 8 | 8 | -0.10 | -1.02 | 0.83 |
| Intracerebral Electrode Implantation | VWR (light phase) | Surgery + NO (Male) | Naïve | 2 | 8 | 8 | -0.23 | -1.16 | 0.71 |
| Intracerebral Electrode Implantation | VWR (light phase) | Surgery + NO (Male) | Naïve | 3 | 8 | 8 | 0.09 | -0.84 | 1.01 |
| Intracerebral Electrode Implantation | VWR (light phase) | Surgery + N (Female) | Sham (drug-control) | 2 | 8 | 8 | -0.26 | -1.19 | 0.67 |
| Intracerebral Electrode Implantation | VWR (light phase) | Surgery + N (Female) | Sham (drug-control) | 3 | 8 | 8 | 0.42 | -0.52 | 1.36 |
| Intracerebral Electrode Implantation | VWR (light phase) | Surgery + N (Male) | Sham (drug-control) | 2 | 8 | 8 | -0.69 | -1.63 | 0.29 |
| Intracerebral Electrode Implantation | VWR (light phase) | Surgery + N (Male) | Sham (drug-control) | 3 | 8 | 8 | -0.02 | -0.94 | 0.91 |
| Intracerebral Electrode Implantation | VWR (light phase) | Surgery + NL (Female) | Sham (drug-control) | 2 | 8 | 8 | -0.47 | -1.41 | 0.48 |
| Intracerebral Electrode Implantation | VWR (light phase) | Surgery + NL (Female) | Sham (drug-control) | 3 | 8 | 8 | -0.31 | -1.24 | 0.62 |
| Intracerebral Electrode Implantation | VWR (light phase) | Surgery + NL (Male) | Sham (drug-control) | 2 | 8 | 8 | -0.45 | -1.38 | 0.50 |
| Intracerebral Electrode Implantation | VWR (light phase) | Surgery + NL (Male) | Sham (drug-control) | 3 | 8 | 8 | -0.24 | -1.17 | 0.69 |
| Intracerebral Electrode Implantation | VWR (light phase) | Surgery + NO (Female) | Sham (drug-control) | 2 | 8 | 8 | -0.79 | -1.75 | 0.19 |
| Intracerebral Electrode Implantation | VWR (light phase) | Surgery + NO (Female) | Sham (drug-control) | 3 | 8 | 8 | -0.39 | -1.32 | 0.56 |
| Intracerebral Electrode Implantation | VWR (light phase) | Surgery + NO (Male) | Sham (drug-control) | 2 | 8 | 8 | -0.55 | -1.49 | 0.41 |
| Intracerebral Electrode Implantation | VWR (light phase) | Surgery + NO (Male) | Sham (drug-control) | 3 | 8 | 8 | -0.25 | -1.17 | 0.69 |
| Intracerebral Electrode Implantation | VWR (light phase) | Surgery + NLO (Female) | Sham (drug-control) | 2 | 8 | 8 | -0.61 | -1.55 | 0.36 |
| Intracerebral Electrode Implantation | VWR (light phase) | Surgery + NLO (Female) | Sham (drug-control) | 3 | 8 | 8 | 0.62 | -0.35 | 1.56 |
| Intracerebral Electrode Implantation | VWR (light phase) | Surgery + NLO (Male) | Sham (drug-control) | 2 | 8 | 8 | -0.38 | -1.31 | 0.57 |
| Intracerebral Electrode Implantation | VWR (light phase) | Surgery + NLO (Male) | Sham (drug-control) | 3 | 8 | 8 | 0.40 | -0.55 | 1.33 |
| Intracerebral Electrode Implantation | VWR (dark phase) | Sham + N (Female) | Naïve | 1 | 8 | 8 | 0.55 | -0.40 | 1.49 |
| Intracerebral Electrode Implantation | VWR (dark phase) | Sham + N (Female) | Naïve | 2 | 8 | 8 | 0.15 | -0.78 | 1.07 |
| Intracerebral Electrode Implantation | VWR (dark phase) | Sham + N (Female) | Naïve | 3 | 8 | 8 | 0.15 | -0.78 | 1.08 |
| Intracerebral Electrode Implantation | VWR (dark phase) | Sham + NL (Female) | Naïve | 1 | 8 | 8 | 0.44 | -0.51 | 1.37 |
| Intracerebral Electrode Implantation | VWR (dark phase) | Sham + NL (Female) | Naïve | 2 | 8 | 8 | 0.22 | -0.72 | 1.14 |
| Intracerebral Electrode Implantation | VWR (dark phase) | Sham + NL (Female) | Naïve | 3 | 8 | 8 | 0.29 | -0.65 | 1.22 |
| Intracerebral Electrode Implantation | VWR (dark phase) | Sham + NLO (Female) | Naïve | 1 | 8 | 8 | 0.18 | -0.76 | 1.10 |
| Intracerebral Electrode Implantation | VWR (dark phase) | Sham + NLO (Female) | Naïve | 2 | 8 | 8 | 0.36 | -0.58 | 1.29 |
| Intracerebral Electrode Implantation | VWR (dark phase) | Sham + NLO (Female) | Naïve | 3 | 8 | 8 | 0.39 | -0.55 | 1.32 |
| Intracerebral Electrode Implantation | VWR (dark phase) | Sham + NO (Female) | Naïve | 1 | 8 | 8 | -0.44 | -1.37 | 0.51 |
| Intracerebral Electrode Implantation | VWR (dark phase) | Sham + NO (Female) | Naïve | 2 | 8 | 8 | -0.04 | -0.97 | 0.88 |
| Intracerebral Electrode Implantation | VWR (dark phase) | Sham + NO (Female) | Naïve | 3 | 8 | 8 | 0.30 | -0.64 | 1.23 |
| Intracerebral Electrode Implantation | VWR (dark phase) | Surgery + N (Female) | Naïve | 1 | 8 | 8 | -0.31 | -1.24 | 0.63 |
| Intracerebral Electrode Implantation | VWR (dark phase) | Surgery + N (Female) | Naïve | 2 | 8 | 8 | -0.10 | -1.03 | 0.83 |
| Intracerebral Electrode Implantation | VWR (dark phase) | Surgery + N (Female) | Naïve | 3 | 8 | 8 | -0.23 | -1.15 | 0.71 |
| Intracerebral Electrode Implantation | VWR (dark phase) | Surgery + NL (Female) | Naïve | 1 | 8 | 8 | -0.88 | -1.85 | 0.12 |
| Intracerebral Electrode Implantation | VWR (dark phase) | Surgery + NL (Female) | Naïve | 2 | 8 | 8 | -0.81 | -1.77 | 0.18 |
| Intracerebral Electrode Implantation | VWR (dark phase) | Surgery + NL (Female) | Naïve | 3 | 8 | 8 | -0.45 | -1.39 | 0.49 |
| Intracerebral Electrode Implantation | VWR (dark phase) | Surgery + NLO (Female) | Naïve | 1 | 8 | 8 | -0.86 | -1.83 | 0.13 |
| Intracerebral Electrode Implantation | VWR (dark phase) | Surgery + NLO (Female) | Naïve | 2 | 8 | 8 | 0.20 | -0.73 | 1.13 |
| Intracerebral Electrode Implantation | VWR (dark phase) | Surgery + NLO (Female) | Naïve | 3 | 8 | 8 | 0.47 | -0.48 | 1.40 |
| Intracerebral Electrode Implantation | VWR (dark phase) | Surgery + NO (Female) | Naïve | 1 | 8 | 8 | -1.49 | -2.54 | -0.39 |
| Intracerebral Electrode Implantation | VWR (dark phase) | Surgery + NO (Female) | Naïve | 2 | 8 | 8 | -0.36 | -1.29 | 0.58 |
| Intracerebral Electrode Implantation | VWR (dark phase) | Surgery + NO (Female) | Naïve | 3 | 8 | 8 | 0.27 | -0.67 | 1.19 |
| Intracerebral Electrode Implantation | VWR (dark phase) | Sham + N (Male) | Naïve | 1 | 8 | 8 | 0.07 | -0.86 | 0.99 |
| Intracerebral Electrode Implantation | VWR (dark phase) | Sham + N (Male) | Naïve | 2 | 8 | 8 | 0.47 | -0.48 | 1.41 |
| Intracerebral Electrode Implantation | VWR (dark phase) | Sham + N (Male) | Naïve | 3 | 7 | 8 | 0.30 | -0.67 | 1.25 |
| Intracerebral Electrode Implantation | VWR (dark phase) | Sham + NL (Male) | Naïve | 1 | 8 | 8 | -0.11 | -1.04 | 0.82 |
| Intracerebral Electrode Implantation | VWR (dark phase) | Sham + NL (Male) | Naïve | 2 | 8 | 8 | 0.35 | -0.59 | 1.28 |
| Intracerebral Electrode Implantation | VWR (dark phase) | Sham + NL (Male) | Naïve | 3 | 7 | 8 | 0.03 | -0.93 | 0.98 |
| Intracerebral Electrode Implantation | VWR (dark phase) | Sham + NLO (Male) | Naïve | 1 | 8 | 8 | -0.79 | -1.75 | 0.19 |
| Intracerebral Electrode Implantation | VWR (dark phase) | Sham + NLO (Male) | Naïve | 2 | 8 | 8 | 0.50 | -0.45 | 1.44 |
| Intracerebral Electrode Implantation | VWR (dark phase) | Sham + NLO (Male) | Naïve | 3 | 7 | 8 | 0.39 | -0.58 | 1.35 |
| Intracerebral Electrode Implantation | VWR (dark phase) | Sham + NO (Male) | Naïve | 1 | 8 | 8 | -0.73 | -1.69 | 0.24 |
| Intracerebral Electrode Implantation | VWR (dark phase) | Sham + NO (Male) | Naïve | 2 | 8 | 8 | 0.24 | -0.69 | 1.17 |
| Intracerebral Electrode Implantation | VWR (dark phase) | Sham + NO (Male) | Naïve | 3 | 7 | 8 | 0.22 | -0.75 | 1.17 |
| Intracerebral Electrode Implantation | VWR (dark phase) | Surgery + N (Male) | Naïve | 1 | 8 | 8 | -0.55 | -1.49 | 0.41 |
| Intracerebral Electrode Implantation | VWR (dark phase) | Surgery + N (Male) | Naïve | 2 | 8 | 8 | 0.25 | -0.68 | 1.18 |
| Intracerebral Electrode Implantation | VWR (dark phase) | Surgery + N (Male) | Naïve | 3 | 7 | 8 | 0.38 | -0.59 | 1.34 |
| Intracerebral Electrode Implantation | VWR (dark phase) | Surgery + NL (Male) | Naïve | 1 | 8 | 8 | -0.57 | -1.51 | 0.39 |
| Intracerebral Electrode Implantation | VWR (dark phase) | Surgery + NL (Male) | Naïve | 2 | 8 | 8 | -0.22 | -1.14 | 0.72 |
| Intracerebral Electrode Implantation | VWR (dark phase) | Surgery + NL (Male) | Naïve | 3 | 7 | 8 | -0.49 | -1.45 | 0.49 |
| Intracerebral Electrode Implantation | VWR (dark phase) | Surgery + NLO (Male) | Naïve | 1 | 8 | 8 | -1.13 | -2.12 | -0.09 |
| Intracerebral Electrode Implantation | VWR (dark phase) | Surgery + NLO (Male) | Naïve | 2 | 8 | 8 | -0.08 | -1.00 | 0.85 |
| Intracerebral Electrode Implantation | VWR (dark phase) | Surgery + NLO (Male) | Naïve | 3 | 8 | 8 | -0.19 | -1.11 | 0.75 |
| Intracerebral Electrode Implantation | VWR (dark phase) | Surgery + NO (Male) | Naïve | 1 | 8 | 8 | -1.09 | -2.08 | -0.06 |
| Intracerebral Electrode Implantation | VWR (dark phase) | Surgery + NO (Male) | Naïve | 2 | 8 | 8 | -0.43 | -1.37 | 0.51 |
| Intracerebral Electrode Implantation | VWR (dark phase) | Surgery + NO (Male) | Naïve | 3 | 7 | 8 | -0.52 | -1.49 | 0.46 |
| Intracerebral Electrode Implantation | VWR (dark phase) | Surgery + N (Female) | Sham (drug-control) | 1 | 8 | 8 | -0.78 | -1.74 | 0.20 |
| Intracerebral Electrode Implantation | VWR (dark phase) | Surgery + N (Female) | Sham (drug-control) | 2 | 8 | 8 | -0.25 | -1.18 | 0.69 |
| Intracerebral Electrode Implantation | VWR (dark phase) | Surgery + N (Female) | Sham (drug-control) | 3 | 8 | 8 | -0.37 | -1.30 | 0.58 |
| Intracerebral Electrode Implantation | VWR (dark phase) | Surgery + N (Male) | Sham (drug-control) | 1 | 8 | 8 | -0.53 | -1.46 | 0.43 |
| Intracerebral Electrode Implantation | VWR (dark phase) | Surgery + N (Male) | Sham (drug-control) | 2 | 8 | 8 | -0.25 | -1.17 | 0.69 |
| Intracerebral Electrode Implantation | VWR (dark phase) | Surgery + N (Male) | Sham (drug-control) | 3 | 7 | 7 | 0.16 | -0.82 | 1.14 |
| Intracerebral Electrode Implantation | VWR (dark phase) | Surgery + NL (Female) | Sham (drug-control) | 1 | 8 | 8 | -1.65 | -2.74 | -0.52 |
| Intracerebral Electrode Implantation | VWR (dark phase) | Surgery + NL (Female) | Sham (drug-control) | 2 | 8 | 8 | -1.32 | -2.35 | -0.26 |
| Intracerebral Electrode Implantation | VWR (dark phase) | Surgery + NL (Female) | Sham (drug-control) | 3 | 8 | 8 | -0.85 | -1.81 | 0.14 |
| Intracerebral Electrode Implantation | VWR (dark phase) | Surgery + NL (Male) | Sham (drug-control) | 1 | 8 | 8 | -0.45 | -1.38 | 0.50 |
| Intracerebral Electrode Implantation | VWR (dark phase) | Surgery + NL (Male) | Sham (drug-control) | 2 | 8 | 8 | -0.56 | -1.50 | 0.40 |
| Intracerebral Electrode Implantation | VWR (dark phase) | Surgery + NL (Male) | Sham (drug-control) | 3 | 7 | 7 | -0.38 | -1.36 | 0.62 |
| Intracerebral Electrode Implantation | VWR (dark phase) | Surgery + NO (Female) | Sham (drug-control) | 1 | 8 | 8 | -1.61 | -2.69 | -0.49 |
| Intracerebral Electrode Implantation | VWR (dark phase) | Surgery + NO (Female) | Sham (drug-control) | 2 | 8 | 8 | -0.34 | -1.27 | 0.60 |
| Intracerebral Electrode Implantation | VWR (dark phase) | Surgery + NO (Female) | Sham (drug-control) | 3 | 8 | 8 | -0.10 | -1.02 | 0.83 |
| Intracerebral Electrode Implantation | VWR (dark phase) | Surgery + NO (Male) | Sham (drug-control) | 1 | 8 | 8 | -0.75 | -1.70 | 0.23 |
| Intracerebral Electrode Implantation | VWR (dark phase) | Surgery + NO (Male) | Sham (drug-control) | 2 | 8 | 8 | -0.66 | -1.60 | 0.31 |
| Intracerebral Electrode Implantation | VWR (dark phase) | Surgery + NO (Male) | Sham (drug-control) | 3 | 7 | 7 | -0.74 | -1.75 | 0.30 |
| Intracerebral Electrode Implantation | VWR (dark phase) | Surgery + NLO (Female) | Sham (drug-control) | 1 | 8 | 8 | -0.85 | -1.82 | 0.14 |
| Intracerebral Electrode Implantation | VWR (dark phase) | Surgery + NLO (Female) | Sham (drug-control) | 2 | 8 | 8 | -0.20 | -1.12 | 0.74 |
| Intracerebral Electrode Implantation | VWR (dark phase) | Surgery + NLO (Female) | Sham (drug-control) | 3 | 8 | 8 | -0.02 | -0.94 | 0.91 |
| Intracerebral Electrode Implantation | VWR (dark phase) | Surgery + NLO (Male) | Sham (drug-control) | 1 | 8 | 8 | -1.06 | -2.05 | -0.04 |
| Intracerebral Electrode Implantation | VWR (dark phase) | Surgery + NLO (Male) | Sham (drug-control) | 2 | 8 | 8 | -0.78 | -1.74 | 0.20 |
| Intracerebral Electrode Implantation | VWR (dark phase) | Surgery + NLO (Male) | Sham (drug-control) | 3 | 8 | 7 | -0.53 | -1.50 | 0.45 |
| Intracerebral Electrode Implantation | HC vm (light phase) | Sham + N (Female) | Naïve | 1 | 8 | 8 | 0.27 | -0.67 | 1.19 |
| Intracerebral Electrode Implantation | HC vm (light phase) | Sham + N (Female) | Naïve | 2 | 8 | 8 | 0.42 | -0.53 | 1.35 |
| Intracerebral Electrode Implantation | HC vm (light phase) | Sham + N (Female) | Naïve | 3 | 8 | 8 | 0.37 | -0.58 | 1.30 |
| Intracerebral Electrode Implantation | HC vm (light phase) | Sham + NL (Female) | Naïve | 1 | 8 | 8 | 0.32 | -0.62 | 1.25 |
| Intracerebral Electrode Implantation | HC vm (light phase) | Sham + NL (Female) | Naïve | 2 | 8 | 8 | 0.76 | -0.22 | 1.72 |
| Intracerebral Electrode Implantation | HC vm (light phase) | Sham + NL (Female) | Naïve | 3 | 8 | 8 | 0.79 | -0.19 | 1.75 |
| Intracerebral Electrode Implantation | HC vm (light phase) | Sham + NLO (Female) | Naïve | 1 | 8 | 8 | -0.38 | -1.31 | 0.56 |
| Intracerebral Electrode Implantation | HC vm (light phase) | Sham + NLO (Female) | Naïve | 2 | 8 | 8 | 0.96 | -0.05 | 1.93 |
| Intracerebral Electrode Implantation | HC vm (light phase) | Sham + NLO (Female) | Naïve | 3 | 8 | 8 | 0.72 | -0.25 | 1.67 |
| Intracerebral Electrode Implantation | HC vm (light phase) | Sham + NO (Female) | Naïve | 1 | 8 | 8 | -0.53 | -1.46 | 0.43 |
| Intracerebral Electrode Implantation | HC vm (light phase) | Sham + NO (Female) | Naïve | 2 | 8 | 8 | 0.52 | -0.44 | 1.45 |
| Intracerebral Electrode Implantation | HC vm (light phase) | Sham + NO (Female) | Naïve | 3 | 8 | 8 | 0.53 | -0.43 | 1.46 |
| Intracerebral Electrode Implantation | HC vm (light phase) | Surgery + N (Female) | Naïve | 1 | 8 | 8 | -0.36 | -1.29 | 0.58 |
| Intracerebral Electrode Implantation | HC vm (light phase) | Surgery + N (Female) | Naïve | 2 | 8 | 8 | 0.41 | -0.54 | 1.34 |
| Intracerebral Electrode Implantation | HC vm (light phase) | Surgery + N (Female) | Naïve | 3 | 8 | 8 | -0.01 | -0.94 | 0.91 |
| Intracerebral Electrode Implantation | HC vm (light phase) | Surgery + NL (Female) | Naïve | 1 | 8 | 8 | -0.50 | -1.44 | 0.45 |
| Intracerebral Electrode Implantation | HC vm (light phase) | Surgery + NL (Female) | Naïve | 2 | 8 | 8 | 0.94 | -0.06 | 1.91 |
| Intracerebral Electrode Implantation | HC vm (light phase) | Surgery + NL (Female) | Naïve | 3 | 8 | 8 | 0.45 | -0.50 | 1.38 |
| Intracerebral Electrode Implantation | HC vm (light phase) | Surgery + NLO (Female) | Naïve | 1 | 8 | 8 | -0.50 | -1.43 | 0.46 |
| Intracerebral Electrode Implantation | HC vm (light phase) | Surgery + NLO (Female) | Naïve | 2 | 8 | 8 | 0.52 | -0.44 | 1.45 |
| Intracerebral Electrode Implantation | HC vm (light phase) | Surgery + NLO (Female) | Naïve | 3 | 8 | 8 | 0.92 | -0.08 | 1.89 |
| Intracerebral Electrode Implantation | HC vm (light phase) | Surgery + NO (Female) | Naïve | 1 | 8 | 8 | -0.81 | -1.77 | 0.18 |
| Intracerebral Electrode Implantation | HC vm (light phase) | Surgery + NO (Female) | Naïve | 2 | 8 | 8 | -0.31 | -1.23 | 0.63 |
| Intracerebral Electrode Implantation | HC vm (light phase) | Surgery + NO (Female) | Naïve | 3 | 8 | 8 | -0.09 | -1.01 | 0.84 |
| Intracerebral Electrode Implantation | HC vm (light phase) | Sham + N (Male) | Naïve | 1 | 8 | 8 | -0.16 | -1.08 | 0.77 |
| Intracerebral Electrode Implantation | HC vm (light phase) | Sham + N (Male) | Naïve | 2 | 8 | 8 | 0.56 | -0.40 | 1.50 |
| Intracerebral Electrode Implantation | HC vm (light phase) | Sham + N (Male) | Naïve | 3 | 8 | 8 | 0.52 | -0.43 | 1.46 |
| Intracerebral Electrode Implantation | HC vm (light phase) | Sham + NL (Male) | Naïve | 1 | 8 | 8 | -0.57 | -1.51 | 0.39 |
| Intracerebral Electrode Implantation | HC vm (light phase) | Sham + NL (Male) | Naïve | 2 | 8 | 8 | 0.86 | -0.13 | 1.82 |
| Intracerebral Electrode Implantation | HC vm (light phase) | Sham + NL (Male) | Naïve | 3 | 8 | 8 | 0.66 | -0.31 | 1.61 |
| Intracerebral Electrode Implantation | HC vm (light phase) | Sham + NLO (Male) | Naïve | 1 | 8 | 8 | -0.96 | -1.94 | 0.04 |
| Intracerebral Electrode Implantation | HC vm (light phase) | Sham + NLO (Male) | Naïve | 2 | 8 | 8 | 1.03 | 0.01 | 2.01 |
| Intracerebral Electrode Implantation | HC vm (light phase) | Sham + NLO (Male) | Naïve | 3 | 8 | 8 | 0.48 | -0.47 | 1.41 |
| Intracerebral Electrode Implantation | HC vm (light phase) | Sham + NO (Male) | Naïve | 1 | 8 | 8 | -0.73 | -1.69 | 0.24 |
| Intracerebral Electrode Implantation | HC vm (light phase) | Sham + NO (Male) | Naïve | 2 | 8 | 8 | 0.42 | -0.52 | 1.35 |
| Intracerebral Electrode Implantation | HC vm (light phase) | Sham + NO (Male) | Naïve | 3 | 8 | 8 | 0.43 | -0.52 | 1.36 |
| Intracerebral Electrode Implantation | HC vm (light phase) | Surgery + N (Male) | Naïve | 1 | 8 | 8 | -0.99 | -1.97 | 0.02 |
| Intracerebral Electrode Implantation | HC vm (light phase) | Surgery + N (Male) | Naïve | 2 | 8 | 8 | -0.17 | -1.09 | 0.76 |
| Intracerebral Electrode Implantation | HC vm (light phase) | Surgery + N (Male) | Naïve | 3 | 8 | 8 | -0.64 | -1.58 | 0.33 |
| Intracerebral Electrode Implantation | HC vm (light phase) | Surgery + NL (Male) | Naïve | 1 | 8 | 8 | -1.26 | -2.28 | -0.21 |
| Intracerebral Electrode Implantation | HC vm (light phase) | Surgery + NL (Male) | Naïve | 2 | 8 | 8 | 0.57 | -0.39 | 1.51 |
| Intracerebral Electrode Implantation | HC vm (light phase) | Surgery + NL (Male) | Naïve | 3 | 8 | 8 | 0.21 | -0.73 | 1.13 |
| Intracerebral Electrode Implantation | HC vm (light phase) | Surgery + NLO (Male) | Naïve | 1 | 8 | 8 | -1.03 | -2.01 | -0.01 |
| Intracerebral Electrode Implantation | HC vm (light phase) | Surgery + NLO (Male) | Naïve | 2 | 8 | 8 | 0.27 | -0.66 | 1.20 |
| Intracerebral Electrode Implantation | HC vm (light phase) | Surgery + NLO (Male) | Naïve | 3 | 8 | 8 | -0.01 | -0.93 | 0.92 |
| Intracerebral Electrode Implantation | HC vm (light phase) | Surgery + NO (Male) | Naïve | 1 | 8 | 8 | -0.93 | -1.91 | 0.07 |
| Intracerebral Electrode Implantation | HC vm (light phase) | Surgery + NO (Male) | Naïve | 2 | 8 | 8 | 0.05 | -0.87 | 0.98 |
| Intracerebral Electrode Implantation | HC vm (light phase) | Surgery + NO (Male) | Naïve | 3 | 8 | 8 | 0.27 | -0.67 | 1.20 |
| Intracerebral Electrode Implantation | HC vm (light phase) | Surgery + N (Female) | Sham (drug-control) | 1 | 8 | 8 | -0.78 | -1.74 | 0.20 |
| Intracerebral Electrode Implantation | HC vm (light phase) | Surgery + N (Female) | Sham (drug-control) | 2 | 8 | 8 | 0.12 | -0.81 | 1.05 |
| Intracerebral Electrode Implantation | HC vm (light phase) | Surgery + N (Female) | Sham (drug-control) | 3 | 8 | 8 | -0.33 | -1.26 | 0.61 |
| Intracerebral Electrode Implantation | HC vm (light phase) | Surgery + N (Male) | Sham (drug-control) | 1 | 8 | 8 | -1.11 | -2.11 | -0.09 |
| Intracerebral Electrode Implantation | HC vm (light phase) | Surgery + N (Male) | Sham (drug-control) | 2 | 8 | 8 | -0.64 | -1.59 | 0.32 |
| Intracerebral Electrode Implantation | HC vm (light phase) | Surgery + N (Male) | Sham (drug-control) | 3 | 8 | 8 | -0.78 | -1.74 | 0.20 |
| Intracerebral Electrode Implantation | HC vm (light phase) | Surgery + NL (Female) | Sham (drug-control) | 1 | 8 | 8 | -1.05 | -2.03 | -0.03 |
| Intracerebral Electrode Implantation | HC vm (light phase) | Surgery + NL (Female) | Sham (drug-control) | 2 | 8 | 8 | -0.22 | -1.15 | 0.71 |
| Intracerebral Electrode Implantation | HC vm (light phase) | Surgery + NL (Female) | Sham (drug-control) | 3 | 8 | 8 | -0.72 | -1.67 | 0.25 |
| Intracerebral Electrode Implantation | HC vm (light phase) | Surgery + NL (Male) | Sham (drug-control) | 1 | 8 | 8 | -0.78 | -1.74 | 0.20 |
| Intracerebral Electrode Implantation | HC vm (light phase) | Surgery + NL (Male) | Sham (drug-control) | 2 | 8 | 8 | -0.61 | -1.55 | 0.36 |
| Intracerebral Electrode Implantation | HC vm (light phase) | Surgery + NL (Male) | Sham (drug-control) | 3 | 8 | 8 | -0.55 | -1.49 | 0.41 |
| Intracerebral Electrode Implantation | HC vm (light phase) | Surgery + NO (Female) | Sham (drug-control) | 1 | 8 | 8 | -1.14 | -2.13 | -0.10 |
| Intracerebral Electrode Implantation | HC vm (light phase) | Surgery + NO (Female) | Sham (drug-control) | 2 | 8 | 8 | -0.54 | -1.48 | 0.41 |
| Intracerebral Electrode Implantation | HC vm (light phase) | Surgery + NO (Female) | Sham (drug-control) | 3 | 8 | 8 | -0.53 | -1.47 | 0.42 |
| Intracerebral Electrode Implantation | HC vm (light phase) | Surgery + NO (Male) | Sham (drug-control) | 1 | 8 | 8 | -0.37 | -1.30 | 0.58 |
| Intracerebral Electrode Implantation | HC vm (light phase) | Surgery + NO (Male) | Sham (drug-control) | 2 | 8 | 8 | -0.36 | -1.29 | 0.58 |
| Intracerebral Electrode Implantation | HC vm (light phase) | Surgery + NO (Male) | Sham (drug-control) | 3 | 8 | 8 | -0.13 | -1.05 | 0.80 |
| Intracerebral Electrode Implantation | HC vm (light phase) | Surgery + NLO (Female) | Sham (drug-control) | 1 | 8 | 8 | -0.33 | -1.25 | 0.61 |
| Intracerebral Electrode Implantation | HC vm (light phase) | Surgery + NLO (Female) | Sham (drug-control) | 2 | 8 | 8 | -0.22 | -1.14 | 0.71 |
| Intracerebral Electrode Implantation | HC vm (light phase) | Surgery + NLO (Female) | Sham (drug-control) | 3 | 8 | 8 | 0.18 | -0.76 | 1.10 |
| Intracerebral Electrode Implantation | HC vm (light phase) | Surgery + NLO (Male) | Sham (drug-control) | 1 | 8 | 8 | -0.27 | -1.19 | 0.67 |
| Intracerebral Electrode Implantation | HC vm (light phase) | Surgery + NLO (Male) | Sham (drug-control) | 2 | 8 | 8 | -0.77 | -1.72 | 0.21 |
| Intracerebral Electrode Implantation | HC vm (light phase) | Surgery + NLO (Male) | Sham (drug-control) | 3 | 8 | 8 | -0.46 | -1.39 | 0.49 |
| Intracerebral Electrode Implantation | HC vm (dark phase) | Sham + N (Female) | Naïve | 0 | 8 | 8 | -0.42 | -1.35 | 0.53 |
| Intracerebral Electrode Implantation | HC vm (dark phase) | Sham + N (Female) | Naïve | 1 | 8 | 8 | 0.16 | -0.77 | 1.09 |
| Intracerebral Electrode Implantation | HC vm (dark phase) | Sham + N (Female) | Naïve | 2 | 8 | 8 | 0.20 | -0.73 | 1.13 |
| Intracerebral Electrode Implantation | HC vm (dark phase) | Sham + N (Female) | Naïve | 3 | 8 | 8 | 0.28 | -0.66 | 1.20 |
| Intracerebral Electrode Implantation | HC vm (dark phase) | Sham + NL (Female) | Naïve | 0 | 8 | 8 | -0.25 | -1.18 | 0.68 |
| Intracerebral Electrode Implantation | HC vm (dark phase) | Sham + NL (Female) | Naïve | 1 | 8 | 8 | -0.41 | -1.34 | 0.54 |
| Intracerebral Electrode Implantation | HC vm (dark phase) | Sham + NL (Female) | Naïve | 2 | 8 | 8 | -0.22 | -1.14 | 0.72 |
| Intracerebral Electrode Implantation | HC vm (dark phase) | Sham + NL (Female) | Naïve | 3 | 8 | 8 | 0.13 | -0.80 | 1.05 |
| Intracerebral Electrode Implantation | HC vm (dark phase) | Sham + NLO (Female) | Naïve | 0 | 8 | 8 | -0.51 | -1.45 | 0.44 |
| Intracerebral Electrode Implantation | HC vm (dark phase) | Sham + NLO (Female) | Naïve | 1 | 8 | 8 | 0.08 | -0.85 | 1.00 |
| Intracerebral Electrode Implantation | HC vm (dark phase) | Sham + NLO (Female) | Naïve | 2 | 8 | 8 | 0.36 | -0.58 | 1.29 |
| Intracerebral Electrode Implantation | HC vm (dark phase) | Sham + NLO (Female) | Naïve | 3 | 8 | 8 | 0.49 | -0.46 | 1.43 |
| Intracerebral Electrode Implantation | HC vm (dark phase) | Sham + NO (Female) | Naïve | 0 | 8 | 8 | -0.56 | -1.50 | 0.40 |
| Intracerebral Electrode Implantation | HC vm (dark phase) | Sham + NO (Female) | Naïve | 1 | 8 | 8 | -0.27 | -1.19 | 0.67 |
| Intracerebral Electrode Implantation | HC vm (dark phase) | Sham + NO (Female) | Naïve | 2 | 8 | 8 | 0.22 | -0.72 | 1.14 |
| Intracerebral Electrode Implantation | HC vm (dark phase) | Sham + NO (Female) | Naïve | 3 | 8 | 8 | 0.24 | -0.70 | 1.16 |
| Intracerebral Electrode Implantation | HC vm (dark phase) | Surgery + N (Female) | Naïve | 0 | 8 | 8 | -1.78 | -2.89 | -0.62 |
| Intracerebral Electrode Implantation | HC vm (dark phase) | Surgery + N (Female) | Naïve | 1 | 8 | 8 | -0.87 | -1.83 | 0.13 |
| Intracerebral Electrode Implantation | HC vm (dark phase) | Surgery + N (Female) | Naïve | 2 | 8 | 8 | 0.05 | -0.87 | 0.98 |
| Intracerebral Electrode Implantation | HC vm (dark phase) | Surgery + N (Female) | Naïve | 3 | 8 | 8 | -0.42 | -1.35 | 0.53 |
| Intracerebral Electrode Implantation | HC vm (dark phase) | Surgery + NL (Female) | Naïve | 0 | 8 | 8 | -1.63 | -2.71 | -0.51 |
| Intracerebral Electrode Implantation | HC vm (dark phase) | Surgery + NL (Female) | Naïve | 1 | 8 | 8 | -1.62 | -2.70 | -0.50 |
| Intracerebral Electrode Implantation | HC vm (dark phase) | Surgery + NL (Female) | Naïve | 2 | 8 | 8 | -1.18 | -2.19 | -0.14 |
| Intracerebral Electrode Implantation | HC vm (dark phase) | Surgery + NL (Female) | Naïve | 3 | 8 | 8 | -0.73 | -1.69 | 0.24 |
| Intracerebral Electrode Implantation | HC vm (dark phase) | Surgery + NLO (Female) | Naïve | 0 | 8 | 8 | -2.24 | -3.45 | -0.98 |
| Intracerebral Electrode Implantation | HC vm (dark phase) | Surgery + NLO (Female) | Naïve | 1 | 8 | 8 | -1.66 | -2.75 | -0.53 |
| Intracerebral Electrode Implantation | HC vm (dark phase) | Surgery + NLO (Female) | Naïve | 2 | 8 | 8 | 0.30 | -0.64 | 1.23 |
| Intracerebral Electrode Implantation | HC vm (dark phase) | Surgery + NLO (Female) | Naïve | 3 | 8 | 8 | 0.44 | -0.50 | 1.38 |
| Intracerebral Electrode Implantation | HC vm (dark phase) | Surgery + NO (Female) | Naïve | 0 | 8 | 8 | -1.93 | -3.07 | -0.74 |
| Intracerebral Electrode Implantation | HC vm (dark phase) | Surgery + NO (Female) | Naïve | 1 | 8 | 8 | -2.14 | -3.33 | -0.90 |
| Intracerebral Electrode Implantation | HC vm (dark phase) | Surgery + NO (Female) | Naïve | 2 | 8 | 8 | -0.59 | -1.53 | 0.37 |
| Intracerebral Electrode Implantation | HC vm (dark phase) | Surgery + NO (Female) | Naïve | 3 | 8 | 8 | 0.04 | -0.89 | 0.96 |
| Intracerebral Electrode Implantation | HC vm (dark phase) | Sham + N (Male) | Naïve | 0 | 8 | 8 | -0.29 | -1.22 | 0.64 |
| Intracerebral Electrode Implantation | HC vm (dark phase) | Sham + N (Male) | Naïve | 1 | 8 | 8 | 0.43 | -0.52 | 1.36 |
| Intracerebral Electrode Implantation | HC vm (dark phase) | Sham + N (Male) | Naïve | 2 | 8 | 8 | 0.90 | -0.09 | 1.87 |
| Intracerebral Electrode Implantation | HC vm (dark phase) | Sham + N (Male) | Naïve | 3 | 7 | 8 | 0.73 | -0.28 | 1.71 |
| Intracerebral Electrode Implantation | HC vm (dark phase) | Sham + NL (Male) | Naïve | 0 | 8 | 8 | -0.56 | -1.50 | 0.40 |
| Intracerebral Electrode Implantation | HC vm (dark phase) | Sham + NL (Male) | Naïve | 1 | 8 | 8 | -0.07 | -1.00 | 0.86 |
| Intracerebral Electrode Implantation | HC vm (dark phase) | Sham + NL (Male) | Naïve | 2 | 8 | 8 | 0.35 | -0.59 | 1.28 |
| Intracerebral Electrode Implantation | HC vm (dark phase) | Sham + NL (Male) | Naïve | 3 | 7 | 8 | -0.16 | -1.12 | 0.80 |
| Intracerebral Electrode Implantation | HC vm (dark phase) | Sham + NLO (Male) | Naïve | 0 | 8 | 8 | -0.20 | -1.13 | 0.73 |
| Intracerebral Electrode Implantation | HC vm (dark phase) | Sham + NLO (Male) | Naïve | 1 | 8 | 8 | -0.59 | -1.53 | 0.38 |
| Intracerebral Electrode Implantation | HC vm (dark phase) | Sham + NLO (Male) | Naïve | 2 | 8 | 8 | 1.21 | 0.16 | 2.22 |
| Intracerebral Electrode Implantation | HC vm (dark phase) | Sham + NLO (Male) | Naïve | 3 | 7 | 8 | 1.11 | 0.05 | 2.14 |
| Intracerebral Electrode Implantation | HC vm (dark phase) | Sham + NO (Male) | Naïve | 0 | 8 | 8 | -0.90 | -1.87 | 0.10 |
| Intracerebral Electrode Implantation | HC vm (dark phase) | Sham + NO (Male) | Naïve | 1 | 8 | 8 | -0.83 | -1.79 | 0.16 |
| Intracerebral Electrode Implantation | HC vm (dark phase) | Sham + NO (Male) | Naïve | 2 | 8 | 8 | 0.43 | -0.52 | 1.36 |
| Intracerebral Electrode Implantation | HC vm (dark phase) | Sham + NO (Male) | Naïve | 3 | 7 | 8 | 0.38 | -0.60 | 1.33 |
| Intracerebral Electrode Implantation | HC vm (dark phase) | Surgery + N (Male) | Naïve | 0 | 8 | 8 | -1.20 | -2.21 | -0.16 |
| Intracerebral Electrode Implantation | HC vm (dark phase) | Surgery + N (Male) | Naïve | 1 | 8 | 8 | -0.23 | -1.16 | 0.70 |
| Intracerebral Electrode Implantation | HC vm (dark phase) | Surgery + N (Male) | Naïve | 2 | 8 | 8 | 0.13 | -0.80 | 1.06 |
| Intracerebral Electrode Implantation | HC vm (dark phase) | Surgery + N (Male) | Naïve | 3 | 5 | 8 | 0.10 | -0.94 | 1.14 |
| Intracerebral Electrode Implantation | HC vm (dark phase) | Surgery + NL (Male) | Naïve | 0 | 8 | 8 | -1.52 | -2.59 | -0.42 |
| Intracerebral Electrode Implantation | HC vm (dark phase) | Surgery + NL (Male) | Naïve | 1 | 8 | 8 | -0.81 | -1.77 | 0.18 |
| Intracerebral Electrode Implantation | HC vm (dark phase) | Surgery + NL (Male) | Naïve | 2 | 8 | 8 | -0.42 | -1.36 | 0.52 |
| Intracerebral Electrode Implantation | HC vm (dark phase) | Surgery + NL (Male) | Naïve | 3 | 7 | 8 | -0.56 | -1.53 | 0.43 |
| Intracerebral Electrode Implantation | HC vm (dark phase) | Surgery + NLO (Male) | Naïve | 0 | 8 | 8 | -1.69 | -2.78 | -0.55 |
| Intracerebral Electrode Implantation | HC vm (dark phase) | Surgery + NLO (Male) | Naïve | 1 | 8 | 8 | -1.19 | -2.20 | -0.15 |
| Intracerebral Electrode Implantation | HC vm (dark phase) | Surgery + NLO (Male) | Naïve | 2 | 8 | 8 | 0.25 | -0.68 | 1.18 |
| Intracerebral Electrode Implantation | HC vm (dark phase) | Surgery + NLO (Male) | Naïve | 3 | 7 | 8 | 0.40 | -0.57 | 1.36 |
| Intracerebral Electrode Implantation | HC vm (dark phase) | Surgery + NO (Male) | Naïve | 0 | 8 | 8 | -1.49 | -2.55 | -0.39 |
| Intracerebral Electrode Implantation | HC vm (dark phase) | Surgery + NO (Male) | Naïve | 1 | 8 | 8 | -1.23 | -2.24 | -0.18 |
| Intracerebral Electrode Implantation | HC vm (dark phase) | Surgery + NO (Male) | Naïve | 2 | 8 | 8 | -0.36 | -1.29 | 0.59 |
| Intracerebral Electrode Implantation | HC vm (dark phase) | Surgery + NO (Male) | Naïve | 3 | 7 | 8 | 0.09 | -0.86 | 1.05 |
| Intracerebral Electrode Implantation | HC vm (dark phase) | Surgery + N (Female) | Sham (drug-control) | 0 | 8 | 8 | -1.13 | -2.13 | -0.10 |
| Intracerebral Electrode Implantation | HC vm (dark phase) | Surgery + N (Female) | Sham (drug-control) | 1 | 8 | 8 | -0.78 | -1.74 | 0.20 |
| Intracerebral Electrode Implantation | HC vm (dark phase) | Surgery + N (Female) | Sham (drug-control) | 2 | 8 | 8 | -0.19 | -1.11 | 0.75 |
| Intracerebral Electrode Implantation | HC vm (dark phase) | Surgery + N (Female) | Sham (drug-control) | 3 | 8 | 8 | -0.56 | -1.50 | 0.40 |
| Intracerebral Electrode Implantation | HC vm (dark phase) | Surgery + N (Male) | Sham (drug-control) | 0 | 8 | 8 | -0.81 | -1.77 | 0.18 |
| Intracerebral Electrode Implantation | HC vm (dark phase) | Surgery + N (Male) | Sham (drug-control) | 1 | 8 | 8 | -0.60 | -1.55 | 0.36 |
| Intracerebral Electrode Implantation | HC vm (dark phase) | Surgery + N (Male) | Sham (drug-control) | 2 | 8 | 8 | -0.85 | -1.81 | 0.14 |
| Intracerebral Electrode Implantation | HC vm (dark phase) | Surgery + N (Male) | Sham (drug-control) | 3 | 5 | 7 | -0.67 | -1.75 | 0.45 |
| Intracerebral Electrode Implantation | HC vm (dark phase) | Surgery + NL (Female) | Sham (drug-control) | 0 | 8 | 8 | -1.37 | -2.40 | -0.30 |
| Intracerebral Electrode Implantation | HC vm (dark phase) | Surgery + NL (Female) | Sham (drug-control) | 1 | 8 | 8 | -1.49 | -2.55 | -0.40 |
| Intracerebral Electrode Implantation | HC vm (dark phase) | Surgery + NL (Female) | Sham (drug-control) | 2 | 8 | 8 | -1.29 | -2.32 | -0.23 |
| Intracerebral Electrode Implantation | HC vm (dark phase) | Surgery + NL (Female) | Sham (drug-control) | 3 | 8 | 8 | -0.83 | -1.79 | 0.16 |
| Intracerebral Electrode Implantation | HC vm (dark phase) | Surgery + NL (Male) | Sham (drug-control) | 0 | 8 | 8 | -1.72 | -2.82 | -0.58 |
| Intracerebral Electrode Implantation | HC vm (dark phase) | Surgery + NL (Male) | Sham (drug-control) | 1 | 8 | 8 | -0.52 | -1.46 | 0.44 |
| Intracerebral Electrode Implantation | HC vm (dark phase) | Surgery + NL (Male) | Sham (drug-control) | 2 | 8 | 8 | -0.93 | -1.90 | 0.07 |
| Intracerebral Electrode Implantation | HC vm (dark phase) | Surgery + NL (Male) | Sham (drug-control) | 3 | 7 | 7 | -0.44 | -1.43 | 0.57 |
| Intracerebral Electrode Implantation | HC vm (dark phase) | Surgery + NO (Female) | Sham (drug-control) | 0 | 8 | 8 | -1.47 | -2.52 | -0.38 |
| Intracerebral Electrode Implantation | HC vm (dark phase) | Surgery + NO (Female) | Sham (drug-control) | 1 | 8 | 8 | -2.05 | -3.22 | -0.83 |
| Intracerebral Electrode Implantation | HC vm (dark phase) | Surgery + NO (Female) | Sham (drug-control) | 2 | 8 | 8 | -0.79 | -1.75 | 0.19 |
| Intracerebral Electrode Implantation | HC vm (dark phase) | Surgery + NO (Female) | Sham (drug-control) | 3 | 8 | 8 | -0.20 | -1.13 | 0.73 |
| Intracerebral Electrode Implantation | HC vm (dark phase) | Surgery + NO (Male) | Sham (drug-control) | 0 | 8 | 8 | -0.96 | -1.93 | 0.05 |
| Intracerebral Electrode Implantation | HC vm (dark phase) | Surgery + NO (Male) | Sham (drug-control) | 1 | 8 | 8 | -0.66 | -1.61 | 0.31 |
| Intracerebral Electrode Implantation | HC vm (dark phase) | Surgery + NO (Male) | Sham (drug-control) | 2 | 8 | 8 | -0.72 | -1.67 | 0.26 |
| Intracerebral Electrode Implantation | HC vm (dark phase) | Surgery + NO (Male) | Sham (drug-control) | 3 | 7 | 7 | -0.29 | -1.27 | 0.70 |
| Intracerebral Electrode Implantation | HC vm (dark phase) | Surgery + NLO (Female) | Sham (drug-control) | 0 | 8 | 8 | -1.21 | -2.22 | -0.17 |
| Intracerebral Electrode Implantation | HC vm (dark phase) | Surgery + NLO (Female) | Sham (drug-control) | 1 | 8 | 8 | -1.02 | -2.01 | -0.01 |
| Intracerebral Electrode Implantation | HC vm (dark phase) | Surgery + NLO (Female) | Sham (drug-control) | 2 | 8 | 8 | -0.15 | -1.07 | 0.79 |
| Intracerebral Electrode Implantation | HC vm (dark phase) | Surgery + NLO (Female) | Sham (drug-control) | 3 | 8 | 8 | -0.13 | -1.06 | 0.80 |
| Intracerebral Electrode Implantation | HC vm (dark phase) | Surgery + NLO (Male) | Sham (drug-control) | 0 | 8 | 8 | -3.28 | -4.78 | -1.74 |
| Intracerebral Electrode Implantation | HC vm (dark phase) | Surgery + NLO (Male) | Sham (drug-control) | 1 | 8 | 8 | -0.83 | -1.79 | 0.16 |
| Intracerebral Electrode Implantation | HC vm (dark phase) | Surgery + NLO (Male) | Sham (drug-control) | 2 | 8 | 8 | -1.19 | -2.20 | -0.15 |
| Intracerebral Electrode Implantation | HC vm (dark phase) | Surgery + NLO (Male) | Sham (drug-control) | 3 | 7 | 7 | -0.70 | -1.70 | 0.33 |
| Subarachnoid Hemorrhage (SAH) Model | Clinical Score | SAH | Sham | 0 | 20 | 19 | 0.44 | -0.79 | 1.67 |
| Subarachnoid Hemorrhage (SAH) Model | Clinical Score | SAH | Sham | 1 | 18 | 19 | 0.54 | -0.65 | 1.73 |
| Subarachnoid Hemorrhage (SAH) Model | Clinical Score | SAH | Sham | 2 | 11 | 17 | 0.39 | -1.00 | 1.78 |
| Subarachnoid Hemorrhage (SAH) Model | Clinical Score | SAH | Sham (drug-control) | 1 | 18 | 5 | 1.91 | 0.65 | 3.18 |
| Subarachnoid Hemorrhage (SAH) Model | Clinical Score | SAH | Sham (drug-control) | 2 | 11 | 5 | 1.23 | -0.37 | 2.83 |
| Subarachnoid Hemorrhage (SAH) Model | Clinical Score | SAH | Baseline | 0 | 20 | 20 | 2.01 | 1.15 | 2.87 |
| Subarachnoid Hemorrhage (SAH) Model | Clinical Score | SAH | Baseline | 1 | 18 | 20 | 1.91 | 1.00 | 2.82 |
| Subarachnoid Hemorrhage (SAH) Model | Clinical Score | SAH | Baseline | 2 | 11 | 20 | 1.23 | 0.02 | 2.44 |
| Subarachnoid Hemorrhage (SAH) Model | Clinical Score | Sham | Baseline | 0 | 19 | 19 | 1.57 | 0.49 | 2.65 |
| Subarachnoid Hemorrhage (SAH) Model | Clinical Score | Sham | Baseline | 1 | 19 | 19 | 1.37 | 0.38 | 2.35 |
| Subarachnoid Hemorrhage (SAH) Model | Clinical Score | Sham | Baseline | 2 | 17 | 19 | 0.84 | -0.08 | 1.76 |
| Subarachnoid Hemorrhage (SAH) Model | Neuroscore | SAH | Sham | 0 | 20 | 19 | 0.44 | -0.79 | 1.67 |
| Subarachnoid Hemorrhage (SAH) Model | Neuroscore | SAH | Sham | 1 | 18 | 19 | 0.87 | -0.22 | 1.96 |
| Subarachnoid Hemorrhage (SAH) Model | Neuroscore | SAH | Sham | 2 | 11 | 17 | 0.68 | -0.71 | 2.07 |
| Subarachnoid Hemorrhage (SAH) Model | Neuroscore | SAH | Baseline | 0 | 20 | 20 | 2.01 | 0.87 | 3.16 |
| Subarachnoid Hemorrhage (SAH) Model | Neuroscore | SAH | Baseline | 1 | 18 | 20 | 1.71 | 0.57 | 2.86 |
| Subarachnoid Hemorrhage (SAH) Model | Neuroscore | SAH | Baseline | 2 | 11 | 20 | 1.91 | 0.48 | 3.34 |
| Subarachnoid Hemorrhage (SAH) Model | Neuroscore | Sham | Baseline | 0 | 19 | 19 | 1.57 | 0.31 | 2.84 |
| Subarachnoid Hemorrhage (SAH) Model | Neuroscore | Sham | Baseline | 1 | 19 | 19 | 0.84 | -0.29 | 1.97 |
| Subarachnoid Hemorrhage (SAH) Model | Neuroscore | Sham | Baseline | 2 | 17 | 19 | 1.23 | 0.10 | 2.36 |
| Subarachnoid Hemorrhage (SAH) Model | Open field | SAH | Sham (drug-control) | -1 | 13 | 5 | 0.83 | -0.21 | 1.84 |
| Subarachnoid Hemorrhage (SAH) Model | Open field | SAH | Sham (drug-control) | 1 | 11 | 5 | -0.84 | -1.88 | 0.22 |
| Subarachnoid Hemorrhage (SAH) Model | Open field | SAH | Sham (drug-control) | 3 | 9 | 5 | -2.11 | -3.40 | -0.76 |
| Subarachnoid Hemorrhage (SAH) Model | Open field | SAH | Sham (drug-control) | 4 | 6 | 5 | 0.39 | -0.72 | 1.48 |
| Subarachnoid Hemorrhage (SAH) Model | Open field | SAH | Sham (drug-control) | 7 | 6 | 5 | 0.55 | -0.58 | 1.65 |
| Subarachnoid Hemorrhage (SAH) Model | Open field | SAH | Sham | -1 | 13 | 12 | -0.28 | -1.04 | 0.48 |
| Subarachnoid Hemorrhage (SAH) Model | Open field | SAH | Sham | 1 | 11 | 12 | 0.17 | -0.63 | 0.95 |
| Subarachnoid Hemorrhage (SAH) Model | Open field | SAH | Sham | 3 | 9 | 12 | -0.47 | -1.30 | 0.38 |
| Subarachnoid Hemorrhage (SAH) Model | Open field | SAH | Sham | 4 | 6 | 8 | 0.36 | -0.65 | 1.35 |
| Subarachnoid Hemorrhage (SAH) Model | Open field | SAH | Sham | 7 | 6 | 8 | 0.16 | -0.84 | 1.15 |
| Subarachnoid Hemorrhage (SAH) Model | Open field | SAH | Baseline | 1 | 11 | 13 | -0.93 | -1.74 | -0.10 |
| Subarachnoid Hemorrhage (SAH) Model | Open field | SAH | Baseline | 3 | 9 | 13 | -0.59 | -1.42 | 0.25 |
| Subarachnoid Hemorrhage (SAH) Model | Open field | SAH | Baseline | 4 | 6 | 13 | -0.19 | -1.11 | 0.74 |
| Subarachnoid Hemorrhage (SAH) Model | Open field | SAH | Baseline | 7 | 6 | 13 | 0.09 | -0.84 | 1.01 |
| Subarachnoid Hemorrhage (SAH) Model | Open field | Sham (drug-control) | Baseline | 1 | 5 | 5 | 1.16 | -0.12 | 2.38 |
| Subarachnoid Hemorrhage (SAH) Model | Open field | Sham (drug-control) | Baseline | 3 | 5 | 5 | 2.58 | 0.87 | 4.22 |
| Subarachnoid Hemorrhage (SAH) Model | Open field | Sham (drug-control) | Baseline | 4 | 5 | 5 | 0.47 | -0.68 | 1.60 |
| Subarachnoid Hemorrhage (SAH) Model | Open field | Sham (drug-control) | Baseline | 7 | 5 | 5 | 0.62 | -0.55 | 1.76 |
| Subarachnoid Hemorrhage (SAH) Model | Open field | Sham | Baseline | 1 | 12 | 12 | -1.62 | -2.51 | -0.70 |
| Subarachnoid Hemorrhage (SAH) Model | Open field | Sham | Baseline | 3 | 12 | 12 | -0.14 | -0.91 | 0.63 |
| Subarachnoid Hemorrhage (SAH) Model | Open field | Sham | Baseline | 4 | 8 | 12 | -0.88 | -1.77 | 0.03 |
| Subarachnoid Hemorrhage (SAH) Model | Open field | Sham | Baseline | 7 | 8 | 12 | -0.38 | -1.24 | 0.49 |
| Partial Hepatectomy (PH) | Clinical Score | PH 50 (Male) | Sham | 1 | 63 | 10 | 0.51 | -0.15 | 1.18 |
| Partial Hepatectomy (PH) | Clinical Score | PH 70 (Male) | Sham | 1 | 58 | 10 | 0.26 | -0.41 | 0.93 |
| Partial Hepatectomy (PH) | Open field | PH 50 (Male) | Sham | -1 | 63 | 10 | 0.32 | -0.34 | 0.98 |
| Partial Hepatectomy (PH) | Open field | PH 50 (Male) | Sham | 1 | 63 | 10 | 0.22 | -0.44 | 0.88 |
| Partial Hepatectomy (PH) | Open field | PH 50 (Male) | Sham | 3 | 42 | 10 | -0.12 | -0.80 | 0.56 |
| Partial Hepatectomy (PH) | Open field | PH 50 (Male) | Sham | 4 | 21 | 10 | -0.03 | -0.76 | 0.70 |
| Partial Hepatectomy (PH) | Open field | PH 50 (Male) | Sham | 7 | 21 | 10 | 0.53 | -0.22 | 1.27 |
| Partial Hepatectomy (PH) | Open field | PH 70 (Male) | Sham | -1 | 63 | 10 | 0.99 | 0.31 | 1.67 |
| Partial Hepatectomy (PH) | Open field | PH 70 (Male) | Sham | 1 | 58 | 10 | 0.13 | -0.53 | 0.80 |
| Partial Hepatectomy (PH) | Open field | PH 70 (Male) | Sham | 3 | 37 | 10 | -0.15 | -0.83 | 0.54 |
| Partial Hepatectomy (PH) | Open field | PH 70 (Male) | Sham | 4 | 18 | 10 | -0.02 | -0.77 | 0.73 |
| Partial Hepatectomy (PH) | Open field | PH 70 (Male) | Sham | 7 | 18 | 10 | -0.07 | -0.82 | 0.68 |
| Partial Hepatectomy (PH) | Open field | Sham (Male) | Baseline | 1 | 10 | 10 | -0.41 | -1.26 | 0.44 |
| Partial Hepatectomy (PH) | Open field | Sham (Male) | Baseline | 3 | 10 | 10 | 0.20 | -0.65 | 1.04 |
| Partial Hepatectomy (PH) | Open field | Sham (Male) | Baseline | 4 | 10 | 10 | 0.53 | -0.34 | 1.38 |
| Partial Hepatectomy (PH) | Open field | Sham (Male) | Baseline | 7 | 10 | 10 | 0.82 | -0.07 | 1.69 |
| Partial Hepatectomy (PH) | Open field | PH 50 (Male) | Baseline | 1 | 63 | 63 | -0.60 | -0.95 | -0.24 |
| Partial Hepatectomy (PH) | Open field | PH 50 (Male) | Baseline | 3 | 42 | 63 | -0.21 | -0.60 | 0.18 |
| Partial Hepatectomy (PH) | Open field | PH 50 (Male) | Baseline | 4 | 21 | 63 | 0.17 | -0.32 | 0.66 |
| Partial Hepatectomy (PH) | Open field | PH 50 (Male) | Baseline | 7 | 21 | 63 | 1.08 | 0.56 | 1.60 |
| Partial Hepatectomy (PH) | Open field | PH 70 (Male) | Baseline | 1 | 58 | 63 | -1.27 | -1.66 | -0.88 |
| Partial Hepatectomy (PH) | Open field | PH 70 (Male) | Baseline | 3 | 37 | 63 | -0.93 | -1.35 | -0.51 |
| Partial Hepatectomy (PH) | Open field | PH 70 (Male) | Baseline | 4 | 18 | 63 | -0.54 | -1.06 | -0.01 |
| Partial Hepatectomy (PH) | Open field | PH 70 (Male) | Baseline | 7 | 18 | 63 | -0.36 | -0.88 | 0.17 |
| Partial Hepatectomy (PH) | Open field | PH 50 (Female) | Baseline | 1 | 7 | 7 | -1.84 | -3.03 | -0.59 |
| Partial Hepatectomy (PH) | Open field | PH 50 (Female) | Baseline | 3 | 7 | 7 | -0.27 | -1.25 | 0.73 |
| Partial Hepatectomy (PH) | Open field | PH 50 (Female) | Baseline | 4 | 7 | 7 | 0.04 | -0.95 | 1.02 |
| Partial Hepatectomy (PH) | Open field | PH 50 (Female) | Baseline | 7 | 7 | 7 | 0.31 | -0.68 | 1.29 |

*, Cohen’s H for Clinical Score and Neuroscore; Hedge’s G for Open field, VWR, and HC vm.

Experimental days refer to the days of and following the main surgical intervention.

For Clinical Score, days/comparisons without changes were not included in the table.

N, Non-steroidal anti-inflammatory drugs; L, Local anesthetic; O, Opioids; VWR, Voluntary wheel running; HC vm, Home-cage-based video monitoring.

Table S12. Effect size and confidence interval calculation in the “surgical interventions” category for the telemetry sub-analysis

| **Model ID** | **Readout parameter** | **Intervention group** | **Reference data** | **Experimental day(s)** | **Sample size** | | **Effect size*** | **Confidence interval** | |
| --- | --- | --- | --- | --- | --- | --- | --- | --- | --- |
|  |  |  |  |  | **Exp/Int** | **Reference** |  | **Lower** | **Higher** |
| Intraperitoneal Transmitter Implantation | Telemetry (light-off) | Carprofen | Baseline  (day 28) | 0 | 7 | 7 | -5.31 | -7.79 | -2.83 |
| Intraperitoneal Transmitter Implantation | Telemetry (light-off) | Carprofen | Baseline  (day 28) | 1 | 7 | 7 | -2.75 | -4.38 | -1.13 |
| Intraperitoneal Transmitter Implantation | Telemetry (light-off) | Carprofen | Baseline  (day 28) | 2 | 7 | 7 | -2.78 | -4.41 | -1.15 |
| Intraperitoneal Transmitter Implantation | Telemetry (light-off) | Carprofen | Baseline  (day 28) | 3 | 7 | 7 | -2.99 | -4.68 | -1.29 |
| Intraperitoneal Transmitter Implantation | Telemetry (light-off) | Carprofen | Baseline  (day 28) | 4 | 7 | 7 | -2.06 | -3.50 | -0.62 |
| Intraperitoneal Transmitter Implantation | Telemetry (light-off) | Carprofen | Baseline  (day 28) | 5 | 6 | 7 | -1.31 | -2.65 | 0.04 |
| Intraperitoneal Transmitter Implantation | Telemetry (light-off) | Carprofen | Baseline  (day 28) | 7 | 7 | 7 | -0.69 | -1.88 | 0.51 |
| Intraperitoneal Transmitter Implantation | Telemetry (light-off) | Carprofen | Baseline  (day 28) | 14 | 7 | 7 | 0.14 | -1.02 | 1.31 |
| Intraperitoneal Transmitter Implantation | Telemetry (light-off) | Metamizole | Baseline  (day 28) | 0 | 6 | 6 | -2.76 | -4.55 | -0.96 |
| Intraperitoneal Transmitter Implantation | Telemetry (light-off) | Metamizole | Baseline  (day 28) | 1 | 6 | 6 | -2.42 | -4.12 | -0.73 |
| Intraperitoneal Transmitter Implantation | Telemetry (light-off) | Metamizole | Baseline  (day 28) | 2 | 6 | 6 | -2.22 | -3.85 | -0.58 |
| Intraperitoneal Transmitter Implantation | Telemetry (light-off) | Metamizole | Baseline  (day 28) | 3 | 6 | 6 | -1.93 | -3.48 | -0.37 |
| Intraperitoneal Transmitter Implantation | Telemetry (light-off) | Metamizole | Baseline  (day 28) | 4 | 6 | 6 | -1.14 | -2.53 | 0.25 |
| Intraperitoneal Transmitter Implantation | Telemetry (light-off) | Metamizole | Baseline  (day 28) | 5 | 5 | 6 | -1.21 | -2.70 | 0.28 |
| Intraperitoneal Transmitter Implantation | Telemetry (light-off) | Metamizole | Baseline  (day 28) | 7 | 6 | 6 | -1.46 | -2.91 | -0.02 |
| Intraperitoneal Transmitter Implantation | Telemetry (light-off) | Metamizole | Baseline  (day 28) | 14 | 6 | 6 | -0.07 | -1.36 | 1.21 |
| Partial Hepatectomy (PH) | Clinical Score | 50 % PH | Baseline  (day -2) | 1 | 10 | 10 | 1.16 | 0.28 | 2.04 |
| Partial Hepatectomy (PH) | Clinical Score | 70 % PH | Baseline  (day -2) | 1 | 7 | 7 | 0.78 | -0.27 | 1.82 |
| Partial Hepatectomy (PH) | Clinical Score | 70 % PH | Baseline  (day -2) | 2 | 7 | 7 | 0.78 | -0.27 | 1.82 |
| Partial Hepatectomy (PH) | Telemetry (light-off) | 50 % PH | Baseline  (day -2) | 1 | 8 | 9 | -1.23 | -2.36 | -0.10 |
| Partial Hepatectomy (PH) | Telemetry (light-off) | 50 % PH | Baseline  (day -2) | 2 | 5 | 9 | / | / | / |
| Partial Hepatectomy (PH) | Telemetry (light-off) | 50 % PH | Baseline  (day -2) | 3 | 9 | 9 | -1.18 | -2.27 | -0.10 |
| Partial Hepatectomy (PH) | Telemetry (light-off) | 50 % PH | Baseline  (day -2) | 6 | 8 | 9 | 0.19 | -0.85 | 1.23 |
| Partial Hepatectomy (PH) | Telemetry (light-off) | 70 % PH | Baseline  (day -2) | 1 | 7 | 7 | -1.59 | -2.93 | -0.25 |
| Partial Hepatectomy (PH) | Telemetry (light-off) | 70 % PH | Baseline  (day -2) | 2 | 6 | 7 | -1.23 | -2.56 | 0.11 |
| Partial Hepatectomy (PH) | Telemetry (light-off) | 70 % PH | Baseline  (day -2) | 3 | 6 | 7 | -1.29 | -2.63 | 0.06 |
| Partial Hepatectomy (PH) | Telemetry (light-off) | 70 % PH | Baseline  (day -2) | 6 | 4 | 7 | / | / | / |
| Epilepsy Model - Post SE (IHK) | Clinical Score | IHK | Sham | 1 | 12 | 8 | 3.14 | 2.25 | 4.04 |
| Epilepsy Model - Post SE (IHK) | Clinical Score | IHK | Sham (tm) | 1 | 12 | 8 | 3.14 | 2.25 | 4.04 |
| Epilepsy Model - Post SE (IHK) | Telemetry (light-on) | IHK | Sham | 1 | 12 | 8 | 0.45 | -0.52 | 1.42 |
| Epilepsy Model - Post SE (IHK) | Telemetry (light-on) | IHK | Sham | 2 | 12 | 8 | -0.12 | -1.08 | 0.84 |
| Epilepsy Model - Post SE (IHK) | Telemetry (light-on) | IHK | Sham | 3 | 12 | 8 | 0.67 | -0.32 | 1.65 |
| Epilepsy Model - Post SE (IHK) | Telemetry (light-on) | IHK | Sham | 4 | 12 | 8 | 0.16 | -0.80 | 1.12 |
| Epilepsy Model - Post SE (IHK) | Telemetry (light-on) | IHK | Sham (tm) | 1 | 12 | 8 | 0.92 | -0.09 | 1.93 |
| Epilepsy Model - Post SE (IHK) | Telemetry (light-on) | IHK | Sham (tm) | 2 | 12 | 8 | -0.27 | -1.23 | 0.70 |
| Epilepsy Model - Post SE (IHK) | Telemetry (light-on) | IHK | Sham (tm) | 3 | 12 | 8 | 0.40 | -0.57 | 1.37 |
| Epilepsy Model - Post SE (IHK) | Telemetry (light-on) | IHK | Sham (tm) | 4 | 12 | 8 | 0.01 | -0.95 | 0.97 |
| Epilepsy Model - Post SE (IHK) | Telemetry (light-off) | IHK | Sham | 1 | 12 | 8 | -0.08 | -1.04 | 0.88 |
| Epilepsy Model - Post SE (IHK) | Telemetry (light-off) | IHK | Sham | 2 | 12 | 8 | 0.30 | -0.67 | 1.26 |
| Epilepsy Model - Post SE (IHK) | Telemetry (light-off) | IHK | Sham | 3 | 11 | 8 | 0.71 | -0.30 | 1.72 |
| Epilepsy Model - Post SE (IHK) | Telemetry (light-off) | IHK | Sham | 4 | 11 | 8 | 0.23 | -0.75 | 1.22 |
| Epilepsy Model - Post SE (IHK) | Telemetry (light-off) | IHK | Sham (tm) | 1 | 12 | 8 | -1.05 | -2.07 | -0.03 |
| Epilepsy Model - Post SE (IHK) | Telemetry (light-off) | IHK | Sham (tm) | 2 | 12 | 8 | -0.14 | -1.10 | 0.82 |
| Epilepsy Model - Post SE (IHK) | Telemetry (light-off) | IHK | Sham (tm) | 3 | 11 | 8 | -0.42 | -1.41 | 0.57 |
| Epilepsy Model - Post SE (IHK) | Telemetry (light-off) | IHK | Sham (tm) | 4 | 11 | 8 | 0.42 | -0.57 | 1.41 |
| Pancreatic Cancer Model | Clinical Score | Pancreatic cancer | Baseline  (day -1) | 0 | 9 | 10 | 1.68 | 0.78 | 2.58 |
| Pancreatic Cancer Model | Telemetry (light-off) | Pancreatic cancer | Baseline  (day -1) | 0 | 9 | 10 | -1.15 | -2.19 | -0.10 |
| Pancreatic Cancer Model | Telemetry (light-off) | Pancreatic cancer | Baseline  (day -1) | 1 | 9 | 10 | -0.15 | -1.12 | 0.82 |
| Pancreatic Cancer Model | Telemetry (light-off) | Pancreatic cancer | Baseline  (day -1) | 2 | 9 | 10 | 0.06 | -0.91 | 1.03 |
| Pancreatic Cancer Model | Telemetry (light-off) | Pancreatic cancer | Baseline  (day -1) | 3 | 9 | 10 | 0.16 | -0.81 | 1.13 |

*, Cohen’s H for Clinical Score and Hedge’s G for Telemetry.

Experimental days refer to the days of and following the main intervention of the model (e.g., transmitter implantation, partial hepatectomy, intracerebral electrode implantation, etc.).

For Clinical Score, days/comparisons without changes were not included in the table.

Baseline data includes data from the end of the laboratory-specific recovery phase (note that telemetric data can only be collected following surgery).

Exp/Int, Experimental/Intervention group; Sham (tm), transmitter only sham; /, no calculations for this time point due to over 20 % missing data; IHK, intrahippocampal kainate; SE, Status epilepticus*.*

Table S13. Effect size and confidence interval calculation in the "neuroscientific and stress tests/techniques" and the "genetic disease models" categories.

| **Model ID** | **Readout parameter** | **Experimental /Intervention group** | **Reference data** | **Experimental phase/day(s)** | **Sample size Exp/Int group** | **Sample size Reference data** | **Effect size** | **CI lower** | **CI higher** |
| --- | --- | --- | --- | --- | --- | --- | --- | --- | --- |
| Restraint Stress Model | Voluntary wheel running | Restraint stress | Naïve | -1 | 8 | 8 | 0.11 | -0.82 | 1.03 |
| Restraint Stress Model | Voluntary wheel running | Restraint stress | Naïve | 0 | 8 | 8 | 0.18 | -0.75 | 1.10 |
| Restraint Stress Model | Voluntary wheel running | Restraint stress | Naïve | 1 | 8 | 8 | -1.12 | -2.12 | -0.09 |
| Restraint Stress Model | Voluntary wheel running | Restraint stress | Naïve | 10 | 8 | 8 | -0.77 | -1.73 | 0.21 |
| Restraint Stress Model | Voluntary wheel running | Restraint stress | Naïve | 11 | 8 | 8 | -0.51 | -1.45 | 0.44 |
| Restraint Stress Model | Voluntary wheel running | Restraint stress | Naïve | 12 | 8 | 8 | -0.51 | -1.45 | 0.44 |
| Restraint Stress Model | Voluntary wheel running | Restraint stress | Naïve | 13 | 8 | 8 | -0.60 | -1.54 | 0.36 |
| Restraint Stress Model | Voluntary wheel running | Restraint stress | Naïve | 14 | 8 | 8 | -0.27 | -1.20 | 0.66 |
| Restraint Stress Model | Voluntary wheel running | Restraint stress | Naïve | 2 | 8 | 8 | -1.21 | -2.23 | -0.17 |
| Restraint Stress Model | Voluntary wheel running | Restraint stress | Naïve | 3 | 8 | 8 | -1.74 | -2.85 | -0.59 |
| Restraint Stress Model | Voluntary wheel running | Restraint stress | Naïve | 4 | 8 | 8 | -1.37 | -2.41 | -0.30 |
| Restraint Stress Model | Voluntary wheel running | Restraint stress | Naïve | 5 | 8 | 8 | -1.04 | -2.03 | -0.02 |
| Restraint Stress Model | Voluntary wheel running | Restraint stress | Naïve | 6 | 8 | 8 | -0.68 | -1.63 | 0.29 |
| Restraint Stress Model | Voluntary wheel running | Restraint stress | Naïve | 7 | 8 | 8 | -1.57 | -2.64 | -0.46 |
| Restraint Stress Model | Voluntary wheel running | Restraint stress | Naïve | 8 | 8 | 8 | -1.62 | -2.70 | -0.50 |
| Restraint Stress Model | Voluntary wheel running | Restraint stress | Naïve | 9 | 8 | 8 | -1.37 | -2.41 | -0.30 |
| Restraint Stress Model | Voluntary wheel running | Control | Baseline | 0 | 8 | 8 | -0.61 | -1.56 | 0.35 |
| Restraint Stress Model | Voluntary wheel running | Control | Baseline | 1 | 8 | 8 | -0.43 | -1.37 | 0.51 |
| Restraint Stress Model | Voluntary wheel running | Control | Baseline | 10 | 8 | 8 | -0.96 | -1.94 | 0.04 |
| Restraint Stress Model | Voluntary wheel running | Control | Baseline | 11 | 8 | 8 | -0.61 | -1.56 | 0.35 |
| Restraint Stress Model | Voluntary wheel running | Control | Baseline | 12 | 8 | 8 | -0.45 | -1.38 | 0.50 |
| Restraint Stress Model | Voluntary wheel running | Control | Baseline | 13 | 8 | 8 | -0.31 | -1.24 | 0.63 |
| Restraint Stress Model | Voluntary wheel running | Control | Baseline | 14 | 8 | 8 | -0.68 | -1.63 | 0.29 |
| Restraint Stress Model | Voluntary wheel running | Control | Baseline | 2 | 8 | 8 | -0.09 | -1.02 | 0.84 |
| Restraint Stress Model | Voluntary wheel running | Control | Baseline | 3 | 8 | 8 | 0.09 | -0.83 | 1.02 |
| Restraint Stress Model | Voluntary wheel running | Control | Baseline | 4 | 8 | 8 | -0.14 | -1.07 | 0.79 |
| Restraint Stress Model | Voluntary wheel running | Control | Baseline | 5 | 8 | 8 | -0.42 | -1.36 | 0.52 |
| Restraint Stress Model | Voluntary wheel running | Control | Baseline | 6 | 8 | 8 | -1.18 | -2.19 | -0.14 |
| Restraint Stress Model | Voluntary wheel running | Control | Baseline | 7 | 8 | 8 | -0.46 | -1.40 | 0.49 |
| Restraint Stress Model | Voluntary wheel running | Control | Baseline | 8 | 8 | 8 | -0.12 | -1.04 | 0.81 |
| Restraint Stress Model | Voluntary wheel running | Control | Baseline | 9 | 8 | 8 | -0.13 | -1.06 | 0.80 |
| Restraint Stress Model | Voluntary wheel running | Restraint stress | Baseline | 0 | 8 | 8 | -0.59 | -1.53 | 0.37 |
| Restraint Stress Model | Voluntary wheel running | Restraint stress | Baseline | 1 | 8 | 8 | -1.60 | -2.67 | -0.48 |
| Restraint Stress Model | Voluntary wheel running | Restraint stress | Baseline | 2 | 8 | 8 | -1.42 | -2.46 | -0.34 |
| Restraint Stress Model | Voluntary wheel running | Restraint stress | Baseline | 3 | 8 | 8 | -1.78 | -2.89 | -0.63 |
| Restraint Stress Model | Voluntary wheel running | Restraint stress | Baseline | 4 | 8 | 8 | -1.94 | -3.09 | -0.75 |
| Restraint Stress Model | Voluntary wheel running | Restraint stress | Baseline | 5 | 8 | 8 | -1.89 | -3.03 | -0.71 |
| Restraint Stress Model | Voluntary wheel running | Restraint stress | Baseline | 6 | 8 | 8 | -2.62 | -3.93 | -1.26 |
| Restraint Stress Model | Voluntary wheel running | Restraint stress | Baseline | 7 | 8 | 8 | -2.23 | -3.44 | -0.97 |
| Restraint Stress Model | Voluntary wheel running | Restraint stress | Baseline | 8 | 8 | 8 | -2.08 | -3.26 | -0.86 |
| Restraint Stress Model | Voluntary wheel running | Restraint stress | Baseline | 9 | 8 | 8 | -2.04 | -3.21 | -0.83 |
| Restraint Stress Model | Voluntary wheel running | Restraint stress | Baseline | 10 | 8 | 8 | -2.22 | -3.43 | -0.97 |
| Restraint Stress Model | Voluntary wheel running | Restraint stress | Baseline | 11 | 8 | 8 | -1.33 | -2.36 | -0.26 |
| Restraint Stress Model | Voluntary wheel running | Restraint stress | Baseline | 12 | 8 | 8 | -1.12 | -2.12 | -0.09 |
| Restraint Stress Model | Voluntary wheel running | Restraint stress | Baseline | 13 | 8 | 8 | -1.11 | -2.10 | -0.08 |
| Restraint Stress Model | Voluntary wheel running | Restraint stress | Baseline | 14 | 8 | 8 | -1.33 | -2.36 | -0.26 |
| Forced Swim Test (FST) | Open field | FST-Only | Naïve | Post-FST | 24 | 24 | -0.14 | -0.70 | 0.42 |
| Forced Swim Test (FST) | Open field | FST-Only | Naïve | Baseline | 24 | 24 | 0.12 | -0.44 | 0.68 |
| Forced Swim Test (FST) | Open field | FST-Only | Sham | Post-FST | 24 | 24 | 0.22 | -0.34 | 0.78 |
| Forced Swim Test (FST) | Open field | FST-Only | Sham | Baseline | 24 | 24 | 0.10 | -0.46 | 0.66 |
| Forced Swim Test (FST) | Open field | FST-Only | Baseline | Post-FST | 24 | 24 | -0.59 | -1.15 | -0.01 |
| Forced Swim Test (FST) | Open field | FST-Sal | Baseline | Post-FST | 24 | 24 | -0.48 | -1.04 | 0.09 |
| Forced Swim Test (FST) | Open field | Handling | Baseline | Post-FST | 24 | 24 | -0.20 | -0.75 | 0.36 |
| Dravet Syndrome Model | Neuroscore | *Scn1a* +/- (female) | Naïve (*Scn1a* WT) | prepub | 10 | 10 | 0.93 | 0.05 | 1.80 |
| Dravet Syndrome Model | Neuroscore | *Scn1a* +/- (female) | Naïve (*Scn1a* WT) | sexually_mat | 10 | 10 | 0.64 | -0.23 | 1.52 |
| Dravet Syndrome Model | Neuroscore | *Scn1a* +/- (male) | Naïve (*Scn1a* WT) | prepub | 10 | 10 | 1.16 | 0.28 | 2.04 |
| Dravet Syndrome Model | Neuroscore | *Scn1a* +/- (male) | Naïve (*Scn1a* WT) | sexually_mat | 10 | 10 | 0.93 | 0.05 | 1.80 |
| Dravet Syndrome Model | Open field | *Scn1a* +/- (female) | Naïve (*Scn1a* WT) | prepub | 10 | 10 | 2.41 | 1.25 | 3.54 |
| Dravet Syndrome Model | Open field | *Scn1a* +/- (female) | Naïve (*Scn1a* WT) | sexually_mat | 10 | 10 | 4.03 | 2.46 | 5.56 |
| Dravet Syndrome Model | Open field | *Scn1a* +/- (male) | Naïve (*Scn1a* WT) | prepub | 10 | 10 | 1.69 | 0.67 | 2.67 |
| Dravet Syndrome Model | Open field | *Scn1a* +/- (male) | Naïve (*Scn1a* WT) | sexually_mat | 10 | 10 | 3.10 | 1.77 | 4.39 |
| Dravet Syndrome Model | HC vm | *Scn1a* +/- (female) | Naïve (*Scn1a* WT) | prepub | 10 | 10 | 1.07 | 0.15 | 1.96 |
| Dravet Syndrome Model | HC vm | *Scn1a* +/- (female) | Naïve (*Scn1a* WT) | sexually_mat | 10 | 10 | -1.87 | -2.88 | -0.81 |
| Dravet Syndrome Model | HC vm | *Scn1a* +/- (male) | Naïve (*Scn1a* WT) | prepub | 10 | 10 | 0.12 | -0.72 | 0.96 |
| Dravet Syndrome Model | HC vm | *Scn1a* +/- (male) | Naïve (*Scn1a* WT) | sexually_mat | 10 | 10 | -1.43 | -2.37 | -0.46 |
| *Gria1* KO Model | Neuroscore | *Gria1* -/- (female) | Naïve (WT) | prepub | 10 | 10 | 0.93 | 0.05 | 1.80 |
| *Gria1* KO Model | Open field | *Gria1* -/- (female) | Naïve (WT) | prepub | 10 | 10 | 5.45 | 3.48 | 7.39 |
| *Gria1* KO Model | Open field | *Gria1* -/- (female) | Naïve (WT) | sexually_mat | 10 | 10 | 7.03 | 4.59 | 9.44 |
| *Gria1* KO Model | Open field | *Gria1* -/- (male) | Naïve (WT) | prepub | 8 | 10 | 3.37 | 1.90 | 4.81 |
| *Gria1* KO Model | Open field | *Gria1* -/- (male) | Naïve (WT) | sexually_mat | 8 | 10 | 3.82 | 2.22 | 5.38 |
| *Gria1* KO Model | Voluntary wheel running | *Gria1* -/- (female) | Naïve (WT) | prepub | 10 | 10 | -1.07 | -1.96 | -0.15 |
| *Gria1* KO Model | Voluntary wheel running | *Gria1* -/- (female) | Naïve (WT) | sexually_mat | 10 | 10 | -1.78 | -2.78 | -0.74 |
| *Gria1* KO Model | Voluntary wheel running | *Gria1* -/- (male) | Naïve (WT) | prepub | 8 | 10 | -0.83 | -1.75 | 0.11 |
| *Gria1* KO Model | Voluntary wheel running | *Gria1* -/- (male) | Naïve (WT) | sexually_mat | 8 | 10 | -0.29 | -1.17 | 0.61 |
| *Gria1* KO Model | HC vm | *Gria1* -/- (female) | Naïve (WT) | prepub | 10 | 10 | 0.26 | -0.58 | 1.10 |
| *Gria1* KO Model | HC vm | *Gria1* -/- (female) | Naïve (WT) | sexually_mat | 10 | 10 | 2.51 | 1.32 | 3.66 |
| *Gria1* KO Model | HC vm | *Gria1* -/- (male) | Naïve (WT) | prepub | 8 | 10 | 1.51 | 0.46 | 2.51 |
| *Gria1* KO Model | HC vm | *Gria1* -/- (male) | Naïve (WT) | sexually_mat | 8 | 10 | 1.00 | 0.03 | 1.93 |
| DAT KO Model | Neuroscore | homozygous | Naïve (WT) | sexual maturity | 16 | 20 | 1.32 | 0.66 | 1.98 |
| DAT KO Model | Open field | heterozygous | Naïve (WT) | sexual maturity | 20 | 20 | 0.13 | -0.48 | 0.73 |
| DAT KO Model | Open field | homozygous | Naïve (WT) | sexual maturity | 16 | 20 | 1.22 | 0.50 | 1.91 |

*, Cohen’s H for Clinical Score and Neuroscore; Hedge’s G for Open field, VWR, and HC vm.

Experimental days refer to the days of and following the main intervention in the model (e.g., stress induction) or the developmental stage of the animals.

For Clinical Score, days/comparisons without changes were not included in the table.

HC vm, Home-cage-based video monitoring; KO, Knock out; DAT, Dopamine transporter; WT, Wildtype

Table S14. Effect size and confidence interval calculation in the “neuroscientific and stress tests/techniques” category included in the telemetry sub-analysis.

| **Model ID** | **Readout parameter** | **Intervention group** | **Reference data** | **Experimental day(s)** | **Sample size** | | **Effect size*** | **Confidence interval** | |
| --- | --- | --- | --- | --- | --- | --- | --- | --- | --- |
|  |  |  |  |  | **Exp/ Int** | **Reference** |  | **Lower** | **Higher** |
| Biopotential Recording Techniques | Telemetry  (light-off) | Non-tethered | Baseline  (days -2 and -1) | 0 | 6 | 6 | -0.74 | -2.07 | 0.59 |
| Biopotential Recording Techniques | Telemetry  (light-off) | Non-tethered | Baseline  (days -2 and -1) | 1 | 6 | 6 | -0.71 | -2.04 | 0.61 |
| Biopotential Recording Techniques | Telemetry  (light-off) | Non-tethered | Baseline  (days -2 and -1) | 2 | 6 | 6 | -0.80 | -2.14 | 0.54 |
| Biopotential Recording Techniques | Telemetry  (light-off) | Non-tethered | Baseline  (days -2 and -1) | 5 | 6 | 6 | -0.42 | -1.72 | 0.88 |
| Biopotential Recording Techniques | Telemetry  (light-off) | Non-tethered | Baseline  (days -2 and -1) | 6 | 6 | 6 | -0.57 | -1.88 | 0.74 |
| Biopotential Recording Techniques | Telemetry  (light-off) | Non-tethered | Baseline  (days -2 and -1) | 7 | 6 | 6 | -0.56 | -1.88 | 0.75 |
| Biopotential Recording Techniques | Telemetry  (light-off) | Non-tethered | Baseline  (days -2 and -1) | 11 | 6 | 6 | -1.23 | -2.64 | 0.17 |
| Biopotential Recording Techniques | Telemetry  (light-off) | Non-tethered | Baseline  (days -2 and -1) | 12 | 6 | 6 | -1.18 | -2.57 | 0.22 |
| Biopotential Recording Techniques | Telemetry  (light-off) | Non-tethered | Baseline  (days -2 and -1) | 13 | 6 | 6 | -0.06 | -1.35 | 1.23 |
| Biopotential Recording Techniques | Telemetry  (light-off) | Tethered | Baseline  (days -2 and -1) | 0 | 7 | 7 | 0.14 | -1.02 | 1.31 |
| Biopotential Recording Techniques | Telemetry  (light-off) | Tethered | Baseline  (days -2 and -1) | 1 | 7 | 7 | 1.03 | -0.21 | 2.27 |
| Biopotential Recording Techniques | Telemetry  (light-off) | Tethered | Baseline  (days -2 and -1) | 2 | 7 | 7 | 0.02 | -1.14 | 1.19 |
| Biopotential Recording Techniques | Telemetry  (light-off) | Tethered | Baseline  (days -2 and -1) | 5 | 7 | 7 | 0.55 | -0.64 | 1.73 |
| Biopotential Recording Techniques | Telemetry  (light-off) | Tethered | Baseline  (days -2 and -1) | 6 | 7 | 7 | 0.62 | -0.57 | 1.82 |
| Biopotential Recording Techniques | Telemetry  (light-off) | Tethered | Baseline  (days -2 and -1) | 7 | 7 | 7 | 0.96 | -0.27 | 2.19 |
| Biopotential Recording Techniques | Telemetry  (light-off) | Tethered | Baseline  (days -2 and -1) | 11 | 7 | 7 | 0.58 | -0.60 | 1.77 |
| Biopotential Recording Techniques | Telemetry  (light-off) | Tethered | Baseline  (days -2 and -1) | 12 | 7 | 7 | 0.93 | -0.30 | 2.15 |
| Biopotential Recording Techniques | Telemetry  (light-off) | Tethered | Baseline  (days -2 and -1) | 13 | 7 | 7 | 1.10 | -0.15 | 2.35 |

*, Hedge’s G.

Experimental days refer to the days of and following the main intervention of the model (e.g., start of the tethered/non-tethered observation phase).

For Clinical Score, days/comparisons without changes were not included in the table.

Baseline data include data from the end of the laboratory-specific recovery phase (note that telemetric data can only be collected following surgery). Exp/Int, Experimental/Intervention group.

Table S15. Effect sizes and confidence intervals for the studies included in the “non-genetic disease models” category.

| **Model ID** | **Readout parameter** | **Experimental/ Intervention group** | **Reference data** | **Experimental phase/day(s)** | **Sample size Exp/Int group** | **Sample size Reference data** | **Effect size** | **CI lower** | **CI higher** |
| --- | --- | --- | --- | --- | --- | --- | --- | --- | --- |
| Epilepsy Model - Kindling (bla) | Neuroscore | BLA-Kindling | Naïve | Chronic phase | 14 | 15 | 1.57 | 0.84 | 2.30 |
| Epilepsy Model - Kindling (hip) | Neuroscore | Hip-Kindling | Naïve | Chronic phase | 11 | 15 | 1.48 | 0.70 | 2.26 |
| Epilepsy Model - Kindling (bla) | Neuroscore | BLA-Kindling | Sham | Chronic phase | 14 | 15 | 1.57 | 0.84 | 2.30 |
| Epilepsy Model - Kindling (hip) | Neuroscore | Hip-Kindling | Sham | Chronic phase | 11 | 15 | 1.48 | 0.70 | 2.26 |
| Epilepsy Model - Kindling (bla) | Open field | BLA-Kindling | Naïve | Chronic phase | 14 | 15 | -0.29 | -1.00 | 0.43 |
| Epilepsy Model - Kindling (hip) | Open field | Hip-Kindling | Naïve | Chronic phase | 11 | 15 | -0.17 | -0.92 | 0.59 |
| Epilepsy Model - Kindling (bla) | Open field | BLA-Kindling | Sham | Chronic phase | 14 | 15 | -1.33 | -2.11 | -0.53 |
| Epilepsy Model - Kindling (hip) | Open field | Hip-Kindling | Sham | Chronic phase | 11 | 15 | -0.83 | -1.62 | -0.04 |
| Epilepsy Model - Post-SE (IHK) | Neuroscore | IHK | Naïve | Chronic phase | 12 | 8 | 1.91 | 1.02 | 2.81 |
| Epilepsy Model - Post-SE (IHK) | Neuroscore | IHK | Sham | Chronic phase | 12 | 8 | 1.91 | 1.02 | 2.81 |
| Epilepsy Model - Post-SE (IHK) | Neuroscore | IHK | Sham (tm) | Chronic phase | 12 | 8 | 1.91 | 1.02 | 2.81 |
| Epilepsy Model - Post-SE (IHK) | Open field | IHK | Naïve | Chronic phase | 12 | 8 | -0.24 | -1.10 | 0.62 |
| Epilepsy Model - Post-SE (IHK) | Open field | IHK | Sham | Chronic phase | 12 | 8 | 0.21 | -0.65 | 1.07 |
| Epilepsy Model - Post-SE (IHK) | Open field | IHK | Sham (tm) | Chronic phase | 12 | 8 | -0.64 | -1.52 | 0.25 |
| Colitis Model (Lab 1) | Voluntary wheel running | 1% + phlebotomy | Sham | 1 | 14 | 8 | 0.31 | 1.15 | -0.53 |
| Colitis Model (Lab 1) | Voluntary wheel running | 1% + phlebotomy | Sham | 2 | 14 | 8 | 0.58 | 1.42 | -0.28 |
| Colitis Model (Lab 1) | Voluntary wheel running | 1% + phlebotomy | Sham | 3 | 14 | 8 | -0.55 | 0.31 | -1.39 |
| Colitis Model (Lab 1) | Voluntary wheel running | 1% + phlebotomy | Sham | 4 | 14 | 8 | -0.41 | 0.44 | -1.25 |
| Colitis Model (Lab 1) | Voluntary wheel running | 1% + phlebotomy | Sham | 5 | 14 | 8 | -0.41 | 0.44 | -1.25 |
| Colitis Model (Lab 1) | Voluntary wheel running | 1% + phlebotomy | Sham | 6 | 14 | 8 | -0.43 | 0.42 | -1.27 |
| Colitis Model (Lab 1) | Voluntary wheel running | 1% + phlebotomy | Sham | 7 | 13 | 8 | -0.97 | -0.06 | -1.86 |
| Colitis Model (Lab 1) | Voluntary wheel running | 1% + phlebotomy | Sham | 8 | 13 | 8 | -1.29 | -0.34 | -2.22 |
| Colitis Model (Lab 1) | Voluntary wheel running | 1% + phlebotomy | Sham | 9 | 11 | 8 | -1.71 | -0.65 | -2.73 |
| Colitis Model (Lab 1) | Voluntary wheel running | 1% + phlebotomy | Sham | 10 | 10 | 8 | -0.88 | 0.07 | -1.81 |
| Colitis Model (Lab 1) | Voluntary wheel running | 1% + phlebotomy | Sham | 11 | 10 | 8 | -0.95 | 0.01 | -1.88 |
| Colitis Model (Lab 1) | Voluntary wheel running | 1% + phlebotomy | Sham | 12 | 10 | 8 | -0.48 | 0.43 | -1.37 |
| Colitis Model (Lab 1) | Voluntary wheel running | 1% + phlebotomy | Sham | 13 | 10 | 8 | -0.58 | 0.34 | -1.48 |
| Colitis Model (Lab 1) | Voluntary wheel running | 1% + phlebotomy | Sham | 14 | 10 | 8 | -0.63 | 0.29 | -1.53 |
| Colitis Model (Lab 1) | Voluntary wheel running | 1,5% + phlebotomy | Sham | 1 | 9 | 8 | -0.20 | 0.71 | -1.10 |
| Colitis Model (Lab 1) | Voluntary wheel running | 1,5% + phlebotomy | Sham | 2 | 9 | 8 | -0.57 | 0.36 | -1.49 |
| Colitis Model (Lab 1) | Voluntary wheel running | 1,5% + phlebotomy | Sham | 3 | 9 | 8 | -0.96 | 0.02 | -1.91 |
| Colitis Model (Lab 1) | Voluntary wheel running | 1,5% + phlebotomy | Sham | 4 | 9 | 8 | -0.95 | 0.03 | -1.90 |
| Colitis Model (Lab 1) | Voluntary wheel running | 1,5% + phlebotomy | Sham | 5 | 9 | 8 | -0.97 | 0.01 | -1.92 |
| Colitis Model (Lab 1) | Voluntary wheel running | 1,5% + phlebotomy | Sham | 6 | 9 | 8 | -0.38 | 0.54 | -1.29 |
| Colitis Model (Lab 1) | Voluntary wheel running | 1,5% + phlebotomy | Sham | 7 | 9 | 8 | -1.94 | -0.78 | -3.06 |
| Colitis Model (Lab 1) | Voluntary wheel running | 1,5% + phlebotomy | Sham | 8 | 8 | 8 | -2.37 | -1.08 | -3.61 |
| Colitis Model (Lab 1) | Voluntary wheel running | 1,5% + phlebotomy | Sham | 9 | 7 | 8 | -2.18 | -0.89 | -3.42 |
| Colitis Model (Lab 1) | Voluntary wheel running | 1,5% + phlebotomy | Sham | 10 | 6 | 8 | -1.88 | -0.62 | -3.09 |
| Colitis Model (Lab 1) | Voluntary wheel running | 1,5% + phlebotomy | Sham | 11 | 6 | 8 | -1.63 | -0.43 | -2.79 |
| Colitis Model (Lab 1) | Voluntary wheel running | 1,5% + phlebotomy | Sham | 12 | 6 | 8 | -1.32 | -0.18 | -2.42 |
| Colitis Model (Lab 1) | Voluntary wheel running | 1,5% + phlebotomy | Sham | 13 | 6 | 8 | -1.33 | -0.19 | -2.43 |
| Colitis Model (Lab 1) | Voluntary wheel running | 1,5% + phlebotomy | Sham | 14 | 6 | 8 | -1.92 | -0.64 | -3.14 |
| Colitis Model (Lab 1) | Voluntary wheel running | 1% + handling | Sham | 1 | 8 | 7 | 0.44 | 1.40 | -0.54 |
| Colitis Model (Lab 1) | Voluntary wheel running | 1% + handling | Sham | 2 | 8 | 7 | 0.48 | 1.44 | -0.50 |
| Colitis Model (Lab 1) | Voluntary wheel running | 1% + handling | Sham | 3 | 8 | 7 | 0.40 | 1.36 | -0.57 |
| Colitis Model (Lab 1) | Voluntary wheel running | 1% + handling | Sham | 4 | 8 | 7 | 0.34 | 1.30 | -0.63 |
| Colitis Model (Lab 1) | Voluntary wheel running | 1% + handling | Sham | 5 | 8 | 7 | -0.37 | 0.60 | -1.33 |
| Colitis Model (Lab 1) | Voluntary wheel running | 1% + handling | Sham | 6 | 8 | 7 | -1.52 | -0.38 | -2.62 |
| Colitis Model (Lab 1) | Voluntary wheel running | 1% + handling | Sham | 7 | 8 | 7 | -1.01 | 0.04 | -2.02 |
| Colitis Model (Lab 1) | Voluntary wheel running | 1% + handling | Sham | 8 | 8 | 7 | -0.92 | 0.11 | -1.92 |
| Colitis Model (Lab 1) | Voluntary wheel running | 1% + handling | Sham | 9 | 8 | 7 | -0.86 | 0.17 | -1.85 |
| Colitis Model (Lab 1) | Voluntary wheel running | 1% + handling | Sham | 10 | 8 | 7 | -0.85 | 0.17 | -1.84 |
| Colitis Model (Lab 1) | Voluntary wheel running | 1% + handling | Sham | 11 | 8 | 7 | -0.74 | 0.27 | -1.72 |
| Colitis Model (Lab 1) | Voluntary wheel running | 1% + handling | Sham | 12 | 8 | 7 | -0.17 | 0.79 | -1.12 |
| Colitis Model (Lab 1) | Voluntary wheel running | 1% + handling | Sham | 13 | 8 | 6 | -0.69 | 0.35 | -1.71 |
| Colitis Model (Lab 1) | Voluntary wheel running | 1% + handling | Sham | 14 | 8 | 7 | 0.24 | 1.20 | -0.72 |
| Colitis Model (Lab 1) | Voluntary wheel running | 1,5% + handling | Sham | 1 | 8 | 7 | 1.10 | 2.12 | 0.04 |
| Colitis Model (Lab 1) | Voluntary wheel running | 1,5% + handling | Sham | 2 | 8 | 7 | 0.34 | 1.30 | -0.63 |
| Colitis Model (Lab 1) | Voluntary wheel running | 1,5% + handling | Sham | 3 | 8 | 7 | -0.33 | 0.64 | -1.29 |
| Colitis Model (Lab 1) | Voluntary wheel running | 1,5% + handling | Sham | 4 | 8 | 7 | -0.32 | 0.64 | -1.28 |
| Colitis Model (Lab 1) | Voluntary wheel running | 1,5% + handling | Sham | 5 | 8 | 7 | -0.86 | 0.16 | -1.85 |
| Colitis Model (Lab 1) | Voluntary wheel running | 1,5% + handling | Sham | 6 | 8 | 7 | -1.86 | -0.65 | -3.03 |
| Colitis Model (Lab 1) | Voluntary wheel running | 1,5% + handling | Sham | 7 | 8 | 7 | -2.71 | -1.28 | -4.09 |
| Colitis Model (Lab 1) | Voluntary wheel running | 1,5% + handling | Sham | 8 | 7 | 7 | -2.70 | -1.23 | -4.13 |
| Colitis Model (Lab 1) | Voluntary wheel running | 1,5% + handling | Sham | 9 | 7 | 7 | -1.92 | -0.66 | -3.14 |
| Colitis Model (Lab 1) | Voluntary wheel running | 1,5% + handling | Sham | 10 | 6 | 7 | -1.45 | -0.25 | -2.60 |
| Colitis Model (Lab 1) | Voluntary wheel running | 1,5% + handling | Sham | 11 | 6 | 7 | -1.82 | -0.53 | -3.06 |
| Colitis Model (Lab 1) | Voluntary wheel running | 1,5% + handling | Sham | 12 | 6 | 7 | -1.83 | -0.54 | -3.06 |
| Colitis Model (Lab 1) | Voluntary wheel running | 1,5% + handling | Sham | 13 | 6 | 6 | -0.93 | 0.21 | -2.03 |
| Colitis Model (Lab 1) | Voluntary wheel running | 1,5% + handling | Sham | 14 | 6 | 7 | -1.07 | 0.05 | -2.16 |
| Colitis Model (Lab 1) | Voluntary wheel running | 0% + phlebotomy | Baseline | 1 | 8 | 8 | -1.23 | -0.18 | -2.25 |
| Colitis Model (Lab 1) | Voluntary wheel running | 0% + phlebotomy | Baseline | 2 | 8 | 8 | -1.20 | -0.15 | -2.21 |
| Colitis Model (Lab 1) | Voluntary wheel running | 0% + phlebotomy | Baseline | 3 | 8 | 8 | -0.12 | 0.81 | -1.05 |
| Colitis Model (Lab 1) | Voluntary wheel running | 0% + phlebotomy | Baseline | 4 | 8 | 8 | -0.19 | 0.74 | -1.12 |
| Colitis Model (Lab 1) | Voluntary wheel running | 0% + phlebotomy | Baseline | 5 | 8 | 8 | -0.21 | 0.72 | -1.14 |
| Colitis Model (Lab 1) | Voluntary wheel running | 0% + phlebotomy | Baseline | 6 | 8 | 8 | -2.87 | -1.45 | -4.25 |
| Colitis Model (Lab 1) | Voluntary wheel running | 0% + phlebotomy | Baseline | 7 | 8 | 8 | -1.33 | -0.26 | -2.35 |
| Colitis Model (Lab 1) | Voluntary wheel running | 0% + phlebotomy | Baseline | 8 | 8 | 8 | -1.17 | -0.13 | -2.17 |
| Colitis Model (Lab 1) | Voluntary wheel running | 0% + phlebotomy | Baseline | 9 | 8 | 8 | -0.28 | 0.66 | -1.20 |
| Colitis Model (Lab 1) | Voluntary wheel running | 0% + phlebotomy | Baseline | 10 | 8 | 8 | -0.60 | 0.36 | -1.54 |
| Colitis Model (Lab 1) | Voluntary wheel running | 0% + phlebotomy | Baseline | 11 | 8 | 8 | -0.70 | 0.27 | -1.66 |
| Colitis Model (Lab 1) | Voluntary wheel running | 0% + phlebotomy | Baseline | 12 | 8 | 8 | -0.34 | 0.60 | -1.27 |
| Colitis Model (Lab 1) | Voluntary wheel running | 0% + phlebotomy | Baseline | 13 | 8 | 8 | -0.30 | 0.63 | -1.23 |
| Colitis Model (Lab 1) | Voluntary wheel running | 0% + phlebotomy | Baseline | 14 | 8 | 8 | 0.38 | 1.31 | -0.56 |
| Colitis Model (Lab 1) | Voluntary wheel running | 1% + phlebotomy | Baseline | 1 | 14 | 14 | -0.34 | 0.39 | -1.06 |
| Colitis Model (Lab 1) | Voluntary wheel running | 1% + phlebotomy | Baseline | 2 | 14 | 14 | -0.03 | 0.69 | -0.75 |
| Colitis Model (Lab 1) | Voluntary wheel running | 1% + phlebotomy | Baseline | 3 | 14 | 14 | -0.89 | -0.12 | -1.64 |
| Colitis Model (Lab 1) | Voluntary wheel running | 1% + phlebotomy | Baseline | 4 | 14 | 14 | -0.58 | 0.16 | -1.31 |
| Colitis Model (Lab 1) | Voluntary wheel running | 1% + phlebotomy | Baseline | 5 | 14 | 14 | -0.71 | 0.04 | -1.45 |
| Colitis Model (Lab 1) | Voluntary wheel running | 1% + phlebotomy | Baseline | 6 | 14 | 14 | -3.15 | -2.02 | -4.25 |
| Colitis Model (Lab 1) | Voluntary wheel running | 1% + phlebotomy | Baseline | 7 | 13 | 14 | -2.37 | -1.37 | -3.33 |
| Colitis Model (Lab 1) | Voluntary wheel running | 1% + phlebotomy | Baseline | 8 | 13 | 14 | -2.44 | -1.43 | -3.42 |
| Colitis Model (Lab 1) | Voluntary wheel running | 1% + phlebotomy | Baseline | 9 | 11 | 14 | -2.89 | -1.75 | -4.01 |
| Colitis Model (Lab 1) | Voluntary wheel running | 1% + phlebotomy | Baseline | 10 | 10 | 14 | -1.74 | -0.79 | -2.66 |
| Colitis Model (Lab 1) | Voluntary wheel running | 1% + phlebotomy | Baseline | 11 | 10 | 14 | -2.24 | -1.20 | -3.25 |
| Colitis Model (Lab 1) | Voluntary wheel running | 1% + phlebotomy | Baseline | 12 | 10 | 14 | -1.03 | -0.18 | -1.86 |
| Colitis Model (Lab 1) | Voluntary wheel running | 1% + phlebotomy | Baseline | 13 | 10 | 14 | -1.63 | -0.70 | -2.54 |
| Colitis Model (Lab 1) | Voluntary wheel running | 1% + phlebotomy | Baseline | 14 | 10 | 14 | -0.56 | 0.25 | -1.36 |
| Colitis Model (Lab 1) | Voluntary wheel running | 1,5% + phlebotomy | Baseline | 1 | 9 | 9 | -1.82 | -0.72 | -2.88 |
| Colitis Model (Lab 1) | Voluntary wheel running | 1,5% + phlebotomy | Baseline | 2 | 9 | 9 | -2.50 | -1.25 | -3.71 |
| Colitis Model (Lab 1) | Voluntary wheel running | 1,5% + phlebotomy | Baseline | 3 | 9 | 9 | -1.69 | -0.62 | -2.73 |
| Colitis Model (Lab 1) | Voluntary wheel running | 1,5% + phlebotomy | Baseline | 4 | 9 | 9 | -1.29 | -0.29 | -2.26 |
| Colitis Model (Lab 1) | Voluntary wheel running | 1,5% + phlebotomy | Baseline | 5 | 9 | 9 | -1.86 | -0.75 | -2.93 |
| Colitis Model (Lab 1) | Voluntary wheel running | 1,5% + phlebotomy | Baseline | 6 | 9 | 9 | -3.37 | -1.90 | -4.80 |
| Colitis Model (Lab 1) | Voluntary wheel running | 1,5% + phlebotomy | Baseline | 7 | 9 | 9 | -5.49 | -3.40 | -7.56 |
| Colitis Model (Lab 1) | Voluntary wheel running | 1,5% + phlebotomy | Baseline | 8 | 8 | 9 | -4.68 | -2.77 | -6.55 |
| Colitis Model (Lab 1) | Voluntary wheel running | 1,5% + phlebotomy | Baseline | 9 | 7 | 9 | -4.39 | -2.51 | -6.23 |
| Colitis Model (Lab 1) | Voluntary wheel running | 1,5% + phlebotomy | Baseline | 10 | 6 | 9 | -5.48 | -3.16 | -7.75 |
| Colitis Model (Lab 1) | Voluntary wheel running | 1,5% + phlebotomy | Baseline | 11 | 6 | 9 | -4.74 | -2.68 | -6.77 |
| Colitis Model (Lab 1) | Voluntary wheel running | 1,5% + phlebotomy | Baseline | 12 | 6 | 9 | -3.66 | -1.94 | -5.33 |
| Colitis Model (Lab 1) | Voluntary wheel running | 1,5% + phlebotomy | Baseline | 13 | 6 | 9 | -6.11 | -3.58 | -8.61 |
| Colitis Model (Lab 1) | Voluntary wheel running | 1,5% + phlebotomy | Baseline | 14 | 6 | 9 | -3.05 | -1.51 | -4.54 |
| Colitis Model (Lab 1) | Voluntary wheel running | 0% + handling | Baseline | 1 | 7 | 7 | -1.64 | -0.44 | -2.79 |
| Colitis Model (Lab 1) | Voluntary wheel running | 0% + handling | Baseline | 2 | 7 | 7 | -0.43 | 0.57 | -1.42 |
| Colitis Model (Lab 1) | Voluntary wheel running | 0% + handling | Baseline | 3 | 7 | 7 | -0.24 | 0.75 | -1.22 |
| Colitis Model (Lab 1) | Voluntary wheel running | 0% + handling | Baseline | 4 | 7 | 7 | -0.89 | 0.17 | -1.91 |
| Colitis Model (Lab 1) | Voluntary wheel running | 0% + handling | Baseline | 5 | 7 | 7 | -0.63 | 0.39 | -1.63 |
| Colitis Model (Lab 1) | Voluntary wheel running | 0% + handling | Baseline | 6 | 7 | 7 | -0.86 | 0.20 | -1.88 |
| Colitis Model (Lab 1) | Voluntary wheel running | 0% + handling | Baseline | 7 | 7 | 7 | -0.05 | 0.93 | -1.03 |
| Colitis Model (Lab 1) | Voluntary wheel running | 0% + handling | Baseline | 8 | 7 | 7 | 0.47 | 1.46 | -0.54 |
| Colitis Model (Lab 1) | Voluntary wheel running | 0% + handling | Baseline | 9 | 7 | 7 | -0.49 | 0.52 | -1.48 |
| Colitis Model (Lab 1) | Voluntary wheel running | 0% + handling | Baseline | 10 | 7 | 7 | -1.27 | -0.15 | -2.35 |
| Colitis Model (Lab 1) | Voluntary wheel running | 0% + handling | Baseline | 11 | 7 | 7 | -0.68 | 0.35 | -1.68 |
| Colitis Model (Lab 1) | Voluntary wheel running | 0% + handling | Baseline | 12 | 7 | 7 | 0.47 | 1.46 | -0.54 |
| Colitis Model (Lab 1) | Voluntary wheel running | 0% + handling | Baseline | 14 | 7 | 7 | -0.47 | 0.53 | -1.46 |
| Colitis Model (Lab 1) | Voluntary wheel running | 0% + handling | Baseline | 13 | 6 | 7 | -0.22 | 0.80 | -1.23 |
| Colitis Model (Lab 1) | Voluntary wheel running | 1% + handling | Baseline | 1 | 8 | 8 | -0.60 | 0.36 | -1.55 |
| Colitis Model (Lab 1) | Voluntary wheel running | 1% + handling | Baseline | 2 | 8 | 8 | 0.23 | 1.15 | -0.71 |
| Colitis Model (Lab 1) | Voluntary wheel running | 1% + handling | Baseline | 3 | 8 | 8 | 0.33 | 1.26 | -0.61 |
| Colitis Model (Lab 1) | Voluntary wheel running | 1% + handling | Baseline | 4 | 8 | 8 | -0.23 | 0.71 | -1.15 |
| Colitis Model (Lab 1) | Voluntary wheel running | 1% + handling | Baseline | 5 | 8 | 8 | -1.21 | -0.16 | -2.22 |
| Colitis Model (Lab 1) | Voluntary wheel running | 1% + handling | Baseline | 6 | 8 | 8 | -2.98 | -1.52 | -4.39 |
| Colitis Model (Lab 1) | Voluntary wheel running | 1% + handling | Baseline | 7 | 8 | 8 | -1.22 | -0.17 | -2.23 |
| Colitis Model (Lab 1) | Voluntary wheel running | 1% + handling | Baseline | 8 | 8 | 8 | -0.86 | 0.14 | -1.82 |
| Colitis Model (Lab 1) | Voluntary wheel running | 1% + handling | Baseline | 9 | 8 | 8 | -1.23 | -0.18 | -2.24 |
| Colitis Model (Lab 1) | Voluntary wheel running | 1% + handling | Baseline | 10 | 8 | 8 | -1.46 | -0.37 | -2.51 |
| Colitis Model (Lab 1) | Voluntary wheel running | 1% + handling | Baseline | 11 | 8 | 8 | -1.20 | -0.16 | -2.21 |
| Colitis Model (Lab 1) | Voluntary wheel running | 1% + handling | Baseline | 12 | 8 | 8 | 0.07 | 1.00 | -0.86 |
| Colitis Model (Lab 1) | Voluntary wheel running | 1% + handling | Baseline | 13 | 8 | 8 | -1.23 | -0.18 | -2.24 |
| Colitis Model (Lab 1) | Voluntary wheel running | 1% + handling | Baseline | 14 | 8 | 8 | 0.01 | 0.93 | -0.92 |
| Colitis Model (Lab 1) | Voluntary wheel running | 1,5% + handling | Baseline | 1 | 8 | 8 | -0.67 | 0.30 | -1.61 |
| Colitis Model (Lab 1) | Voluntary wheel running | 1,5% + handling | Baseline | 2 | 8 | 8 | -0.03 | 0.90 | -0.96 |
| Colitis Model (Lab 1) | Voluntary wheel running | 1,5% + handling | Baseline | 3 | 8 | 8 | -0.79 | 0.19 | -1.75 |
| Colitis Model (Lab 1) | Voluntary wheel running | 1,5% + handling | Baseline | 4 | 8 | 8 | -0.86 | 0.13 | -1.83 |
| Colitis Model (Lab 1) | Voluntary wheel running | 1,5% + handling | Baseline | 5 | 8 | 8 | -1.83 | -0.67 | -2.96 |
| Colitis Model (Lab 1) | Voluntary wheel running | 1,5% + handling | Baseline | 6 | 8 | 8 | -2.99 | -1.53 | -4.40 |
| Colitis Model (Lab 1) | Voluntary wheel running | 1,5% + handling | Baseline | 7 | 8 | 8 | -3.16 | -1.65 | -4.62 |
| Colitis Model (Lab 1) | Voluntary wheel running | 1,5% + handling | Baseline | 8 | 7 | 8 | -2.81 | -1.35 | -4.22 |
| Colitis Model (Lab 1) | Voluntary wheel running | 1,5% + handling | Baseline | 9 | 7 | 8 | -2.36 | -1.03 | -3.65 |
| Colitis Model (Lab 1) | Voluntary wheel running | 1,5% + handling | Baseline | 10 | 6 | 8 | -1.86 | -0.60 | -3.07 |
| Colitis Model (Lab 1) | Voluntary wheel running | 1,5% + handling | Baseline | 11 | 6 | 8 | -2.32 | -0.94 | -3.64 |
| Colitis Model (Lab 1) | Voluntary wheel running | 1,5% + handling | Baseline | 12 | 6 | 8 | -1.96 | -0.68 | -3.19 |
| Colitis Model (Lab 1) | Voluntary wheel running | 1,5% + handling | Baseline | 13 | 6 | 8 | -1.25 | -0.12 | -2.33 |
| Colitis Model (Lab 1) | Voluntary wheel running | 1,5% + handling | Baseline | 14 | 6 | 8 | -1.81 | -0.56 | -3.00 |
| Colitis Model (Lab 2) | Clinical Score | DSS | Baseline | 9 | 9 | 9 | 1.68 | 0.76 | 2.61 |
| Colitis Model (Lab 2) | Clinical Score | DSS | Baseline | 10 | 9 | 9 | 1.46 | 0.54 | 2.38 |
| Colitis Model (Lab 2) | Clinical Score | DSS | Baseline | 2 | 9 | 9 | 0.98 | 0.06 | 1.91 |
| Colitis Model (Lab 2) | Clinical Score | DSS | Baseline | 8 | 9 | 9 | 1.91 | 0.99 | 2.83 |
| Colitis Model (Lab 2) | Clinical Score | DSS | Baseline | 11 | 9 | 9 | 1.23 | 0.31 | 2.15 |
| Colitis Model (Lab 2) | Clinical Score | DSS | Baseline | 12 | 9 | 9 | 0.98 | 0.06 | 1.91 |
| Colitis Model (Lab 2) | Clinical Score | DSS | Baseline | 15 | 9 | 9 | 1.23 | 0.31 | 2.15 |
| Colitis Model (Lab 2) | Clinical Score | DSS | Baseline | 7 | 9 | 9 | 1.46 | 0.54 | 2.38 |
| Colitis Model (Lab 2) | Clinical Score | DSS | Baseline | 5 | 9 | 9 | 0.68 | -0.24 | 1.60 |
| Colitis Model (Lab 2) | Voluntary wheel running | DSS | Baseline | 1 | 9 | 9 | -0.10 | 0.78 | -0.98 |
| Colitis Model (Lab 2) | Voluntary wheel running | DSS | Baseline | 2 | 9 | 9 | -0.68 | 0.24 | -1.58 |
| Colitis Model (Lab 2) | Voluntary wheel running | DSS | Baseline | 3 | 9 | 9 | -0.48 | 0.42 | -1.37 |
| Colitis Model (Lab 2) | Voluntary wheel running | DSS | Baseline | 4 | 9 | 9 | -0.92 | 0.03 | -1.84 |
| Colitis Model (Lab 2) | Voluntary wheel running | DSS | Baseline | 5 | 9 | 9 | -0.76 | 0.17 | -1.67 |
| Colitis Model (Lab 2) | Voluntary wheel running | DSS | Baseline | 6 | 9 | 9 | -1.17 | -0.19 | -2.12 |
| Colitis Model (Lab 2) | Voluntary wheel running | DSS | Baseline | 7 | 9 | 9 | -1.78 | -0.69 | -2.84 |
| Colitis Model (Lab 2) | Voluntary wheel running | DSS | Baseline | 8 | 9 | 9 | -2.99 | -1.62 | -4.32 |
| Colitis Model (Lab 2) | Voluntary wheel running | DSS | Baseline | 9 | 9 | 9 | -2.08 | -0.93 | -3.20 |
| Colitis Model (Lab 2) | Voluntary wheel running | DSS | Baseline | 10 | 9 | 9 | -1.76 | -0.68 | -2.81 |
| Colitis Model (Lab 2) | Voluntary wheel running | DSS | Baseline | 11 | 9 | 9 | -1.55 | -0.51 | -2.56 |
| Colitis Model (Lab 2) | Voluntary wheel running | DSS | Baseline | 12 | 9 | 9 | -1.00 | -0.04 | -1.93 |
| Colitis Model (Lab 2) | Voluntary wheel running | DSS | Baseline | 15 | 9 | 9 | -0.86 | 0.08 | -1.77 |
| Colitis Model (Lab 2) | Voluntary wheel running | DSS | Baseline | 16 | 9 | 9 | -0.63 | 0.29 | -1.52 |
| Epilepsy Model - Kindling (teth) | Open field | kindling | Naïve | Chronic (Generalized) | 11 | 12 | -0.09 | 0.70 | -0.88 |
| Epilepsy Model - Kindling (teth) | Open field | kindling | Sham | Chronic (Generalized) | 11 | 12 | -0.07 | 0.72 | -0.85 |
| Epilepsy Model - Post SE (pilo: PET) | Open field | Pilo | Naïve | Chronic | 13 | 12 | 0.04 | -0.73 | 0.81 |
| Epilepsy Model - Post SE (pilo: PET) | Open field | Pilo | Sham | Chronic | 13 | 12 | 0.11 | -0.68 | 0.90 |
| Epilepsy Model - Post SE (pilo: tele) | Open field | SE | Sham | Chronic | 22 | 12 | 0.20 | -0.76 | 1.15 |
| Epilepsy Model - Post SE (pilo: teth) | Open field | SE_Pilo | Naïve | Chronic | 15 | 12 | 0.51 | -0.26 | 1.26 |
| Epilepsy Model - Post SE (pilo: teth) | Open field | SE_Pilo | Sham | Chronic | 15 | 12 | 0.63 | -0.14 | 1.39 |
| Epilepsy Model - Post SE (electrical: teth) | Clinical Score | SE | Naïve | Chronic | 10 | 11 | 0.64 | -0.21 | 1.50 |
| Epilepsy Model - Post SE (electrical: teth) | Clinical Score | SE | Sham | Chronic | 10 | 11 | 0.64 | -0.21 | 1.50 |
| Epilepsy Model - Post SE (electrical: tele) | Neuroscore | SE | Sham | Chronic | 14 | 12 | 1.71 | 0.62 | 2.80 |
| Epilepsy Model - Post SE (electrical: teth) | Neuroscore | SE | Naïve | Chronic | 10 | 11 | 1.16 | 0.30 | 2.02 |
| Epilepsy Model - Post SE (electrical: teth) | Neuroscore | SE | Sham | Chronic | 10 | 11 | 1.16 | 0.30 | 2.02 |
| Epilepsy Model - Post SE (electrical: PET) | Open field | SE | Naïve | Chronic | 9 | 12 | 2.02 | 0.96 | 3.06 |
| Epilepsy Model - Post SE (electrical: PET) | Open field | SE | Sham | Chronic | 9 | 8 | 1.84 | 0.70 | 2.93 |
| Epilepsy Model - Post SE (electrical: tele) | Open field | SE | Sham | Chronic | 14 | 12 | -0.31 | -1.32 | 0.72 |
| Epilepsy Model - Post SE (electrical: teth) | Open field | SE | Naïve | Chronic | 10 | 11 | 0.74 | -0.12 | 1.59 |
| Epilepsy Model - Post SE (electrical: teth) | Open field | SE | Sham | Chronic | 10 | 11 | 1.08 | 0.18 | 1.96 |
| Hearing Loss Model (Juvenile) | Open field | deaf | Naïve | 1 | 12 | 8 | 0.23 | -0.63 | 1.09 |
| Hearing Loss Model (Juvenile) | Open field | deaf | Naïve | 2 | 12 | 8 | 0.30 | -0.57 | 1.16 |
| Hearing Loss Model (Juvenile) | Open field | deaf | Naïve | 4 | 12 | 8 | 0.49 | -0.38 | 1.36 |
| Hearing Loss Model (Juvenile) | Open field | deaf | Naïve | 8 | 12 | 8 | 1.18 | 0.23 | 2.10 |
| Hearing Loss Model (Juvenile) | Open field | deaf | Naïve | 16 | 12 | 8 | 0.86 | -0.05 | 1.75 |
| Hearing Loss Model (Juvenile) | Open field | deaf | Naïve | 24 | 12 | 8 | 0.94 | 0.02 | 1.84 |
| Hearing Loss Model (Juvenile) | Open field | deaf | Sham | 1 | 12 | 8 | 0.36 | -0.51 | 1.22 |
| Hearing Loss Model (Juvenile) | Open field | deaf | Sham | 2 | 12 | 8 | 0.34 | -0.53 | 1.20 |
| Hearing Loss Model (Juvenile) | Open field | deaf | Sham | 4 | 12 | 8 | 0.81 | -0.10 | 1.70 |
| Hearing Loss Model (Juvenile) | Open field | deaf | Sham | 8 | 12 | 8 | 1.22 | 0.26 | 2.15 |
| Hearing Loss Model (Juvenile) | Open field | deaf | Sham | 16 | 12 | 8 | 0.84 | -0.07 | 1.72 |
| Hearing Loss Model (Juvenile) | Open field | deaf | Sham | 24 | 12 | 8 | 1.04 | 0.11 | 1.95 |
| Hearing Loss Model (Adult 2nd cohort) | Open field | deaf | Naïve | 0 | 12 | 9 | 0.12 | -0.77 | 1.01 |
| Hearing Loss Model (Adult 2nd cohort) | Open field | deaf | Naïve | 1 | 10 | 8 | 0.26 | -0.64 | 1.14 |
| Hearing Loss Model (Adult 2nd cohort) | Open field | deaf | Naïve | 2 | 12 | 9 | 0.16 | -0.67 | 0.99 |
| Hearing Loss Model (Adult 2nd cohort) | Open field | deaf | Naïve | 4 | 12 | 9 | 0.48 | -0.37 | 1.32 |
| Hearing Loss Model (Adult 2nd cohort) | Open field | deaf | Naïve | 8 | 12 | 9 | 0.27 | -0.57 | 1.10 |
| Hearing Loss Model (Adult 2nd cohort) | Open field | deaf | Naïve | 16 | 12 | 9 | -0.51 | -1.37 | 0.37 |
| Hearing Loss Model (Adult 2nd cohort) | Open field | deaf | Naïve | 24 | 12 | 9 | 0.00 | -0.83 | 0.83 |
| Hearing Loss Model (Adult 2nd cohort) | Open field | deaf | Naïve | 32 | 12 | 9 | 0.23 | -0.62 | 1.08 |
| Hearing Loss Model (Adult 2nd cohort) | Open field | deaf | Naïve | 40 | 12 | 9 | -0.41 | -1.28 | 0.48 |
| Hearing Loss Model (Adult 2nd cohort) | Open field | deaf | Naïve | 48 | 12 | 9 | -0.71 | -1.62 | 0.22 |
| Hearing Loss Model (Adult 2nd cohort) | Open field | deaf | Naïve | 56 | 12 | 9 | -0.78 | -1.69 | 0.16 |
| Hearing Loss Model (Adult 2nd cohort) | Open field | deaf | Sham | 0 | 12 | 4 | -0.18 | -1.27 | 0.91 |
| Hearing Loss Model (Adult 2nd cohort) | Open field | deaf | Sham | 1 | 10 | 4 | 0.07 | -1.02 | 1.15 |
| Hearing Loss Model (Adult 2nd cohort) | Open field | deaf | Sham | 2 | 12 | 4 | -0.15 | -1.22 | 0.92 |
| Hearing Loss Model (Adult 2nd cohort) | Open field | deaf | Sham | 4 | 12 | 4 | 0.02 | -1.05 | 1.09 |
| Hearing Loss Model (Adult 2nd cohort) | Open field | deaf | Sham | 8 | 12 | 4 | 0.24 | -0.84 | 1.31 |
| Hearing Loss Model (Adult 2nd cohort) | Open field | deaf | Sham | 16 | 12 | 4 | -0.01 | -1.07 | 1.06 |
| Hearing Loss Model (Adult 2nd cohort) | Open field | deaf | Sham | 24 | 12 | 4 | 0.22 | -0.86 | 1.29 |
| Hearing Loss Model (Adult 2nd cohort) | Open field | deaf | Sham | 32 | 12 | 4 | 0.69 | -0.43 | 1.79 |
| Hearing Loss Model (Adult 2nd cohort) | Open field | deaf | Sham | 40 | 12 | 4 | 0.11 | -0.97 | 1.18 |
| Hearing Loss Model (Adult 2nd cohort) | Open field | deaf | Sham | 48 | 12 | 4 | -0.12 | -1.20 | 0.97 |
| Hearing Loss Model (Adult 2nd cohort) | Open field | deaf | Sham | 56 | 12 | 4 | 0.04 | -1.05 | 1.13 |
| Hearing Loss Model (Adult 1st cohort) | Open field | deaf | Naïve | 0 | 11 | 10 | 0.28 | -0.55 | 1.10 |
| Hearing Loss Model (Adult 1st cohort) | Open field | deaf | Naïve | 1 | 11 | 10 | 0.97 | 0.08 | 1.84 |
| Hearing Loss Model (Adult 1st cohort) | Open field | deaf | Naïve | 2 | 11 | 10 | 1.12 | 0.21 | 2.00 |
| Hearing Loss Model (Adult 1st cohort) | Open field | deaf | Naïve | 4 | 11 | 10 | 1.31 | 0.38 | 2.22 |
| Hearing Loss Model (Adult 1st cohort) | Open field | deaf | Naïve | 8 | 11 | 10 | 1.14 | 0.23 | 2.03 |
| Hearing Loss Model (Adult 1st cohort) | Open field | deaf | Naïve | 16 | 11 | 10 | 1.18 | 0.27 | 2.07 |
| Hearing Loss Model (Adult 1st cohort) | Open field | deaf | Naïve | 24 | 11 | 10 | 0.99 | 0.08 | 1.88 |
| Hearing Loss Model (Adult 2nd cohort) | Open field | deaf | Baseline | 1 | 10 | 12 | 0.30 | -0.55 | 1.14 |
| Hearing Loss Model (Adult 2nd cohort) | Open field | deaf | Baseline | 2 | 12 | 12 | 0.38 | -0.44 | 1.19 |
| Hearing Loss Model (Adult 2nd cohort) | Open field | deaf | Baseline | 4 | 12 | 12 | 0.54 | -0.29 | 1.36 |
| Hearing Loss Model (Adult 2nd cohort) | Open field | deaf | Baseline | 8 | 12 | 12 | 0.46 | -0.36 | 1.28 |
| Hearing Loss Model (Adult 2nd cohort) | Open field | deaf | Baseline | 16 | 12 | 12 | 0.25 | -0.56 | 1.06 |
| Hearing Loss Model (Adult 2nd cohort) | Open field | deaf | Baseline | 24 | 12 | 12 | 0.47 | -0.36 | 1.28 |
| Hearing Loss Model (Adult 2nd cohort) | Open field | deaf | Baseline | 32 | 12 | 12 | 0.88 | 0.00 | 1.73 |
| Hearing Loss Model (Adult 2nd cohort) | Open field | deaf | Baseline | 40 | 12 | 12 | 0.13 | -0.69 | 0.95 |
| Hearing Loss Model (Adult 2nd cohort) | Open field | deaf | Baseline | 48 | 12 | 12 | -0.08 | -0.92 | 0.76 |
| Hearing Loss Model (Adult 2nd cohort) | Open field | deaf | Baseline | 56 | 12 | 12 | -0.01 | -0.85 | 0.83 |
| Hearing Loss Model (Adult 2nd cohort) | Open field | naive | Baseline | 1 | 8 | 9 | 0.16 | -0.77 | 1.08 |
| Hearing Loss Model (Adult 2nd cohort) | Open field | naive | Baseline | 2 | 9 | 9 | 0.25 | -0.66 | 1.15 |
| Hearing Loss Model (Adult 2nd cohort) | Open field | naive | Baseline | 4 | 9 | 9 | 0.18 | -0.73 | 1.08 |
| Hearing Loss Model (Adult 2nd cohort) | Open field | naive | Baseline | 8 | 9 | 9 | 0.36 | -0.56 | 1.27 |
| Hearing Loss Model (Adult 2nd cohort) | Open field | naive | Baseline | 16 | 9 | 9 | 0.94 | -0.07 | 1.91 |
| Hearing Loss Model (Adult 2nd cohort) | Open field | naive | Baseline | 24 | 9 | 9 | 0.54 | -0.39 | 1.45 |
| Hearing Loss Model (Adult 2nd cohort) | Open field | naive | Baseline | 32 | 9 | 9 | 0.50 | -0.43 | 1.42 |
| Hearing Loss Model (Adult 2nd cohort) | Open field | naive | Baseline | 40 | 9 | 9 | 0.55 | -0.40 | 1.49 |
| Hearing Loss Model (Adult 2nd cohort) | Open field | naive | Baseline | 48 | 9 | 9 | 0.68 | -0.29 | 1.63 |
| Hearing Loss Model (Adult 2nd cohort) | Open field | naive | Baseline | 56 | 9 | 9 | 0.85 | -0.14 | 1.82 |
| Hearing Loss Model (Adult 2nd cohort) | Open field | sham | Baseline | 1 | 4 | 4 | 0.08 | -1.13 | 1.28 |
| Hearing Loss Model (Adult 2nd cohort) | Open field | sham | Baseline | 2 | 4 | 4 | 0.38 | -0.85 | 1.59 |
| Hearing Loss Model (Adult 2nd cohort) | Open field | sham | Baseline | 4 | 4 | 4 | 0.42 | -0.82 | 1.63 |
| Hearing Loss Model (Adult 2nd cohort) | Open field | sham | Baseline | 8 | 4 | 4 | 0.11 | -1.10 | 1.31 |
| Hearing Loss Model (Adult 2nd cohort) | Open field | sham | Baseline | 16 | 4 | 4 | 0.18 | -1.03 | 1.38 |
| Hearing Loss Model (Adult 2nd cohort) | Open field | sham | Baseline | 24 | 4 | 4 | 0.13 | -1.08 | 1.33 |
| Hearing Loss Model (Adult 2nd cohort) | Open field | sham | Baseline | 32 | 4 | 4 | -0.01 | -1.21 | 1.20 |
| Hearing Loss Model (Adult 2nd cohort) | Open field | sham | Baseline | 40 | 4 | 4 | -0.15 | -1.35 | 1.06 |
| Hearing Loss Model (Adult 2nd cohort) | Open field | sham | Baseline | 48 | 4 | 4 | -0.18 | -1.38 | 1.03 |
| Hearing Loss Model (Adult 2nd cohort) | Open field | sham | Baseline | 56 | 4 | 4 | -0.29 | -1.49 | 0.94 |
| Parkinsonism Model | Clinical Score | 6-OHDA | Sham | 1 | 10 | 7 | 1.98 | 1.02 | 2.95 |
| Parkinsonism Model | Clinical Score | 6-OHDA | Sham | 2 | 10 | 7 | 1.16 | 0.19 | 2.13 |
| Parkinsonism Model | Clinical Score | 6-OHDA | Sham | 3 | 10 | 7 | 0.93 | -0.04 | 1.89 |
| Parkinsonism Model | Clinical Score | 6-OHDA | Sham | 4 | 10 | 7 | 0.93 | -0.04 | 1.89 |
| Parkinsonism Model | Clinical Score | 6-OHDA | Baseline | 1 | 10 | 10 | 1.98 | 1.11 | 2.86 |
| Parkinsonism Model | Clinical Score | 6-OHDA | Baseline | 2 | 10 | 10 | 1.16 | 0.28 | 2.04 |
| Parkinsonism Model | Clinical Score | 6-OHDA | Baseline | 3 | 10 | 10 | 0.93 | 0.05 | 1.80 |
| Parkinsonism Model | Clinical Score | 6-OHDA | Baseline | 4 | 10 | 10 | 0.93 | 0.05 | 1.80 |
| Parkinsonism Model | Open field | 6-OHDA | Sham | -1 | 10 | 7 | -0.08 | -1.00 | 0.84 |
| Parkinsonism Model | Open field | 6-OHDA | Sham | 1 | 10 | 7 | -0.84 | -1.80 | 0.13 |
| Parkinsonism Model | Open field | 6-OHDA | Sham | 2 | 10 | 7 | -1.91 | -3.03 | -0.75 |
| Parkinsonism Model | Open field | 6-OHDA | Sham | 3 | 10 | 7 | -1.19 | -2.18 | -0.16 |
| Parkinsonism Model | Open field | 6-OHDA | Sham | 4 | 10 | 7 | -1.69 | -2.76 | -0.57 |
| Parkinsonism Model | Open field | 6-OHDA+electrode | Sham | -1 | 10 | 7 | -0.90 | -1.86 | 0.08 |
| Parkinsonism Model | Open field | 6-OHDA+electrode | Sham | 1 | 10 | 7 | -0.86 | -1.81 | 0.12 |
| Parkinsonism Model | Open field | 6-OHDA+electrode | Sham | 2 | 10 | 7 | -0.57 | -1.50 | 0.38 |
| Parkinsonism Model | Open field | 6-OHDA+electrode | Sham | 3 | 10 | 7 | -1.20 | -2.19 | -0.17 |
| Parkinsonism Model | Open field | 6-OHDA+electrode | Sham | 4 | 10 | 7 | -1.50 | -2.54 | -0.42 |
| Parkinsonism Model | Open field | 6-OHDA | Baseline | 1 | 10 | 10 | -1.39 | -2.33 | -0.42 |
| Parkinsonism Model | Open field | 6-OHDA | Baseline | 2 | 10 | 10 | -1.69 | -2.68 | -0.67 |
| Parkinsonism Model | Open field | 6-OHDA | Baseline | 3 | 10 | 10 | -1.40 | -2.34 | -0.43 |
| Parkinsonism Model | Open field | 6-OHDA | Baseline | 4 | 10 | 10 | -1.24 | -2.16 | -0.29 |
| Parkinsonism Model | Open field | Vehicle | Baseline | 1 | 7 | 7 | -0.55 | -1.54 | 0.46 |
| Parkinsonism Model | Open field | Vehicle | Baseline | 2 | 7 | 7 | 0.49 | -0.52 | 1.48 |
| Parkinsonism Model | Open field | Vehicle | Baseline | 3 | 7 | 7 | -0.19 | -1.17 | 0.79 |
| Parkinsonism Model | Open field | Vehicle | Baseline | 4 | 7 | 7 | 0.44 | -0.56 | 1.43 |
| Parkinsonism Model | Open field | 6-OHDA+electrode | Baseline | 1 | 10 | 10 | -0.50 | -1.35 | 0.36 |
| Parkinsonism Model | Open field | 6-OHDA+electrode | Baseline | 2 | 10 | 10 | -0.59 | -1.44 | 0.28 |
| Parkinsonism Model | Open field | 6-OHDA+electrode | Baseline | 3 | 10 | 10 | -0.67 | -1.53 | 0.21 |
| Parkinsonism Model | Open field | 6-OHDA+electrode | Baseline | 4 | 10 | 10 | -0.51 | -1.35 | 0.36 |
| Parkinsonism Model | Open field | Vehicle+electrode | Baseline | 1 | 7 | 7 | -1.28 | -2.37 | -0.16 |
| Parkinsonism Model | Open field | Vehicle+electrode | Baseline | 2 | 7 | 7 | -1.96 | -3.19 | -0.69 |
| Parkinsonism Model | Open field | Vehicle+electrode | Baseline | 3 | 7 | 7 | -1.23 | -2.31 | -0.12 |
| Parkinsonism Model | Open field | Vehicle+electrode | Baseline | 4 | 7 | 7 | -0.29 | -1.27 | 0.71 |
| Glioma Model | Open field | BT4Ca_rat_glioma_cells | Baseline | 1 | 8 | 8 | -0.26 | -1.18 | 0.68 |
| Glioma Model | Open field | BT4Ca_rat_glioma_cells | Baseline | 2 | 8 | 8 | -0.25 | -1.17 | 0.69 |
| Glioma Model | Open field | BT4Ca_rat_glioma_cells | Baseline | 3 | 8 | 8 | 0.16 | -0.77 | 1.09 |
| Glioma Model | Open field | BT4Ca_rat_glioma_cells | Baseline | 4 | 8 | 8 | 0.10 | -0.83 | 1.02 |
| Glioma Model | Open field | Tumor_resection | Baseline | 1 | 8 | 8 | 0.11 | -0.82 | 1.03 |
| Glioma Model | Open field | Tumor_resection | Baseline | 2 | 8 | 8 | 0.19 | -0.74 | 1.12 |
| Glioma Model | Open field | Tumor_resection | Baseline | 3 | 8 | 8 | 0.32 | -0.62 | 1.25 |
| Glioma Model | Open field | Tumor_resection | Baseline | 4 | 8 | 8 | 0.36 | -0.58 | 1.29 |
| Glioma Model (endpoint) | Clinical Score | Glioma-only | Baseline | -1 | 9 | 9 | 3.14 | 2.22 | 4.07 |
| Glioma Model (endpoint) | Clinical Score | Glioma-only | Baseline | -2 | 9 | 9 | 0.68 | -0.24 | 1.60 |
| Glioma Model (endpoint) | Clinical Score | Glioma-only | Baseline | -3 | 9 | 9 | 0.68 | -0.24 | 1.60 |
| Glioma Model (endpoint) | Clinical Score | Glioma-only | Baseline | -4 | 9 | 9 | 0.68 | -0.24 | 1.60 |
| Glioma Model (endpoint) | Clinical Score | Glioma-only | Baseline | -5 | 9 | 9 | 0.68 | -0.24 | 1.60 |
| Glioma Model (endpoint) | Clinical Score | Glioma-only | Baseline | -6 | 9 | 9 | 0.68 | -0.24 | 1.60 |
| Glioma Model (endpoint) | Clinical Score | Glioma-only | Baseline | -7 | 9 | 9 | 0.68 | -0.24 | 1.60 |
| Glioma Model (endpoint) | Clinical Score | Glioma-only | Baseline | 0 | 9 | 9 | 3.14 | 2.22 | 4.07 |
| Glioma Model (endpoint) | Open field | Glioma-only | Baseline | -1 | 9 | 9 | -1.07 | -2.01 | -0.10 |
| Glioma Model (endpoint) | Open field | Glioma-only | Baseline | -2 | 9 | 9 | -0.24 | -1.12 | 0.65 |
| Glioma Model (endpoint) | Open field | Glioma-only | Baseline | -3 | 9 | 9 | 0.01 | -0.87 | 0.89 |
| Glioma Model (endpoint) | Open field | Glioma-only | Baseline | -4 | 9 | 9 | -0.12 | -1.00 | 0.76 |
| Glioma Model (endpoint) | Open field | Glioma-only | Baseline | -5 | 9 | 9 | -0.91 | -1.83 | 0.04 |
| Glioma Model (endpoint) | Open field | Glioma-only | Baseline | -6 | 9 | 9 | -0.04 | -0.92 | 0.84 |
| Glioma Model (endpoint) | Open field | Glioma-only | Baseline | -7 | 9 | 9 | -0.09 | -0.96 | 0.80 |
| Glioma Model (endpoint) | Open field | Glioma-only | Baseline | 0 | 9 | 9 | -0.79 | -1.70 | 0.14 |
| Cerebellar Cognitive Affective Syndrome Model | Clinical Score | Lesion | Naïve | 1 | 22 | 18 | 2.71 | 2.09 | 3.33 |
| Cerebellar Cognitive Affective Syndrome Model | Clinical Score | Lesion | Naïve | 2 | 22 | 18 | 2.71 | 2.09 | 3.33 |
| Cerebellar Cognitive Affective Syndrome Model | Clinical Score | Lesion | Sham | 1 | 22 | 20 | 2.71 | 2.11 | 3.32 |
| Cerebellar Cognitive Affective Syndrome Model | Clinical Score | Lesion | Sham | 2 | 22 | 20 | 2.71 | 2.11 | 3.32 |
| Cerebellar Cognitive Affective Syndrome Model | Clinical Score | Lesion | Baseline | 1 | 22 | 22 | 2.71 | 2.12 | 3.30 |
| Cerebellar Cognitive Affective Syndrome Model | Clinical Score | Lesion | Baseline | 2 | 22 | 22 | 2.71 | 2.12 | 3.30 |
| Cerebellar Cognitive Affective Syndrome Model | Open field | Lesion | Naïve | -1 | 22 | 18 | -0.15 | -0.76 | 0.46 |
| Cerebellar Cognitive Affective Syndrome Model | Open field | Lesion | Naïve | 1 | 22 | 18 | -1.31 | -1.98 | -0.62 |
| Cerebellar Cognitive Affective Syndrome Model | Open field | Lesion | Naïve | 14 | 22 | 18 | -0.04 | -0.65 | 0.57 |
| Cerebellar Cognitive Affective Syndrome Model | Open field | Lesion | Naïve | 2 | 22 | 18 | -0.54 | -1.16 | 0.08 |
| Cerebellar Cognitive Affective Syndrome Model | Open field | Lesion | Naïve | 21 | 22 | 18 | 0.44 | -0.18 | 1.05 |
| Cerebellar Cognitive Affective Syndrome Model | Open field | Lesion | Naïve | 28 | 22 | 18 | -0.14 | -0.75 | 0.47 |
| Cerebellar Cognitive Affective Syndrome Model | Open field | Lesion | Naïve | 35 | 22 | 18 | 0.10 | -0.51 | 0.71 |
| Cerebellar Cognitive Affective Syndrome Model | Open field | Lesion | Naïve | 42 | 22 | 18 | 0.17 | -0.45 | 0.78 |
| Cerebellar Cognitive Affective Syndrome Model | Open field | Lesion | Naïve | 7 | 22 | 18 | -0.37 | -0.98 | 0.25 |
| Cerebellar Cognitive Affective Syndrome Model | Open field | Lesion | Sham | -1 | 22 | 20 | -0.08 | -0.67 | 0.52 |
| Cerebellar Cognitive Affective Syndrome Model | Open field | Lesion | Sham | 1 | 22 | 20 | -1.11 | -1.75 | -0.46 |
| Cerebellar Cognitive Affective Syndrome Model | Open field | Lesion | Sham | 14 | 22 | 20 | 0.00 | -0.60 | 0.59 |
| Cerebellar Cognitive Affective Syndrome Model | Open field | Lesion | Sham | 2 | 22 | 20 | -0.40 | -1.00 | 0.20 |
| Cerebellar Cognitive Affective Syndrome Model | Open field | Lesion | Sham | 21 | 22 | 20 | 0.14 | -0.46 | 0.73 |
| Cerebellar Cognitive Affective Syndrome Model | Open field | Lesion | Sham | 28 | 22 | 20 | -0.50 | -1.10 | 0.11 |
| Cerebellar Cognitive Affective Syndrome Model | Open field | Lesion | Sham | 35 | 22 | 20 | -0.05 | -0.64 | 0.55 |
| Cerebellar Cognitive Affective Syndrome Model | Open field | Lesion | Sham | 42 | 22 | 20 | 0.06 | -0.53 | 0.66 |
| Cerebellar Cognitive Affective Syndrome Model | Open field | Lesion | Sham | 7 | 22 | 20 | -0.43 | -1.03 | 0.18 |
| Cerebellar Cognitive Affective Syndrome Model | Open field | Control | Baseline | 1 | 18 | 18 | 0.38 | -0.26 | 1.03 |
| Cerebellar Cognitive Affective Syndrome Model | Open field | Control | Baseline | 14 | 18 | 18 | 0.39 | -0.25 | 1.04 |
| Cerebellar Cognitive Affective Syndrome Model | Open field | Control | Baseline | 2 | 18 | 18 | 0.13 | -0.51 | 0.77 |
| Cerebellar Cognitive Affective Syndrome Model | Open field | Control | Baseline | 21 | 18 | 18 | 0.16 | -0.48 | 0.80 |
| Cerebellar Cognitive Affective Syndrome Model | Open field | Control | Baseline | 28 | 18 | 18 | 0.24 | -0.40 | 0.88 |
| Cerebellar Cognitive Affective Syndrome Model | Open field | Control | Baseline | 35 | 18 | 18 | 0.08 | -0.56 | 0.72 |
| Cerebellar Cognitive Affective Syndrome Model | Open field | Control | Baseline | 42 | 18 | 18 | 0.01 | -0.62 | 0.65 |
| Cerebellar Cognitive Affective Syndrome Model | Open field | Control | Baseline | 7 | 18 | 18 | 0.35 | -0.30 | 0.99 |
| Cerebellar Cognitive Affective Syndrome Model | Open field | Lesion | Baseline | 1 | 22 | 22 | -0.78 | -1.38 | -0.17 |
| Cerebellar Cognitive Affective Syndrome Model | Open field | Lesion | Baseline | 14 | 22 | 22 | 0.46 | -0.13 | 1.04 |
| Cerebellar Cognitive Affective Syndrome Model | Open field | Lesion | Baseline | 2 | 22 | 22 | -0.35 | -0.93 | 0.24 |
| Cerebellar Cognitive Affective Syndrome Model** | Open field | Lesion | Baseline | 21 | 22 | 22 | 0.74 | 0.14 | 1.34 |
| Cerebellar Cognitive Affective Syndrome Model | Open field | Lesion | Baseline | 28 | 22 | 22 | 0.34 | -0.25 | 0.92 |
| Cerebellar Cognitive Affective Syndrome Model | Open field | Lesion | Baseline | 35 | 22 | 22 | 0.37 | -0.22 | 0.95 |
| Cerebellar Cognitive Affective Syndrome Model** | Open field | Lesion | Baseline | 42 | 22 | 22 | 0.34 | -0.25 | 0.92 |
| Cerebellar Cognitive Affective Syndrome Model | Open field | Lesion | Baseline | 7 | 22 | 22 | 0.06 | -0.52 | 0.64 |
| Cerebellar Cognitive Affective Syndrome Model | Open field | Sham | Baseline | 1 | 20 | 20 | 0.28 | -0.33 | 0.89 |
| Cerebellar Cognitive Affective Syndrome Model | Open field | Sham | Baseline | 14 | 20 | 20 | 0.49 | -0.13 | 1.11 |
| Cerebellar Cognitive Affective Syndrome Model | Open field | Sham | Baseline | 2 | 20 | 20 | 0.01 | -0.60 | 0.61 |
| Cerebellar Cognitive Affective Syndrome Model | Open field | Sham | Baseline | 21 | 20 | 20 | 0.59 | -0.03 | 1.21 |
| Cerebellar Cognitive Affective Syndrome Model | Open field | Sham | Baseline | 28 | 20 | 20 | 0.79 | 0.16 | 1.42 |
| Cerebellar Cognitive Affective Syndrome Model | Open field | Sham | Baseline | 35 | 20 | 20 | 0.39 | -0.23 | 1.00 |
| Cerebellar Cognitive Affective Syndrome Model | Open field | Sham | Baseline | 42 | 20 | 20 | 0.27 | -0.34 | 0.88 |
| Cerebellar Cognitive Affective Syndrome Model | Open field | Sham | Baseline | 7 | 20 | 20 | 0.63 | 0.00 | 1.25 |

*, Cohen’s H for Clinical Score and Neuroscore; Hedge’s G for Open field, and VWR.

**, Please note that the data selected for analysis were from day 42. The increase in activity on day 21 may be related to developmental-stage changes rather than the model.

Experimental days refer to the days of and following the main intervention in the model (e.g., start of the kindling procedure for kindling experiments, SE induction for SE-based models, etc.).

For Clinical Score, days/comparisons without changes were not included in the table.

hip, Hippocampus; bla, Basolateral amygdala; tm, transmitter; SE, Status epilepticus; IHK, intrahippocampal kainate; DSS, Dextran Sodium Sulfate; pilo, pilocarpine; PET, Positron Emission Tomography; tele, telemetry; teth, tethered; 6OHDA, 6-hydroxydopamine.

Table S16. Effect sizes and confidence intervals in the “non-genetic disease models” category included in the telemetry sub-analysis.

| **Model ID** | **Readout parameter** | **Intervention group** | **Reference data** | **Experimental day(s)** | **Sample size** | | **Effect size*** | **Confidence interval** | |
| --- | --- | --- | --- | --- | --- | --- | --- | --- | --- |
|  |  |  |  |  | **Exp/ Int** | **Reference** |  | **Lower** | **Higher** |
| Epilepsy Model – Kindling (tele) | Telemetry (light-on) | Kindling | Sham | -3 and -2 (baseline) | 6 | 6 | -0.85 | -2.20 | 0.49 |
| Epilepsy Model – Kindling (tele) | Telemetry (light-on) | Kindling | Sham | 2 | 6 | 6 | -0.42 | -1.72 | 0.88 |
| Epilepsy Model – Kindling (tele) | Telemetry (light-on) | Kindling | Sham | 3 | 5 | 6 | 0.27 | -1.10 | 1.65 |
| Epilepsy Model – Kindling (tele) | Telemetry (light-on) | Kindling | Sham | 15 | 6 | 6 | 1.78 | 0.26 | 3.30 |
| Epilepsy Model – Kindling (tele) | Telemetry (light-on) | Kindling | Sham | 16 | 6 | 6 | 1.25 | -0.16 | 2.66 |
| Epilepsy Model – Kindling (tele) | Telemetry (light-on) | Kindling | Sham (tm) | -3 and -2 (baseline) | 6 | 6 | 1.03 | -0.34 | 2.39 |
| Epilepsy Model – Kindling (tele) | Telemetry (light-on) | Kindling | Sham (tm) | 2 | 6 | 6 | 0.80 | -0.53 | 2.14 |
| Epilepsy Model – Kindling (tele) | Telemetry (light-on) | Kindling | Sham (tm) | 3 | 5 | 6 | 0.77 | -0.65 | 2.18 |
| Epilepsy Model – Kindling (tele) | Telemetry (light-on) | Kindling | Sham (tm) | 15 | 6 | 6 | 2.14 | 0.53 | 3.75 |
| Epilepsy Model – Kindling (tele) | Telemetry (light-on) | Kindling | Sham (tm) | 16 | 6 | 6 | 1.44 | 0.00 | 2.88 |
| Epilepsy Model – Kindling (tele) | Telemetry (light-off) | Kindling | Sham | -3 and -2 (baseline) | 6 | 6 | -1.03 | -2.40 | 0.34 |
| Epilepsy Model – Kindling (tele) | Telemetry (light-off) | Kindling | Sham | 1 | 6 | 6 | -0.46 | -1.76 | 0.85 |
| Epilepsy Model – Kindling (tele) | Telemetry (light-off) | Kindling | Sham | 2 | 5 | 6 | -0.99 | -2.44 | 0.46 |
| Epilepsy Model – Kindling (tele) | Telemetry (light-off) | Kindling | Sham | 3 | 3 | 6 | / | / | / |
| Epilepsy Model – Kindling (tele) | Telemetry (light-off) | Kindling | Sham | 14 | 6 | 6 | 0.53 | -0.78 | 1.84 |
| Epilepsy Model – Kindling (tele) | Telemetry (light-off) | Kindling | Sham | 15 | 6 | 6 | 0.17 | -1.12 | 1.46 |
| Epilepsy Model – Kindling (tele) | Telemetry (light-off) | Kindling | Sham | 16 | 6 | 6 | 0.73 | -0.60 | 2.05 |
| Epilepsy Model – Kindling (tele) | Telemetry (light-off) | Kindling | Sham (tm) | -3 and -2 (baseline) | 6 | 6 | 1.23 | -0.17 | 2.63 |
| Epilepsy Model – Kindling (tele) | Telemetry (light-off) | Kindling | Sham (tm) | 1 | 6 | 6 | 0.63 | -0.69 | 1.95 |
| Epilepsy Model – Kindling (tele) | Telemetry (light-off) | Kindling | Sham (tm) | 2 | 5 | 6 | 0.31 | -1.07 | 1.68 |
| Epilepsy Model – Kindling (tele) | Telemetry (light-off) | Kindling | Sham (tm) | 3 | 3 | 6 | / | / | / |
| Epilepsy Model – Kindling (tele) | Telemetry (light-off) | Kindling | Sham (tm) | 14 | 6 | 6 | 0.40 | -0.90 | 1.70 |
| Epilepsy Model – Kindling (tele) | Telemetry (light-off) | Kindling | Sham (tm) | 15 | 6 | 6 | 0.85 | -0.49 | 2.19 |
| Epilepsy Model – Kindling (tele) | Telemetry (light-off) | Kindling | Sham (tm) | 16 | 6 | 6 | 0.60 | -0.71 | 1.92 |
| Epilepsy Model – Kindling (tele) | Telemetry (light-off) | Kindling | Baseline  (-3 and -2) | 1 | 6 | 6 | -0.07 | -1.35 | 1.22 |
| Epilepsy Model – Kindling (tele) | Telemetry (light-off) | Kindling | Baseline  (-3 and -2) | 2 | 5 | 6 | -0.77 | -2.19 | 0.65 |
| Epilepsy Model – Kindling (tele) | Telemetry (light-off) | Kindling | Baseline  (-3 and -2) | 3 | 3 | 6 | / | / | / |
| Epilepsy Model – Kindling (tele) | Telemetry (light-off) | Kindling | Baseline  (-3 and -2) | 14 | 6 | 6 | 0.19 | -1.10 | 1.47 |
| Epilepsy Model – Kindling (tele) | Telemetry (light-off) | Kindling | Baseline  (-3 and -2) | 15 | 6 | 6 | 0.32 | -0.97 | 1.62 |
| Epilepsy Model – Kindling (tele) | Telemetry (light-off) | Kindling | Baseline  (-3 and -2) | 16 | 6 | 6 | -0.01 | -1.30 | 1.27 |
| Epilepsy Model – Kindling (tele) | Telemetry (light-off) | Sham | Baseline  (-3 and -2) | 1 | 6 | 6 | -0.46 | -1.76 | 0.85 |
| Epilepsy Model – Kindling (tele) | Telemetry (light-off) | Sham | Baseline  (-3 and -2) | 2 | 6 | 6 | -0.55 | -1.86 | 0.76 |
| Epilepsy Model – Kindling (tele) | Telemetry (light-off) | Sham | Baseline  (-3 and -2) | 3 | 6 | 6 | -1.07 | -2.45 | 0.31 |
| Epilepsy Model – Kindling (tele) | Telemetry (light-off) | Sham | Baseline  (-3 and -2) | 14 | 6 | 6 | -1.52 | -2.98 | -0.06 |
| Epilepsy Model – Kindling (tele) | Telemetry (light-off) | Sham | Baseline  (-3 and -2) | 15 | 6 | 6 | -0.56 | -1.88 | 0.75 |
| Epilepsy Model – Kindling (tele) | Telemetry (light-off) | Sham | Baseline  (-3 and -2) | 16 | 6 | 6 | -1.97 | -3.54 | -0.40 |
| Epilepsy Model – Kindling (tele) | Telemetry (light-off) | Sham (tm) | Baseline  (-3 and -2) | 1 | 6 | 6 | 0.36 | -0.93 | 1.66 |
| Epilepsy Model – Kindling (tele) | Telemetry (light-off) | Sham (tm) | Baseline  (-3 and -2) | 2 | 6 | 6 | 0.26 | -1.03 | 1.56 |
| Epilepsy Model – Kindling (tele) | Telemetry (light-off) | Sham (tm) | Baseline  (-3 and -2) | 3 | 6 | 6 | 0.41 | -0.89 | 1.71 |
| Epilepsy Model – Kindling (tele) | Telemetry (light-off) | Sham (tm) | Baseline  (-3 and -2) | 14 | 6 | 6 | 0.61 | -0.70 | 1.93 |
| Epilepsy Model – Kindling (tele) | Telemetry (light-off) | Sham (tm) | Baseline  (-3 and -2) | 15 | 6 | 6 | 0.44 | -0.86 | 1.74 |
| Epilepsy Model – Kindling (tele) | Telemetry (light-off) | Sham (tm) | Baseline  (-3 and -2) | 16 | 6 | 6 | 0.27 | -1.03 | 1.56 |
| Epilepsy Model – Post SE (IHK) | Telemetry (light-on) | IHK | Sham | 14-15  (chronic) | 12 | 8 | -0.03 | -0.99 | 0.93 |
| Epilepsy Model – Post SE (IHK) | Telemetry (light-on) | IHK | Sham | 28-29  (chronic) | 12 | 8 | 0.40 | -0.57 | 1.37 |
| Epilepsy Model – Post SE (IHK) | Telemetry (light-on) | IHK | Sham (tm) | 14-15  (chronic) | 12 | 8 | -0.37 | -1.34 | 0.60 |
| Epilepsy Model – Post SE (IHK) | Telemetry (light-on) | IHK | Sham (tm) | 28-29  (chronic) | 12 | 8 | 0.01 | -0.95 | 0.97 |
| Epilepsy Model – Post SE (IHK) | Telemetry (light-off) | IHK | Sham | 14-15  (chronic) | 12 | 8 | 0.35 | -0.61 | 1.32 |
| Epilepsy Model – Post SE (IHK) | Telemetry (light-off) | IHK | Sham | 28-29  (chronic) | 12 | 8 | 0.50 | -0.47 | 1.47 |
| Epilepsy Model – Post SE (IHK) | Telemetry (light-off) | IHK | Sham (tm) | 14-15  (chronic) | 12 | 8 | 0.65 | -0.33 | 1.63 |
| Epilepsy Model – Post SE (IHK) | Telemetry (light-off) | IHK | Sham (tm) | 28-29  (chronic) | 12 | 8 | 0.48 | -0.50 | 1.45 |
| Epilepsy Model – Post SE (pilo) | Telemetry (light-on) | SE-Pilocarpine | Sham | -3 and -2 (baseline) | 11 | 6 | 0.66 | -0.45 | 1.77 |
| Epilepsy Model – Post SE (pilo) | Telemetry (light-on) | SE-Pilocarpine | Sham | 5-6 (early) | 11 | 6 | 1.34 | 0.15 | 2.53 |
| Epilepsy Model – Post SE (pilo) | Telemetry (light-on) | SE-Pilocarpine | Sham | 26-27 (latency) | 11 | 6 | -0.67 | -1.78 | 0.44 |
| Epilepsy Model – Post SE (pilo) | Telemetry (light-on) | SE-Pilocarpine | Sham | 63-64 (chronic) | 11 | 6 | -0.01 | -1.10 | 1.07 |
| Epilepsy Model – Post SE (pilo) | Telemetry (light-off) | SE-Pilocarpine | Sham | -3 and -2 (baseline) | 11 | 6 | 0.10 | -0.98 | 1.19 |
| Epilepsy Model – Post SE (pilo) | Telemetry (light-off) | SE-Pilocarpine | Sham | 5-6 (early) | 11 | 6 | 2.66 | 1.21 | 4.12 |
| Epilepsy Model – Post SE (pilo) | Telemetry (light-off) | SE-Pilocarpine | Sham | 26-27 (latency) | 11 | 6 | 1.21 | 0.04 | 2.37 |
| Epilepsy Model – Post SE (pilo) | Telemetry (light-off) | SE-Pilocarpine | Sham | 63-64 (chronic) | 11 | 6 | 1.15 | -0.01 | 2.31 |
| Epilepsy Model – Post SE (pilo) | Telemetry (light-off) | Sham | Baseline  (-3 and -2) | 5-6 (early) | 6 | 6 | -0.60 | -1.92 | 0.71 |
| Epilepsy Model – Post SE (pilo) | Telemetry (light-off) | Sham | Baseline  (-3 and -2) | 26-27 (latency) | 6 | 6 | 0.17 | -1.12 | 1.46 |
| Epilepsy Model – Post SE (pilo) | Telemetry (light-off) | Sham | Baseline  (-3 and -2) | 63-64 (chronic) | 6 | 6 | -1.08 | -2.45 | 0.30 |
| Epilepsy Model – Post SE (pilo) | Telemetry (light-off) | SE-Pilocarpine | Baseline  (-3 and -2) | 5-6 (early) | 11 | 11 | 2.01 | 0.92 | 3.11 |
| Epilepsy Model – Post SE (pilo) | Telemetry (light-off) | SE-Pilocarpine | Baseline  (-3 and -2) | 26-27 (latency) | 11 | 11 | 1.42 | 0.42 | 2.41 |
| Epilepsy Model – Post SE (pilo) | Telemetry (light-off) | SE-Pilocarpine | Baseline  (-3 and -2) | 63-64 (chronic) | 11 | 11 | 0.35 | -0.55 | 1.25 |
| Epilepsy Model – Post SE (electrical) | Telemetry (light-on) | SE-Electrical | Sham | -2 and -1 (baseline) | 7 | 6 | -0.45 | -1.69 | 0.79 |
| Epilepsy Model – Post SE (electrical) | Telemetry (light-on) | SE-Electrical | Sham | 5-6 (early) | 7 | 6 | 1.53 | 0.14 | 2.92 |
| Epilepsy Model – Post SE (electrical) | Telemetry (light-on) | SE-Electrical | Sham | 26-27 (latency) | 7 | 6 | -0.82 | -2.09 | 0.46 |
| Epilepsy Model – Post SE (electrical) | Telemetry (light-on) | SE-Electrical | Sham | 61-62 (chronic) | 7 | 6 | 0.44 | -0.80 | 1.67 |
| Epilepsy Model – Post SE (electrical) | Telemetry (light-off) | SE-Electrical | Sham | -2 and -1 (baseline) | 7 | 6 | -0.64 | -1.90 | 0.61 |
| Epilepsy Model – Post SE (electrical) | Telemetry (light-off) | SE-Electrical | Sham | 5-6 (early) | 7 | 6 | -0.09 | -1.31 | 1.14 |
| Epilepsy Model – Post SE (electrical) | Telemetry (light-off) | SE-Electrical | Sham | 26-27 (latency) | 7 | 6 | 0.52 | -0.72 | 1.77 |
| Epilepsy Model – Post SE (electrical) | Telemetry (light-off) | SE-Electrical | Sham | 61-62 (chronic) | 7 | 6 | 0.83 | -0.45 | 2.10 |
| Epilepsy Model – Post SE (electrical) | Telemetry (light-off) | Sham | Baseline  (-2 and -1) | 5-6 (early) | 6 | 6 | 0.52 | -0.79 | 1.82 |
| Epilepsy Model – Post SE (electrical) | Telemetry (light-off) | Sham | Baseline  (-2 and -1) | 26-27 (latency) | 6 | 6 | 0.83 | -0.51 | 2.18 |
| Epilepsy Model – Post SE (electrical) | Telemetry (light-off) | Sham | Baseline  (-2 and -1) | 61-62 (chronic) | 6 | 6 | -0.76 | -2.09 | 0.57 |
| Epilepsy Model – Post SE (electrical) | Telemetry (light-off) | SE-Electrical | Baseline  (-2 and -1) | 5-6 (early) | 7 | 7 | 0.65 | -0.54 | 1.85 |
| Epilepsy Model – Post SE (electrical) | Telemetry (light-off) | SE-Electrical | Baseline  (-2 and -1) | 26-27 (latency) | 7 | 7 | 1.33 | 0.05 | 2.62 |
| Epilepsy Model – Post SE (electrical) | Telemetry (light-off) | SE-Electrical | Baseline  (-2 and -1) | 61-62 (chronic) | 7 | 7 | 0.81 | -0.40 | 2.02 |
| Glioma Model (endpoint) | Clinical Score | Glioma | Baseline  (~ day -17) | -2 | 7 | 7 | 1.13 | 0.08 | 2.18 |
| Glioma Model (endpoint) | Clinical Score | Glioma | Baseline  (~ day -17) | -1 | 7 | 7 | 1.71 | 0.67 | 2.76 |
| Glioma Model (endpoint) | Clinical Score | Glioma | Baseline  (~ day -17) | 0 (endpoint) | 7 | 7 | 3.14 | 2.09 | 4.19 |
| Glioma Model (endpoint) | Telemetry (light-off) | Glioma | Baseline  (~ day -17) | -8 | 7 | 7 | -0.07 | -1.23 | 1.10 |
| Glioma Model (endpoint) | Telemetry (light-off) | Glioma | Baseline  (~ day -17) | -7 | 7 | 7 | 0.55 | -0.64 | 1.73 |
| Glioma Model (endpoint) | Telemetry (light-off) | Glioma | Baseline  (~ day -17) | -6 | 7 | 7 | -0.17 | -1.33 | 1.00 |
| Glioma Model (endpoint) | Telemetry (light-off) | Glioma | Baseline  (~ day -17) | -5 | 7 | 7 | -0.41 | -1.59 | 0.77 |
| Glioma Model (endpoint) | Telemetry (light-off) | Glioma | Baseline  (~ day -17) | -4 | 7 | 7 | -0.43 | -1.61 | 0.75 |
| Glioma Model (endpoint) | Telemetry (light-off) | Glioma | Baseline  (~ day -17) | -3 | 7 | 7 | -1.04 | -2.28 | 0.20 |
| Glioma Model (endpoint) | Telemetry (light-off) | Glioma | Baseline  (~ day -17) | -2 | 7 | 7 | -1.40 | -2.70 | -0.10 |
| Glioma Model (endpoint) | Telemetry (light-off) | Glioma | Baseline  (~ day -17) | -1 | 7 | 7 | -1.22 | -2.49 | 0.05 |
| Glioma Model (endpoint) | Telemetry (light-off) | Glioma | Baseline  (~ day -17) | 0 (endpoint) | 7 | 7 | -1.53 | -2.85 | -0.21 |
| Pancreatic Cancer Model | Telemetry (light-off) | Pancreatic cancer | Baseline  (day -1) | 4 | 7 | 10 | 0.28 | -0.78 | 1.33 |
| Pancreatic Cancer Model | Telemetry (light-off) | Pancreatic cancer | Baseline  (day -1) | 18 | 7 | 10 | 0.77 | -0.32 | 1.85 |
| Pancreatic Cancer Model | Telemetry (light-off) | Pancreatic cancer | Baseline  (day -1) | 34 | 7 | 10 | 0.55 | -0.52 | 1.62 |

*, Cohen’s H for Clinical Score and Hedge’s G for Telemetry.

Experimental days refer to the days of and following the main intervention in the model (e.g., start of the kindling procedure for kindling experiments, SE induction for SE-based models, etc.).

For Clinical Score, days/comparisons without changes were not included in the table.

Baseline data includes data from the end of the laboratory-specific recovery phase (note that telemetric data can only be collected following surgery).

Exp/Int, Experimental/Intervention group; Sham (tm), transmitter only sham; IHK, intrahippocampal kainate; /, no calculations for this time point due to over 20 % missing data; SE, Status epilepticus.

Table S17. AUC (95% confidence interval) for the analyzed studies.

| **Included model IDs** | **Gold standard** | **Readout parameter** | **Subgrouping variable** | **Reduced activity**  **AUC (95% CI)** | **Increased activity**  **AUC (95% CI)** |
| --- | --- | --- | --- | --- | --- |
| Partial Hepatectomy | Telemetry | Clinical Score | Experimental day = 1 | 0.48 (0.38, 0.57) |  |
|  |  | Open field | Experimental day = -2 | 0.91 (0.76, 1.00) |  |
|  |  |  | Experimental day = 1 | 0.56 (0.34, 0.78) |  |
|  |  |  | Experimental day = 3 | 0.50 (0.29, 0.71) |  |
| Intracerebral Electrode Implantation | HC-based video monitoring (dark phase) | Clinical Score | Sex = Female | 0.50 (0.47, 0.52) |  |
|  |  |  | Sex = Male | 0.51 (0.46, 0.56) |  |
|  |  | Neuroscore | Sex = Female | 0.52 (0.50, 0.55) | 0.51 (0.49, 0.54) |
|  |  |  | Sex = Male | 0.45 (0.40, 0.51) | 0.53 (0.51, 0.57) |
|  |  | VWR (light phase) | Sex = Female | 0.59 (0.50, 0.69) | 0.56 (0.47, 0.66) |
|  |  |  | Sex = Male | 0.68 (0.55, 0.81) | 0.56 (0.46, 0.66) |
|  |  | VWR (dark phase) | Sex = Female | 0.84 (0.77, 0.90) | 0.83 (0.76, 0.90) |
|  |  |  | Sex = Male | 0.91 (0.85, 0.97) | 0.88 (0.82, 0.94) |
|  |  | HC-based video monitoring (light phase) | Sex = Female | 0.56 (0.40, 0.66) | 0.59 (0.50, 0.69) |
|  |  |  | Sex = Male | 0.66 (0.56, 0.77) | 0.64 (0.54, 0.74) |
| Epilepsy Model – Post SE (pilo + electrical) | Telemetry (dark phase) | Neuroscore |  |  | 0.51 (0.34, 0.69) |
|  |  | Open field |  | 0.47 (0.23, 0.71) | 0.63 (0.32, 0.94) |
|  |  | Telemetry (light phase) |  | 0.91 (0.81, 1.00) | 0.83 (0.66, 1.00) |
| Dravet Syndrome Model + *Gria1* KO Model | HC-based video monitoring | Neuroscore | Developmental stage = Prepubescence |  | 0.50 (0.43, 0.57) |
|  |  |  | Developmental stage = Sexual maturity |  | 0.53 (0.50, 0.56) |
|  |  | Open field | Developmental stage = Prepubescence | 0.60 (0.45, 0.74) | 0.59 (0.44, 0.74) |
|  |  |  | Developmental stage = Sexual maturity | 0.51 (0.37, 0.65) | 0.71 (0.60, 0.83) |

AUC, Area under the curve; CI, Confidence Interval; HC, Home cage; VWR, Voluntary wheel running; SE, Status epilepticus; pilo, pilocarpine.

Table S18. Correlation coefficients and statistical significance for the correlation analysis.

| **Included Model ID** | **Subgrouping variable** | **Parameter 1** | **Parameter 2** | **Correlation coefficient** | **p-value** |
| --- | --- | --- | --- | --- | --- |
| Partial Hepatectomy | Experimental day = 1 | Clinical Score | Open field | -0.09 | 0.6131 |
|  |  |  | Telemetry | 0.14 | 0.4339 |
|  |  | Open field | Telemetry | 0.08 | 0.6433 |
|  | Experimental day = 3 | Clinical Score | Open field | 0.07 | 0.6994 |
|  |  |  | Telemetry | -0.25 | 0.1566 |
|  |  | Open field | Telemetry | 0.16 | 0.3634 |
| Intracerebral Electrode Implantation | Sex = Female | Clinical Score | Neuroscore | -0.50 | 0.0000 |
|  |  |  | VWR (light phase) | -0.08 | 0.3505 |
|  |  |  | VWR (dark phase) | -0.09 | 0.2737 |
|  |  |  | HC-based video monitoring (light phase) | -0.04 | 0.6510 |
|  |  |  | HC-based video monitoring (dark phase) | -0.10 | 0.2442 |
|  |  | Neuroscore | VWR (light phase) | 0.08 | 0.3640 |
|  |  |  | VWR (dark phase) | 0.02 | 0.8239 |
|  |  |  | HC-based video monitoring (light phase) | 0.14 | 0.0857 |
|  |  |  | HC-based video monitoring (dark phase) | -0.03 | 0.7570 |
|  |  | VWR (light phase) | VWR (dark phase) | 0.29 | 0.0004 |
|  |  |  | HC-based video monitoring (light phase) | 0.49 | 0.0000 |
|  |  |  | HC-based video monitoring (dark phase) | 0.20 | 0.0137 |
|  |  | VWR (dark phase) | HC-based video monitoring (light phase) | 0.10 | 0.2374 |
|  |  |  | HC-based video monitoring (dark phase) | 0.73 | 0.0000 |
|  |  | HC-based video monitoring (light phase) | HC-based video monitoring (dark phase) | 0.17 | 0.0409 |
|  | Sex = Male | Clinical Score | Neuroscore | -0.46 | 0.0000 |
|  |  |  | VWR (light phase) | -0.01 | 0.8948 |
|  |  |  | VWR (dark phase) | -0.10 | 0.2698 |
|  |  |  | HC-based video monitoring (light phase) | -0.01 | 0.9408 |
|  |  |  | HC-based video monitoring (dark phase) | -0.06 | 0.4722 |
|  |  | Neuroscore | VWR (light phase) | 0.19 | 0.0265 |
|  |  |  | VWR (dark phase) | 0.13 | 0.1492 |
|  |  |  | HC-based video monitoring (light phase) | 0.17 | 0.0524 |
|  |  |  | HC-based video monitoring (dark phase) | 0.19 | 0.0278 |
|  |  | VWR (light phase) | VWR (dark phase) | 0.30 | 0.0005 |
|  |  |  | HC-based video monitoring (light phase) | 0.77 | 0.0000 |
|  |  |  | HC-based video monitoring (dark phase) | 0.21 | 0.0172 |
|  |  | VWR (dark phase) | HC-based video monitoring (light phase) | 0.20 | 0.0191 |
|  |  |  | HC-based video monitoring (dark phase) | 0.77 | 0.0000 |
|  |  | HC-based video monitoring (light phase) | HC-based video monitoring (dark phase) | 0.32 | 0.0001 |
| Pilocarpine Epilepsy Model + Electrical Epilepsy Model |  | Neuroscore | Open field | -0.44 | 0.0200 |
|  |  |  | Telemetry (light phase) | -0.03 | 0.8610 |
|  |  |  | Telemetry (dark phase) | -0.11 | 0.5857 |
|  |  | Open field | Telemetry (light phase) | -0.39 | 0.0419 |
|  |  |  | Telemetry (dark phase) | -0.05 | 0.7927 |
|  |  | Telemetry (light phase) | Telemetry (dark phase) | 0.67 | 0.0000 |
| Dravet Syndrome Model + *Gria1* Model | Developmental stage = Prepubescence | Neuroscore | Open field | 0.39 | 0.0004 |
|  |  |  | HC-based video monitoring | -0.05 | 0.6368 |
|  |  | Open field | HC-based video monitoring | 0.26 | 0.0241 |
|  | Developmental stage = Sexual maturity | Neuroscore | Open field | 0.19 | 0.1030 |
|  |  |  | HC-based video monitoring | -0.26 | 0.0232 |
|  |  | Open field | HC-based video monitoring | 0.13 | 0.2561 |

VWR, Voluntary wheel running; HC, Home cage.

**Supplementary References**

1	Wickham, H. *et al.* Welcome to the Tidyverse. *Journal of Open Source Software* **4**, 1686, doi:10.21105/joss.01686 (2019).

2	glue: Interpreted String Literals v. 1.8.0 (CRAN, CRAN, 2024).

3	Ben-Shachar, M. S., Lüdecke, D. & Makowski, D. effectsize: Estimation of Effect Size Indices and Standardized Parameters. *Journal of Open Source Software* **5**, 2815, doi:10.21105/joss.02815 (2020).

4	Balduzzi, S., Rucker, G. & Schwarzer, G. How to perform a meta-analysis with R: a practical tutorial. *Evid Based Ment Health* **22**, 153-160, doi:10.1136/ebmental-2019-300117 (2019).

5	Robin, X. *et al.* pROC: an open-source package for R and S+ to analyze and compare ROC curves. *BMC Bioinformatics* **12**, 77, doi:10.1186/1471-2105-12-77 (2011).

6	patchwork: The Composer of Plots v. 1.3.2 (2025).

7	Hmisc: Harrell Miscellaneous v. 5.2-4 (2025).

8	R package 'corrplot': Visualization of a Correlation Matrix v. 0.95 (2024).

9	rstatix: Pipe-Friendly Framework for Basic Statistical Tests (2025).

10	Häger, C. *et al.* Running in the wheel: Defining individual severity levels in mice. *PLoS Biol* **16**, e2006159, doi:10.1371/journal.pbio.2006159 (2018).

11	Talbot, S. R. *et al.* RELSA-A multidimensional procedure for the comparative assessment of well-being and the quantitative determination of severity in experimental procedures. *Front Vet Sci* **9**, 937711, doi:10.3389/fvets.2022.937711 (2022).

12	Zieglowski, L. *et al.* Assessing the severity of laparotomy and partial hepatectomy in male rats-A multimodal approach. *PLoS One* **16**, e0255175, doi:10.1371/journal.pone.0255175 (2021).

13	Zieglowski, L. *et al.* Severity assessment using three common behavioral or locomotor tests after laparotomy in rats: a pilot study. *Lab Anim* **54**, 525-535, doi:10.1177/0023677220911680 (2020).

14	Kümmecke, A. M., Zieglowski, L., Ernst, L., Palme, R. & Tolba, R. H. Does Sex Matter in Liver Surgery? Comparison of Severity Assessments between Female and Male Rats after Partial Hepatectomy: A Pilot Study. *Eur Surg Res* **64**, 65-76, doi:10.1159/000527334 (2023).

15	Mallien, A. S. *et al.* Dopamine Transporter Knockout Rats Show Impaired Wellbeing in a Multimodal Severity Assessment Approach. *Front Behav Neurosci* **16**, 924603, doi:10.3389/fnbeh.2022.924603 (2022).

16	Mallien, A. S. *et al.* Systematic analysis of severity in a widely used cognitive depression model for mice. *Lab Anim* **54**, 40-49, doi:10.1177/0023677219874831 (2020).

17	Becker, L. *et al.* Evidence-based severity assessment of the forced swim test in the rat. *PLoS One* **18**, e0292816, doi:10.1371/journal.pone.0292816 (2023).

18	Bach-Hagemann, A. *et al.* Severity Assessment in Rats Undergoing Subarachnoid Hemorrhage Induction by Endovascular Perforation or Corresponding Sham Surgery. *Eur Surg Res* **64**, 120-138, doi:10.1159/000524432 (2023).

19	Boldt, L. *et al.* Toward evidence-based severity assessment in mouse models with repeated seizures: I. Electrical kindling. *Epilepsy Behav* **115**, 107689, doi:10.1016/j.yebeh.2020.107689 (2021).

20	Möller, C. *et al.* Toward evidence-based severity assessment in rat models with repeated seizures: I. Electrical kindling. *Epilepsia* **59**, 765-777, doi:10.1111/epi.14028 (2018).

21	Möller, C. *et al.* Impact of repeated kindled seizures on heart rate rhythms, heart rate variability, and locomotor activity in rats. *Epilepsy Behav* **92**, 36-44, doi:10.1016/j.yebeh.2018.11.034 (2019).

22	Buchecker, V. *et al.* Toward Evidence-Based Severity Assessment in Mouse Models with Repeated Seizures: (II.) Impact of Surgery and Intrahippocampal Kainate. *Eur Surg Res* **64**, 89-107, doi:10.1159/000522156 (2023).

23	Reiber, M., Miljanovic, N., Schonhoff, K., Palme, R. & Potschka, H. Behavioral phenotyping of young Scn1a haploinsufficient mice. *Epilepsy Behav* **136**, 108903, doi:10.1016/j.yebeh.2022.108903 (2022).

24	Reiber, M. *et al.* Phenotyping Young GluA1 Deficient Mice - A Behavioral Characterization in a Genetic Loss-of-Function Model. *Front Behav Neurosci* **16**, 877094, doi:10.3389/fnbeh.2022.877094 (2022).

25	Munk, A. *et al.* Refining pain management in mice by comparing multimodal analgesia and NSAID monotherapy for neurosurgical procedures. *Sci Rep* **14**, 18691, doi:10.1038/s41598-024-69075-2 (2024).

26	Koska, I. *et al.* Toward evidence-based severity assessment in rat models with repeated seizures: II. Chemical post-status epilepticus model. *Epilepsia* **60**, 2114-2127, doi:10.1111/epi.16330 (2019).

27	Di Liberto, V. *et al.* Imaging correlates of behavioral impairments: An experimental PET study in the rat pilocarpine epilepsy model. *Neurobiol Dis* **118**, 9-21, doi:10.1016/j.nbd.2018.06.010 (2018).

28	Seiffert, I. *et al.* Toward evidence-based severity assessment in rat models with repeated seizures: III. Electrical post-status epilepticus model. *Epilepsia* **60**, 1539-1551, doi:10.1111/epi.16095 (2019).

29	van Dijk, R. M. *et al.* Imaging biomarkers of behavioral impairments: A pilot micro-positron emission tomographic study in a rat electrical post-status epilepticus model. *Epilepsia* **59**, 2194-2205, doi:10.1111/epi.14586 (2018).

30	Aulehner, K. *et al.* The impact of tethered recording techniques on activity and sleep patterns in rats. *Sci Rep* **12**, 3179, doi:10.1038/s41598-022-06307-3 (2022).

31	Riedesel, A. K. *et al.* Severity Assessment of Complex and Repeated Intracranial Surgery in Rats. *Eur Surg Res* **64**, 108-119, doi:10.1159/000520678 (2023).

32	Johne, M. *et al.* Processing of auditory information in forebrain regions after hearing loss in adulthood: Behavioral and electrophysiological studies in a rat model. *Front Neurosci* **16**, 966568, doi:10.3389/fnins.2022.966568 (2022).

33	Jelinek, J. *et al.* Hearing loss in juvenile rats leads to excessive play fighting and hyperactivity, mild cognitive deficits and altered neuronal activity in the prefrontal cortex. *Curr Res Neurobiol* **6**, 100124, doi:10.1016/j.crneur.2024.100124 (2024).

34	Stenzel, M. *et al.* Exploring the cognitive effects of hearing loss in adult rats: Implications for visuospatial attention, social behavior, and prefrontal neural activity. *Neuroscience* **564**, 97-109, doi:10.1016/j.neuroscience.2024.11.010 (2025).

35	Helgers, S. O. A. *et al.* Lesion of the Fastigial Nucleus in Juvenile Rats Deteriorates Rat Behavior in Adulthood, Accompanied by Altered Neuronal Activity in the Medial Prefrontal Cortex. *Neuroscience* **442**, 29-40, doi:10.1016/j.neuroscience.2020.06.035 (2020).

36	Helgers, S. O. A. *et al.* Body weight algorithm predicts humane endpoint in an intracranial rat glioma model. *Sci Rep* **10**, 9020, doi:10.1038/s41598-020-65783-7 (2020).

37	Kumstel, S. *et al.* Benefits of non-invasive methods compared to telemetry for distress analysis in a murine model of pancreatic cancer. *J Adv Res* **21**, 35-47, doi:10.1016/j.jare.2019.09.002 (2020).
